# Integrated Analysis of Distant Metastasis-Associated Genes and Potential Drugs in Colon Adenocarcinoma

**DOI:** 10.3389/fonc.2020.576615

**Published:** 2020-10-23

**Authors:** Miaowei Wu, Weiyang Lou, Meng Lou, Peifen Fu, Xiao-Fang Yu

**Affiliations:** ^1^Cancer Institute, Second Affiliated Hospital, School of Medicine, Zhejiang University, Hangzhou, China; ^2^Department of Breast Surgery, First Affiliated Hospital of Zhejiang University, College of Medicine, Zhejiang University, Hangzhou, China

**Keywords:** colon adenocarcinoma, metastasis, survival, drug, bioinformatics analysis

## Abstract

**Background:** Most colon adenocarcinoma (COAD) patients die of distant metastasis, though there are some therapies for metastatic COAD. However, the genes exclusively expressed in metastatic COAD remain unclear. This study aims to identify prognosis-related genes associated with distant metastasis and develop therapeutic strategies for COAD patients.

**Methods:** Transcriptomic data from The Cancer Genome Atlas (TCGA; *n* = 514) cohort were analyzed as a discovery dataset. Next, the data from the GEPIA database and PROGgeneV2 database were used to validate our analysis. Key genes were identified based on the differential miRNA and mRNA expression with respect to M stage. The potential drugs targeting candidate differentially expressed genes (DEGs) were also investigated.

**Results:** A total of 127 significantly DEGs in patients with distant metastasis compared with patients without distant metastasis were identified. Then, four prognosis-related genes (LEP, DLX2, CLSTN2, and REG3A) were selected based on clustering analysis and survival analysis. Finally, three compounds targeting the candidate DEGs, including ajmaline, TTNPB, and dydrogesterone, were predicted to be potential drugs for COAD.

**Conclusions:** This study revealed that distant metastasis in COAD is associated with a specific group of genes, and three existing drugs may suppress the distant metastasis of COAD.

## Introduction

Distant metastasis accounts for a major proportion of cancer-related mortality, including colon carcinoma, which makes up to 10% of the global burden of cancer (1). Colon adenocarcinoma (COAD) is the most common histological subtype of colon carcinoma and has a poor prognosis (2). To date, metastatic colon carcinoma is uncurable in most cases, though there are some treatment options for metastatic disease, such as fluoropyrimidine-based chemotherapy, anti-VEGF agents, anti-EGFR drugs, and immunotherapy (3). Previous studies of COAD focused on the prognostic models and regulators of metastasis (4, 5). However, the genes exclusively expressed in metastatic COAD have yet to provide novel biomarkers and drug targets for use in the clinic.

Differences in the gene expression spectrum between primary and metastatic COAD, which are important for finding cancer biomarkers, have not been thoroughly evaluated. Recent advances in colon carcinoma highlight the urgent need for molecular markers and therapeutic targets in the management of metastatic cancer (6, 7). Here, we screened the differentially expressed gene (DEG) profiles of primary tumor from COAD patients with distant metastasis (M1) compared with those of patients without distant metastasis (M0) in The Cancer Genome Atlas (TCGA) database. Finally, we identified and validated four prognosis-related genes as well as three potential drugs for COAD based on bioinformatics analysis.

## Materials and Methods

### The TCGA Cohort

The RNA-Seq gene expression profiles (Counts format) of COAD patients were downloaded from the TCGA database (https://portal.gdc.cancer.gov/) in March 2020. Clinical data, such as gender, age, tumor grade, clinical stage, and survival time, were also downloaded from the TCGA portal. Next, according to the American Joint Committee on Cancer (AJCC) staging system, we enrolled COAD samples that pathologic M Stage were M0 (*n* = 334) and M1 (*n* = 62), while COAD samples that pathologic M Stage were MX or missing were excluded. Then, R software was used for data extraction and sorting to obtain the gene expression matrices and clinical data.

### Screening the Differentially Expressed miRNAs (DEmiRNAs) and DEGs

The miRNA differential expression analysis was conducted using the “edgeR” package in Bioconductor (https://bioconductor.org/). The related codes were run in R, and the DEmiRNAs and DEGs in the primary tumors of patients with distant metastasis (M1) compared with those of patients without distant metastasis (M0) were analyzed through the “edgeR” package. *P* < 0.05 and |fold change (FC)| > 1 were set as the thresholds for identifying DE-miRNAs and DEGs. The package “pheatmap” was used for clustering and heat map construction.

### Predicting the Target Genes of DEmiRNAs

The target genes of the DEmiRNAs were identified using mirDIP v4.1 (http://ophid.utoronto.ca/mirDIP/index.jsp), an integrative database containing nearly 152 million human microRNA-target predictions, which were collected across 30 different resources (8). Then, the common DEGs identified by the above two methods were presented as Venn diagrams and identified as candidate DEGs.

### Protein-Protein Interaction (PPI) Network and miRNA-Gene Network Construction

The PPI network and miRNA-gene network were successively constructed. The candidate DEGs were first mapped to the Search Tool for the Retrieval of Interacting Genes/Proteins (STRING) database (http://string-db.org) to assess the functional associations among the target genes (9). Only interactions with a combined score of > 0.4 were considered significant. Next, to obtain the hub genes, the degree of connectivity in the PPI network was analyzed by Cytoscape software (version 3.6.0) (10), after which the top 30 hub genes were screened and their PPI subnetwork was established.

To establish the regulatory network of DEmiRNAs and hub genes, first, the upregulated DEmiRNAs and the downregulated hub genes were mapped based on the mirDIP database. In the same way, the downregulated DEmiRNAs and the upregulated hub genes were mapped. Based on this, two miRNA-hub gene regulatory networks were constructed using Cytoscape software (version 3.6.0).

### Screening Prognosis-Related Genes

The Gene Expression Profiling Interactive Analysis (GEPIA) database (http://gepia2.cancer-pku.cn/), an interactive web server for analyzing the RNA-Seq expression data from the TCGA and GTEx projects, was used to analyze the prognostic significance of the screened candidate DEGs in COAD (11). In addition, the PROGgeneV2 database (http://www.compbio.iupui.edu/proggene), a web application for analyzing the prognostic implications of mRNA biomarkers in 18 cancers that combines public repositories such as Gene Expression Omnibus (GEO), European Bioinformatics Institute (EBI) ArrayExpress, and TCGA (12), was used to study the prognostic relevance of the key genes associated with distant metastasis. Furthermore, the package “survival” package was used to plot the Kaplan-Meier survival curves of the TCGA-COAD cohort. In survival analysis, patients were divided into low-expression and high-expression groups based on the median value of the corresponding genes expression.

DIANA-miRPath (v3.0; http://www.microrna.gr/miRPathv3) was used to identify the Kyoto Encyclopedia of Genes and Genomes (KEGG) pathways associated with the DEmiRNAs (13). Metascape (https://metascape.org/), an integrated portal providing a comprehensive gene list annotation and analysis resource by combining functional enrichment, interactome analysis, gene annotation, and membership search based on over 40 independent knowledge bases (14), was used to analyze the enriched terms (Gene Ontology (GO)/KEGG terms, canonical pathways, hallmark gene sets, etc.) of the candidate DEGs. Additionally, the AmiGO database (v2.0; http://amigo.geneontology.org/amigo/) was used to analyze the GO terms of the prognosis-related genes (15).

Next, the Comparative Toxicogenomics Database (CTD) (http://ctdbase.org/) was used to find integrated chemical-gene, chemical-disease, and gene-disease interactions to generate expanded networks and predict novel associations (16). Here, relationships between prognosis-related genes and diseases were investigated.

### Survival Curve and Risk Score

After prognosis-related genes were detected, we used step multivariate Cox regression analysis in the “survival” package to build a gene signature panel. Patients were divided into high- and low-risk groups using the median risk score, and Kaplan-Meier curves were used to explore overall survival (OS) of the two groups using the “survminer” package. The survival receiver operating characteristic curve (ROC) was drawn and the area under the curve (AUC) was calculated for evaluating prognostic value.

### Prediction of Novel Drugs for COAD

The Connectivity Map (CMap) (https://portals.broadinstitute.org/cmap/) (17) is a platform for finding connections among small molecules that share the same mechanism of action and associations with chemicals and physiological processes, diseases, and drugs. In the present study, the candidate DEGs were divided into two groups: up- and downregulated genes. The upregulated and downregulated genes were subsequently uploaded to query CMap, and searches were made for small-molecule compounds. Scores ranged from −1 to 1, representing the relationship between the drugs and input genes, and drugs with a score of ≤ −0.75 (*p* < 0.05, percent non-null > 50) were considered candidate drugs.

Then, the above results were validated using the CTD (http://ctdbase.org/), which contains associations among chemicals, gene products, and diseases (16).

### ONCOMINE Analysis

ONCOMINE gene expression array datasets (https://www.oncomine.org/), an online cancer microarray database containing 715 gene expression data sets and data from 36 86,733 cancerous and normal tissues, was used to analyze the transcription levels of the four identified genes in colorectal cancer. The mRNA expressions of LEP, DLX2, CLSTN2, and REG3A in colorectal cancer specimens were compared with that in normal controls, using a Student's *t*-test to generate a *p*-value.

### Tumor Immune Estimation Resource (TIMER) Database Analysis

The Tumor Immune Estimation Resource (TIMER) (18) is an integrative web server for evaluating tumor-infiltrating immune cells across diverse cancer types (https://cistrome.shinyapps.io/timer/). The TIMER includes more than 10,000 samples across multiple cancer types of the TCGA. It applies a partial deconvolution linear least square regression method to calculate the abundance of immune infiltrates. We investigated the correlations between the identified genes expression and immune infiltrates in tumors, including CD8+ T cells, CD4+ T cells, dendritic cells, neutrophils, B cells, and macrophages. The Correlation module was used to draw the expression scatterplots comparing a pair of user-defined genes in COAD (*n* = 457), together with the Spearman's correlation and estimated statistical significance. The identified genes were represented on the y-axis with gene symbols, whereas the metastasis-related immune markers were placed on the x-axis. The gene expression levels were determined with log2 RSEM. Options used for partial correlation conditioned on tumor purity were also used.

## Results

**Figure 1:**
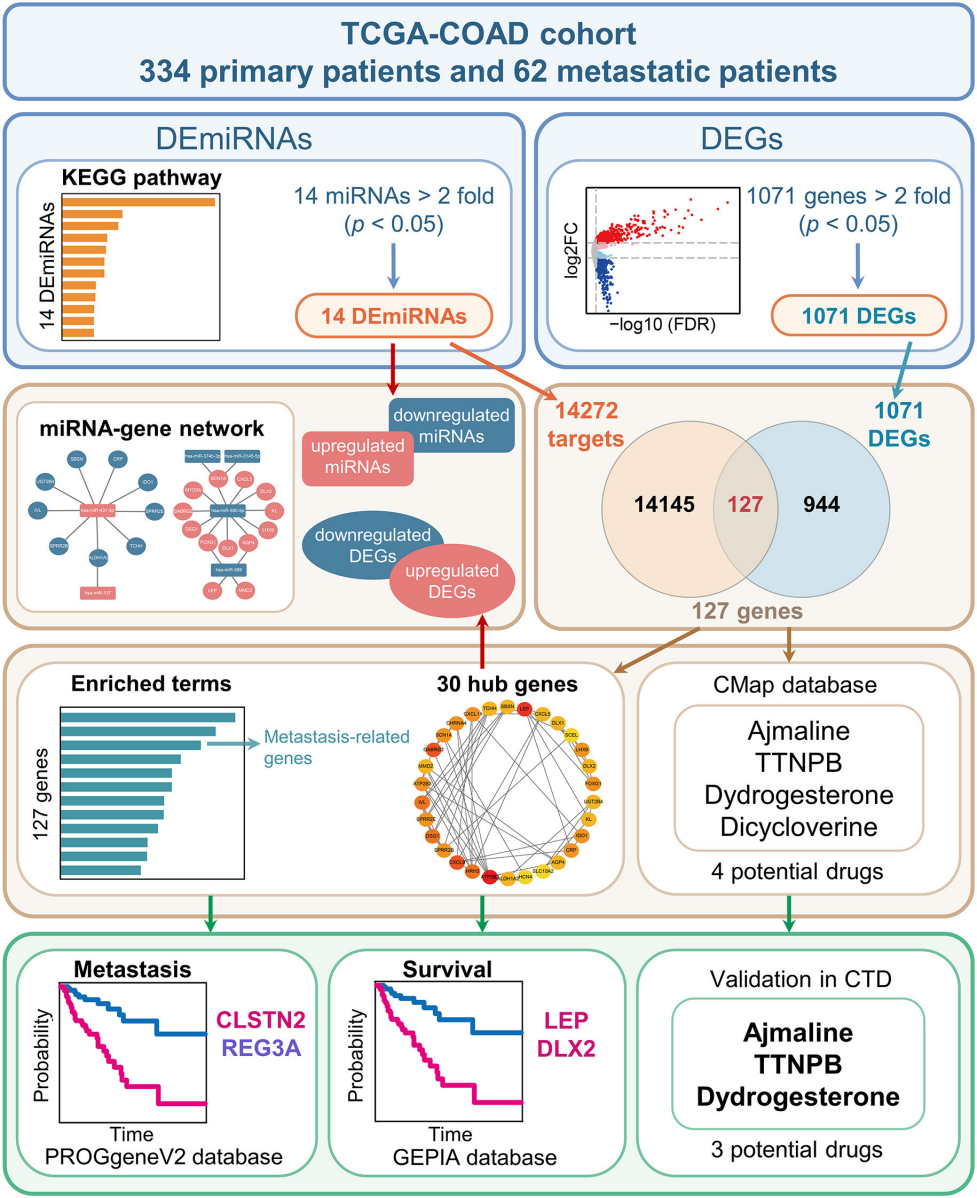
Schematic diagram of the study design. The TCGA-COAD cohort was used to identify the differentially expressed miRNAs (DEmiRNAs) and genes (DEGs) between primary COAD patients and metastatic COAD patients. In brief, 14 miRNAs and 1,071 genes were identified as DEmiRNAs and DEGs, respectively. Subsequently, 14,272 genes were identified as targets of the 14 DEmiRNAs. The 14,272 targets and 1,071 DEGs were overlapped. Next, the common gene set consisting of 127 genes were analyzed. As a result, four candidate genes associated with prognosis were obtained by combining the top 30 hub genes with genes involved in metastasis-related processes. Additionally, three potential small-molecule drugs for COAD were predicted using the CMap database and further validated in CTD.

To explore the prognostic markers specifically expressed in metastatic COAD and novel therapeutic strategies, the present study was designed and analyzed according to the flow chart shown in Figure 1. The patients' clinicopathological characteristics of are listed in Table 1. The specific analysis processes are shown in detail in the following figures and tables.

**Table 1:** Clinicopathological characteristics of COAD patients.

**Variables**	**Non-distant metastatic** **(*n* = 334)**	**Distant metastatic** **(*n* = 62)**
Average Patient Age, years (range)	67.83 (31-90)	64.15 (35-85)
**Sex**		
Male	176	32
Female	158	30
**Histological type**		
Adenocarcinoma, NOS	283	54
Mucinous adenocarcinoma	46	7
Others	5	1
**Pathologic tumor stage (AJCC)**		
I	67	0
II	161	0
III	103	0
IV	0	62
**Pathologic T stage (AJCC)**		
T1	8	0
T2	67	0
T3	236	38
T4	23	24
**Pathologic N stage (AJCC)**		
N0	231	8
N1	64	25
N2	39	29
**Pathologic M stage (AJCC)**		
M0	334	0
M1	0	62

*COAD, colon adenocarcinoma; NOS, not otherwise specified; AJCC, American Joint Committee on Cancer*.

### DEmiRNAs and Their Target Genes

**Figure 2:**
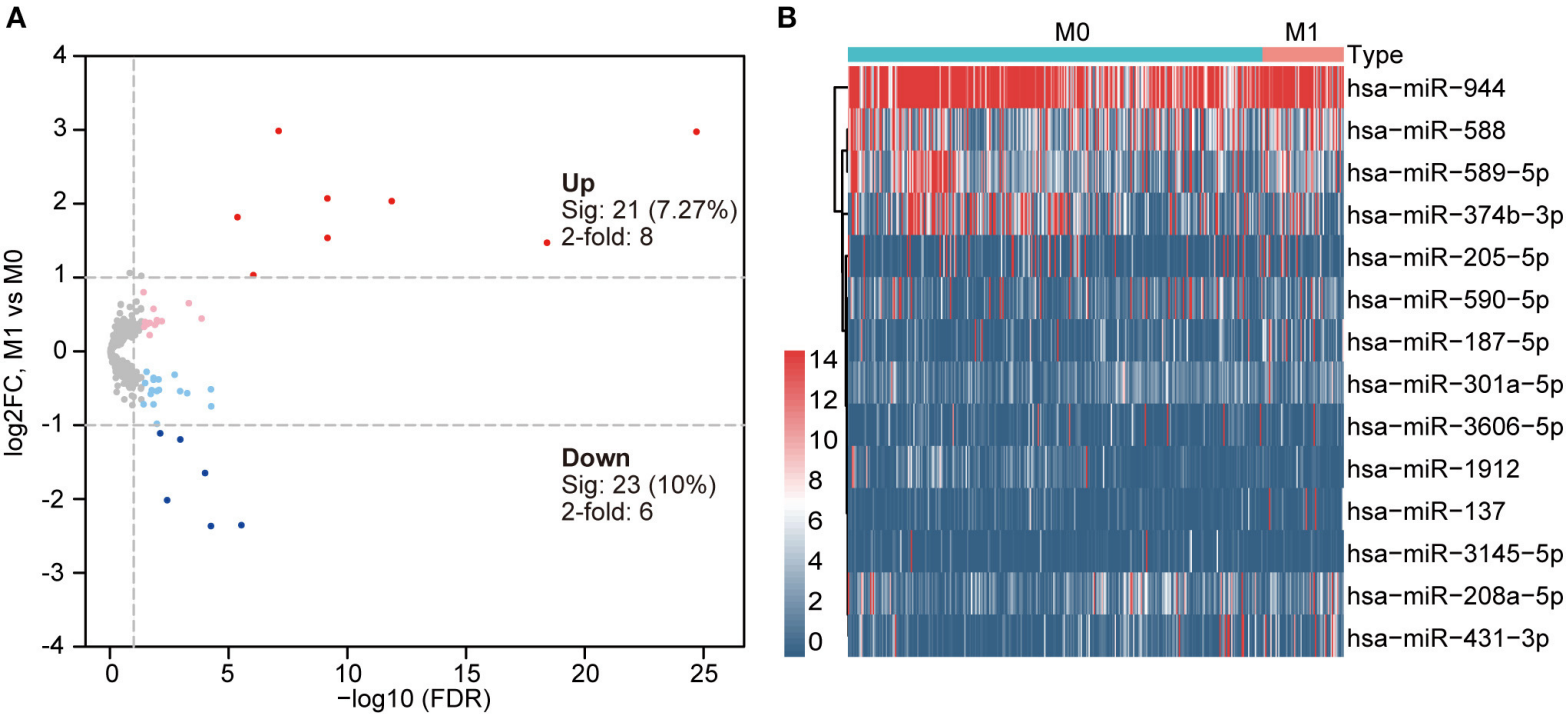
**(A)** Volcano plot indicating differentially expressed miRNAs (DEmiRNAs) in primary COAD patients and metastatic COAD patients (light red and blue colors indicate adj. *p* < 0.05 (sig), whereas red and blue further require more than a 2-fold change); other genes are colored in gray. **(B)** Heat map of DEmiRNAs in primary COAD patients and metastatic COAD patients. Red, higher expression. Blue, lower expression.

To screen DEmiRNAs from the miRNA-Seq dataset of the TCGA cohort, we conducted a differential expression analysis using the “edgeR” software package. In this cohort, 62/379 (16.4%) of the DEmiRNAs were from patients with stage M1, and 317/379 (83.6%) were from patients with stage M0. Then, 14 DEmiRNAs with a critical point of |log2FC > 1| and *p* < 0.05, were obtained, including 8 upregulated DEmiRNAs and 6 downregulated DEmiRNAs (Table 2). The heat map and volcano plot are shown in Figure 2. Additionally, 8,918 potential target genes for the 8 upregulated miRNAs and 5,354 potential target genes for the 6 downregulated miRNAs were determined by using the mirDIP database.

**Table 2:** Identification of differentially expressed miRNAs (DEmiRNAs) in TCGA-COAD patients with or without distant metastasis.

**DEmiRNAs**	**Log2FC**	***p-*value**	**FDR**
hsa-miR-205-5p	2.983302	9.58E-10	8.29E-08
hsa-miR-137	2.972022	4.02E-28	2.09E-25
hsa-miR-431-3p	2.068959	7.05E-12	7.32E-10
hsa-miR-187-5p	2.032728	8.29E-15	1.43E-12
hsa-miR-3606-5p	1.815279	7.79E-08	4.49E-06
hsa-miR-208a-5p	1.534453	5.60E-12	7.27E-10
hsa-miR-944	1.469469	1.59E-21	4.12E-19
hsa-miR-301a-5p	1.030107	1.30E-08	9.63E-07
hsa-miR-374b-3p	−1.11184	0.000342	0.008068
hsa-miR-1912	−1.19534	3.76E-05	0.001148
hsa-miR-589-5p	−1.64981	2.61E-06	0.000104
hsa-miR-3145-5p	−2.01733	0.000159	0.004113
hsa-miR-588	−2.35543	4.73E-08	3.07E-06
hsa-miR-590-5p	−2.36745	1.27E-06	5.89E-05

*COAD, colon adenocarcinoma*.

### Identification of DEGs

**Figure 3:**
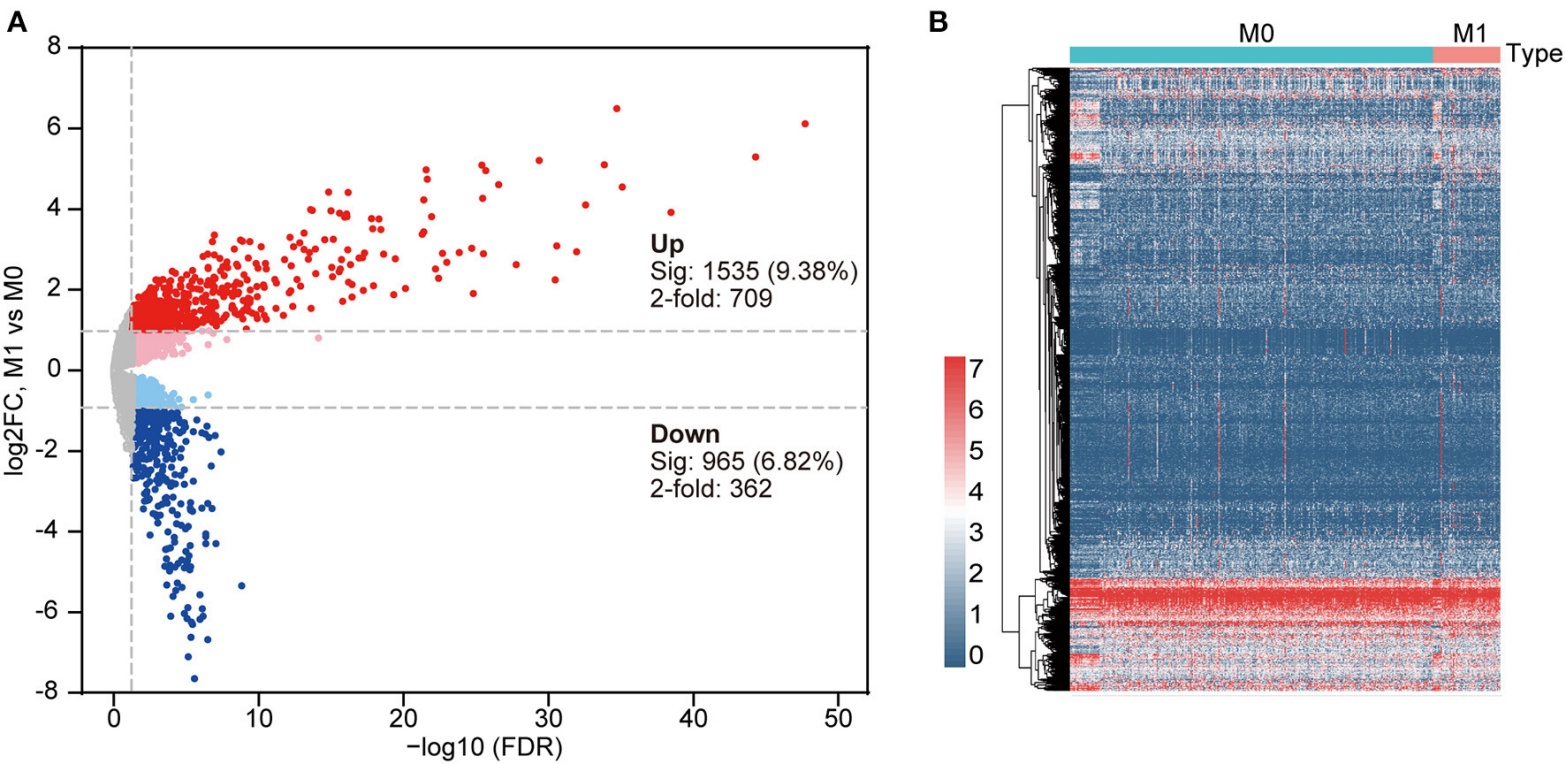
**(A)** Volcano plot indicating differentially expressed genes (DEGs) in primary COAD patients and metastatic COAD patients (light red and blue colors indicate adj. *p* < 0.05 (sig), whereas red and blue further require more than a 2-fold change); other genes are colored in gray. **(B)** Clustered heat map of DEGs in primary COAD patients and metastatic COAD patients. Red, higher expression. Blue, lower expression.

**Figure 4:**
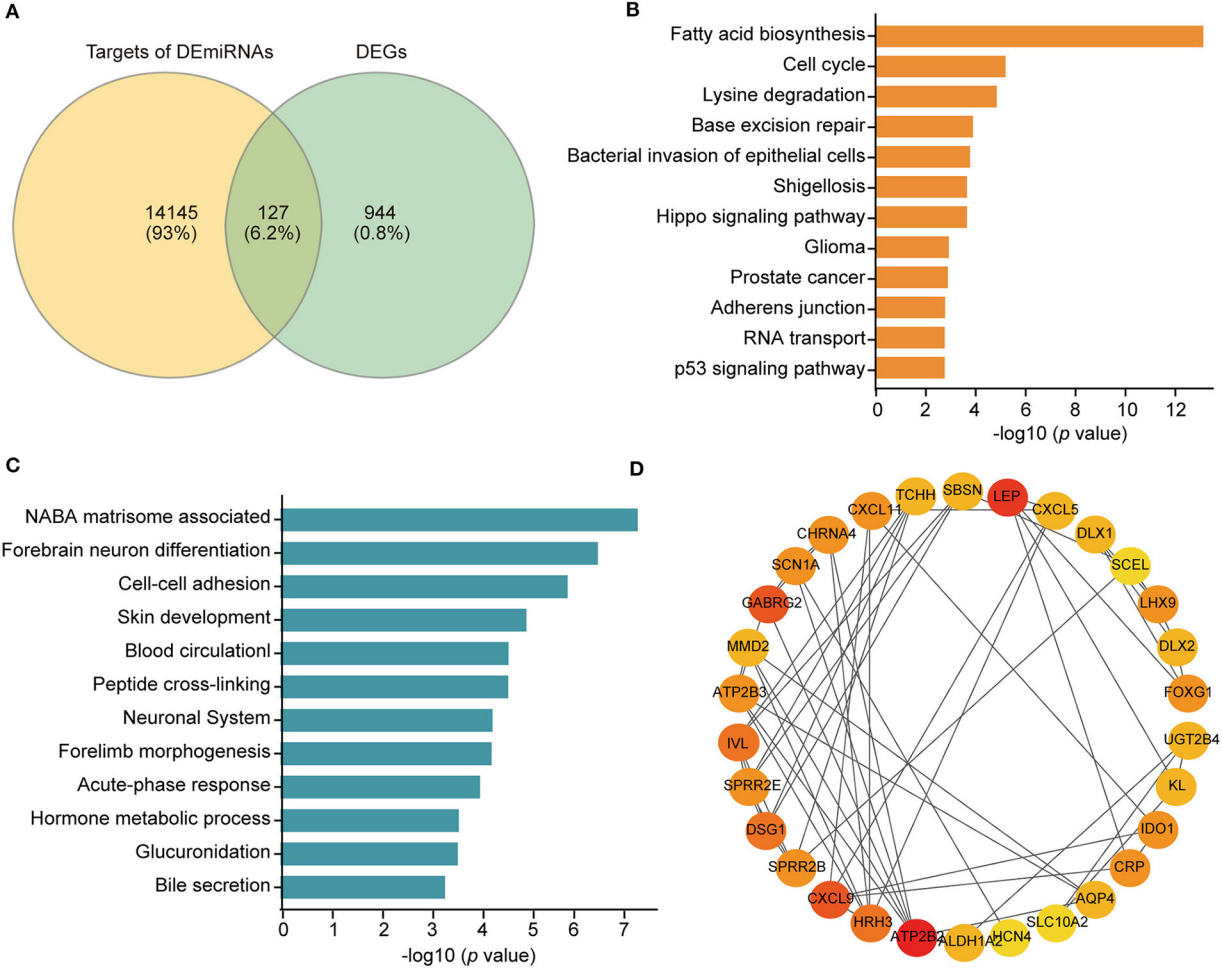
**(A)** Venn diagram depicting the overlap between the targets of the DEmiRNAs and DEGs. **(B)** Kyoto Encyclopedia of Genes and Genomes (KEGG) pathway enrichment of 14 DEmiRNAs based on the DIANA database. **(C)** Enriched ontology clusters of the 127 genes overlapping the targets of the DEmiRNAs and DEGs based on the Metascpae database. **(D)** The top 30 genes evaluated by connectivity degree in the PPI network of the 127 genes. The color from red to yellow represents the connectivity degree from high to low.

To acquire DEGs from the mRNA-Seq dataset of the TCGA cohort, we conducted a differential expression analysis using the “edgeR” software package. In this cohort, 61/391 (15.6%) of the DEGs were from patients with stage M1, and 330/391 (84.4%) were from patients with stage M0. Then, 1,071 DEGs with a critical point of |log2FC > 1| and *p* < 0.05 were identified. Among these DEGs, 709 DEGs were upregulated and 362 DEGs were downregulated in the metastatic group (Supplementary Table 1). The heat map and volcano plot are displayed in Figure 3. The overlap between the targets of the DEmiRNAs and the DEGs was determined, revealing 127 shared genes (Figure 4A). We focused our analysis on these 127 genes, which were defined as candidate DEGs (Supplementary Table 2).

### Functional Annotation and Pathway Enrichment Analysis

To analyze the enriched pathways of the DEmiRNAs, we carried out KEGG pathway enrichment analysis using the DIANA bioinformatic tool. The 14 DEmiRNAs were significantly enriched in cancer and metastasis-related pathways, such as the cell cycle, adherens junction, and p53 signaling pathway (Figure 4B).

Moreover, pathway and process enrichment analyses were conducted based on the 127 candidate DEGs. The top 12 involved clusters were “NABA matrisome associated,” “forebrain neuron differentiation,” “cell-cell adhesion via plasma-membrane adhesion molecules,” “skin development,” “blood circulation,” “peptide cross-linking,” “neuronal system,” “forelimb morphogenesis,” “acute-phase response,” “hormone metabolic process,” “glucuronidation,” and “bile secretion” (Figure 4C). Specifically, among these candidate DEGs, 10 genes participated in cell-cell adhesion *via* the plasma membrane adhesion molecule biological process, including DSG1, CDH9, SLITRK1, CLDN19, PCDHA9, PCDH11Y, PCDHGB7, CLSTN2, REG3A, and CLDN18, which were considered potential metastasis-related DEGs.

### Construction of the PPI Network and Regulatory miRNA-Hub Gene Networks

The PPI network of COAD metastasis-associated genes was constructed using the STRING database. Data from this database suggested that most of these genes could interact with each other. The top 30 hub genes were screened out according to the node degree (Figure 4D). For the upregulated genes, the hub genes were ATP2B3, MMD2, FOXG1, LEP, DSG1, GABRG2, MYO3A, KL, CXCL5, DLX2, SCN1A, LHX9, AQP4, DLX1, ATP2B2, HRH3, ASTN1, SLITRK1, and CHRNA4. For the downregulated genes, the hub genes were CXCL11, CXCL9, UGT2B4, SBSN, CRP, IDO1, TCHH, SPRR2E, SPRR2B, IVL, and ALDH1A2.

**Figure 5:**
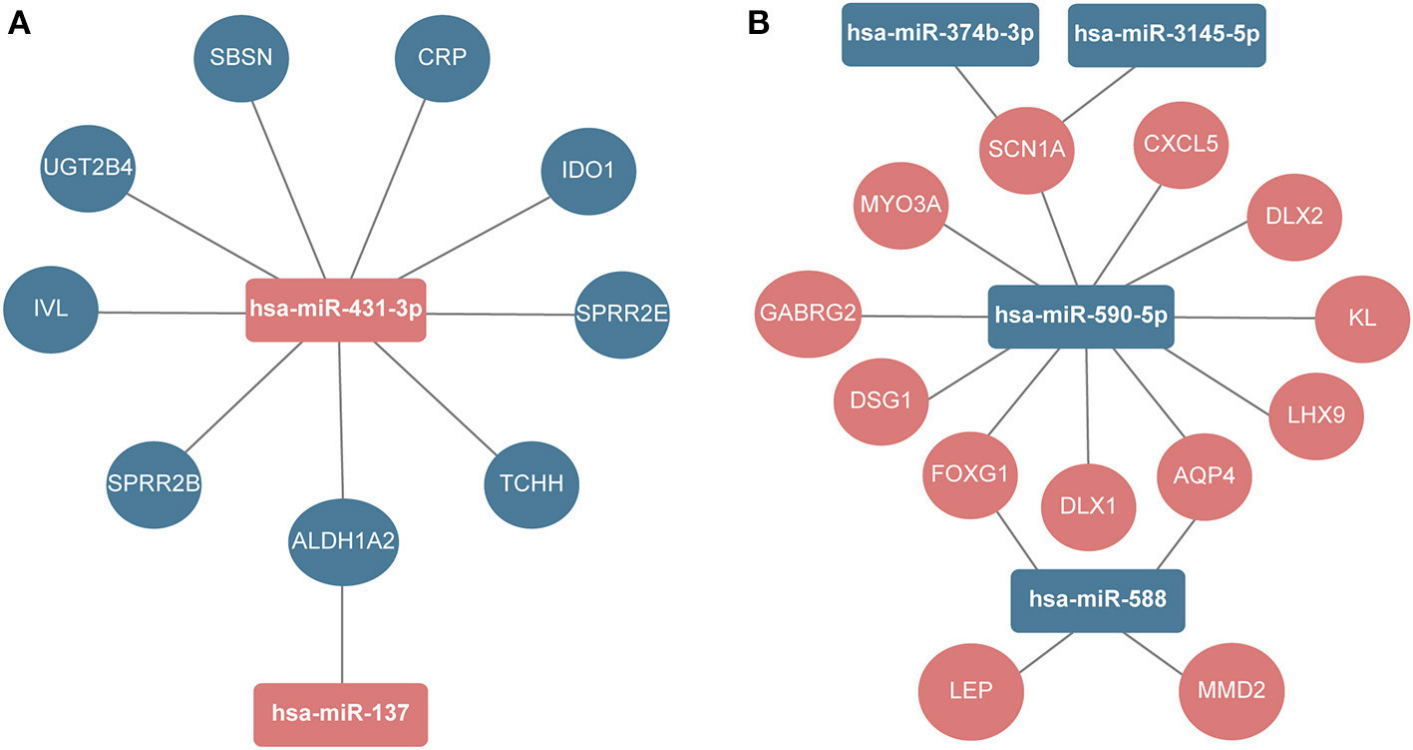
The regulatory network between DEmiRNAs and hub genes. **(A)** Upregulated miRNAs. **(B)** Downregulated miRNAs.

Next, the miRNA-hub gene networks were constructed using Cytoscape (Figure 5). Among the top 30 hub genes, we found that nine hub genes (IVL, UGT3B4, IDO1, CRP, TCHH, ALDH1A2, SPRR2B, SPRR2E, and SBSN) could be potentially modulated by upregulated miR-431-3p, while ALDH1A2 could also be potentially modulated by upregulated miR-137. Moreover, 11 hub genes could be regulated by downregulated miR-590-5p, including MYO3A, CXCL5, LHX9, SCN1A, DSG1, DLX2, DLX1, AQP4, FOXG1, GABRG2, and KL. Additionally, SCN1A could be potentially modulated by the downregulation of miR-3145-5p and miR-374b-3p. Moreover, downregulated miR-588 could potentially modulate four hub genes, including AQP4, FOXG1, LEP, and MMD2.

### Determination of Key Genes Correlated With Prognosis in COAD

**Figure 6:**
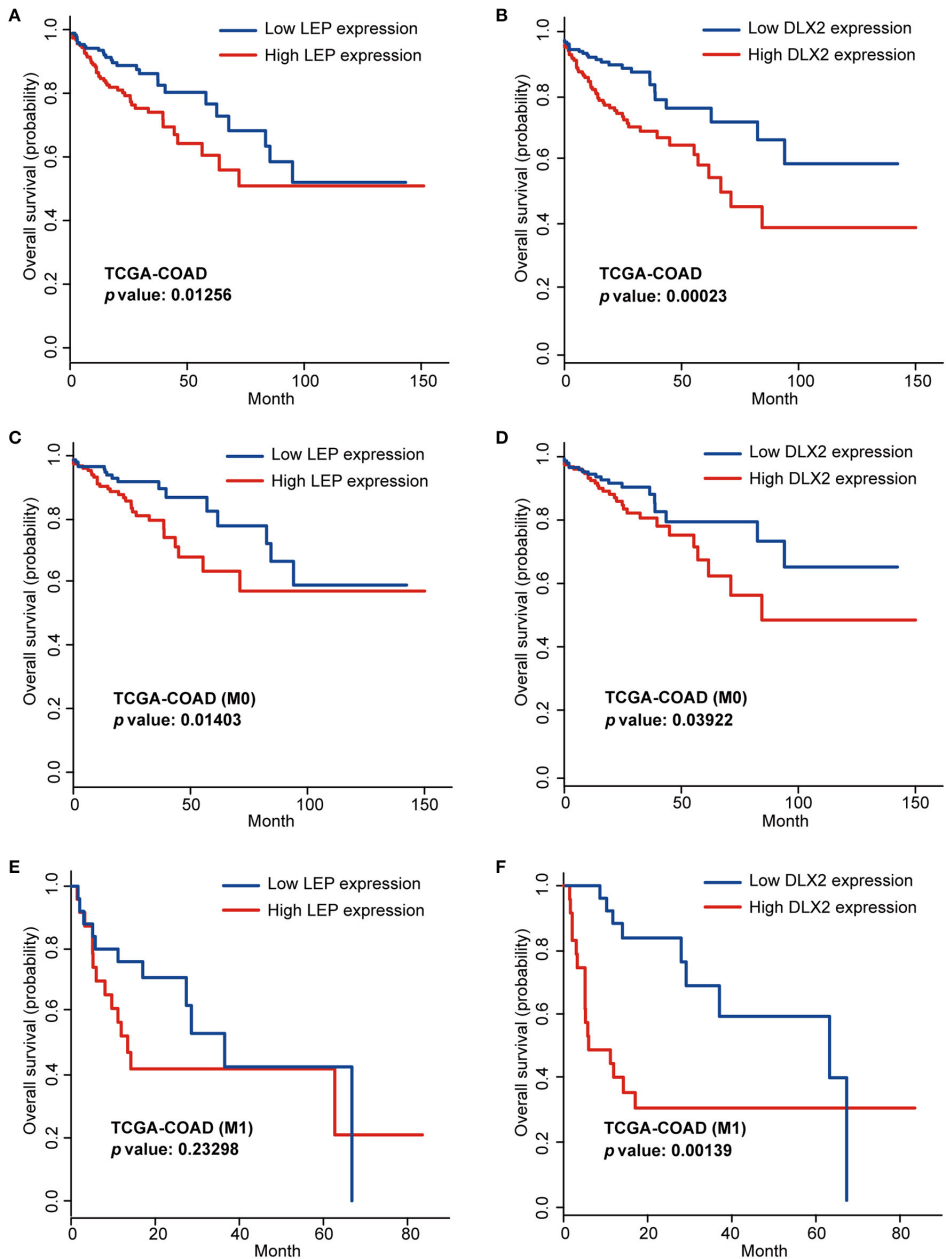
Kaplan-Meier analysis of LEP **(A)** and DLX2 **(B)** in the TCGA-COAD cohort. Kaplan-Meier analysis of LEP **(C)** and DLX2 **(D)** in the M0 stage TCGA-COAD cohort. Kaplan-Meier analysis of LEP **(E)** and DLX2 **(F)** in the M1 stage TCGA-COAD cohort.

To screen the prognostic genes, Kaplan-Meier analysis was performed based on the expression levels of the hub genes in the TCGA-COAD cohort. Among the top 30 hub genes, we found that two genes (LEP and DLX2) were significantly associated with overall survival (OS), and these genes were considered risky genes, with hazard ratio (HR) > 1 (*p* < 0.05; Figures 6A,B). Furthermore, for patients with stage M0 disease, higher levels of LEP and DLX2 were markedly associated with worse OS (*p* < 0.05; Figures 6C,D). However, for patients with stage M1, the expression of LEP had no significant effect on OS (Figure 6E), while higher expression of DLX2 was significantly correlated with worse OS (*p* < 0.05; Figure 6F).

**Figure 7:**
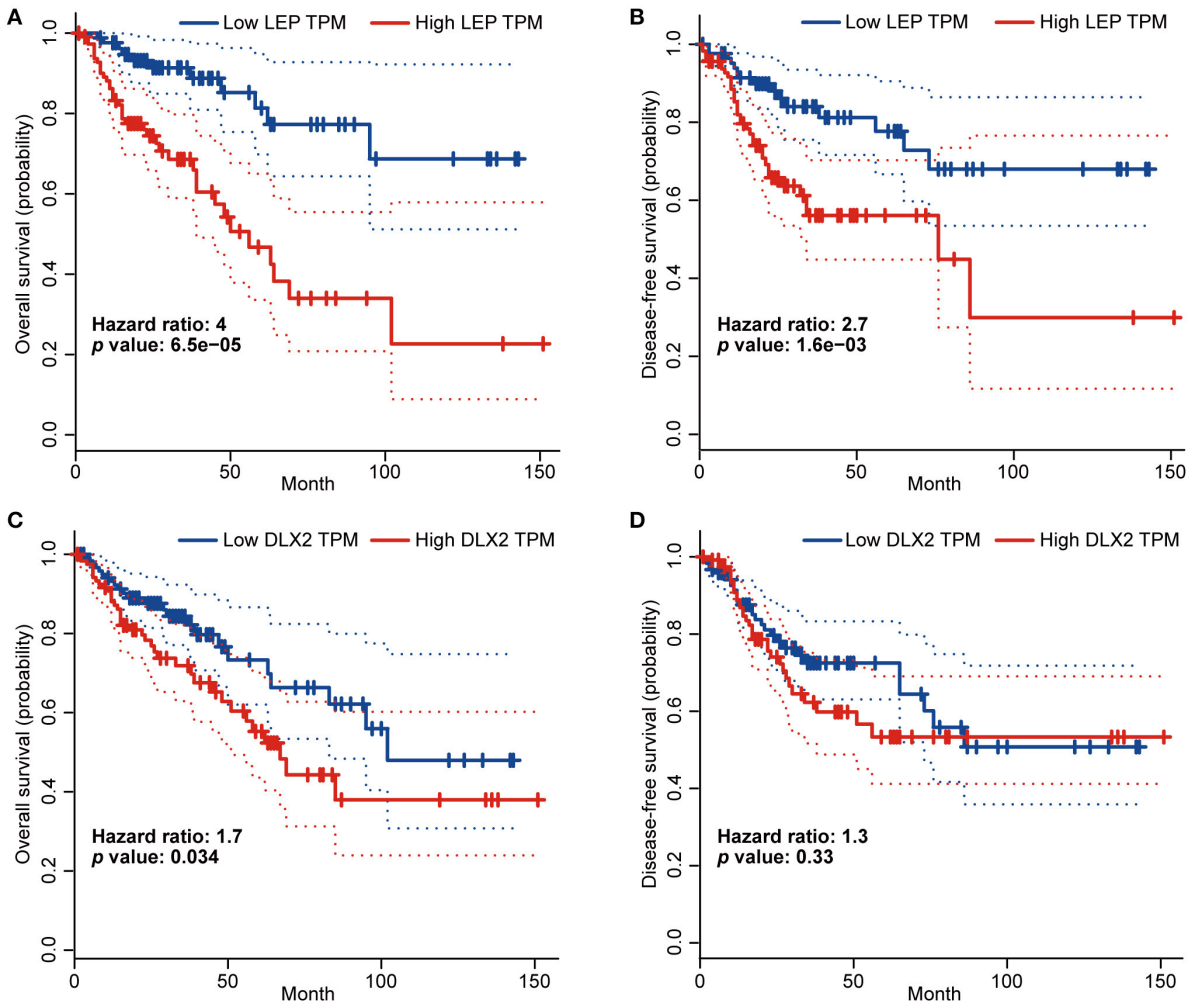
The prognostic values of LEP and DLX2 in COAD patients (GEPIA).The overall survival curve **(A)** and disease-free survival curve **(B)** of LEP. The overall survival curve **(C)** and disease-free survival curve **(D)** of DLX2.

We validated the prognostic values of LEP and DLX2 using the GEPIA database. The results showed that COAD patients with higher LEP expression had worse OS and disease-free survival (DFS) (*p* < 0.05; Figures 7A,B). In addition, COAD patients with higher DLX2 expression had worse OS (*p* < 0.05; Figure 7C), but DLX2 expression had no significant effect on DFS (Figure 7D).

**Figure 8:**
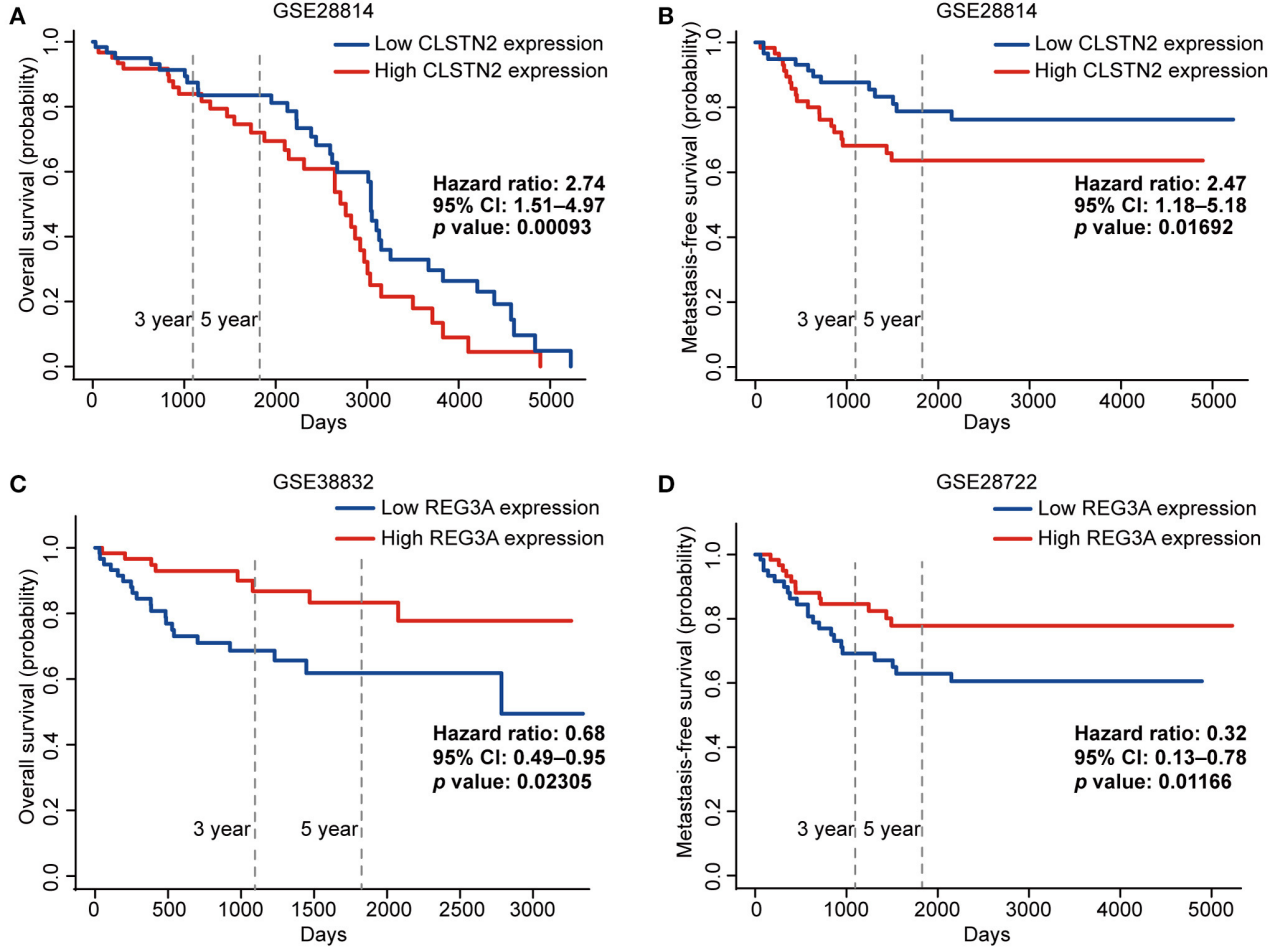
The prognostic values of CLSTN2 and REG3A in colorectal cancer patients (PROGgeneV2).The overall survival curve **(A)** and metastasis-free survival curve **(B)** of CLSTN2. The overall survival curve **(C)** and metastasis-free survival curve **(D)** of REG3A.

Subsequently, we further investigated the prognostic roles of the above 10 metastasis-related DEGs in colorectal cancer patients. Higher expression of CLSTN2 was markedly correlated with worse OS and metastasis-free survival (MFS) (*p* < 0.05; Figures 8A,B). In contrast, higher expression of REG3A was significantly associated with better OS and MFS (*p* < 0.05; Figures 8C,D).

### Risk Score and Survival Analysis

Next, we compared the performance of identified individual genes with the combined gene signature panel. First, we used step multivariate Cox regression analysis to establish a gene signature with prognostic value for COAD. According to the results, the risk scoring system for each patient was calculated as follows: risk score = LEP * 0.134582 + REG3A * (−0.05402) + DLX2 * 0.211469, after which the patients were classified into a low-risk (*n* = 178) or a high-risk (*n* = 178) group based on the median risk score. As shown in Supplementary Figure 1A, patients in high-risk group had significantly poorer survival compared with those in low-risk group (log-rank test, *p* < 0.05). Subsequently, to evaluate the prognostic value of risk score, we drew the ROC curve at 5 years of OS and calculated the AUC value. From Supplementary Figure 1B, the 5 year AUC was 0.672 (AUC > 0.65), which revealed superior predictive accuracy in OS (19).

Second, for the individual genes, the correlations between their expression levels and prognosis are shown in Figure 6. Similarly, the ROC curves at 5 years of OS were plotted based on the risk score of each sample. Additionally, the 5 year AUC of LEP, DLX2, CLSTN2, and REG3A were 0.638, 0.622, 0.5, and 0.62, respectively (AUC < 0.65), indicating that the combined gene signature panel have better performance on evaluating the prognosis of COAD patients.

### Functional Enrichment and Related Diseases of the Four Prognosis-Related Genes

Figures 6–8 show that the key genes, including LEP, DLX2, CLSTN2, and REG3A, are associated with the prognosis of COAD patients. To learn more about the four genes, we analyzed their GO term enrichment related to biological processes, molecular functions, and cellular components (Supplementary Table 3). Notably, both LEP and REG3A were involved in the regulation of the extracellular space. DLX2 participated in modulating DNA binding-related processes, transcription, and cell differentiation. CLSTN2 played a role in homophilic cell adhesion via plasma membrane adhesion molecules. Subsequently, the CTD was used to investigate the diseases related to these genes, and the results showed that all four genes are relevant to colonic neoplasms (Supplementary Table 4).

### The Four Prognosis-Related Genes Expressions Are Correlated With Immune Infiltration Level and Immune Markers in COAD

As tumor microenvironment, especially immune cells play an important role in metastasis (20, 21), we explored whether the four genes expressions are correlated with immune cells and immune-related markers in COAD.

**Figure 9:**
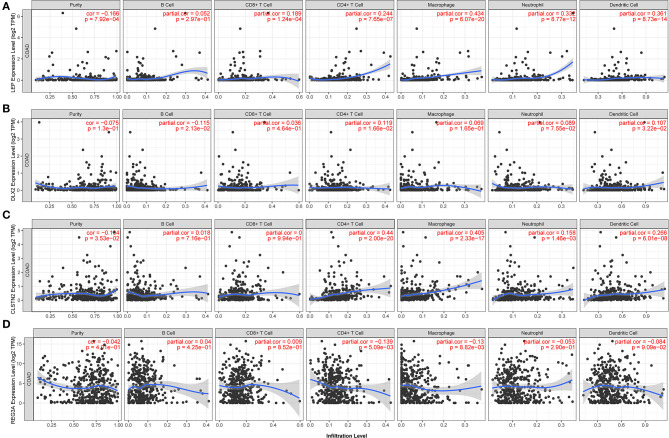
Correlations of the four prognosis-related genes with various immune cells in COAD (TIMER). The correlation between the abundance of immune cell and the expression of **(A)** LEP, **(B)** DLX2, **(C)** CLSTN2, and **(D)** REG3A. The x axis indicates the prognosis related genes and y axis indicates immune cell types. Each dot represents a sample, and the blue line represents the relationship between the expression level of each gene and immune cell contents.

As for immune cells, LEP expression level had significantly positive correlations with infiltrating levels of CD4+ T cells (*r* = 0.244, *p* = 7.65e-07), macrophages (*r* = 0.434, *p* = 6.07e-20), neutrophils (*r* = 0.332, *p* = 8.77e-12), and dendritic cells (DCs) (*r* = 0.361, *p* = 8.73e-14) (Figure 9A). In addition, there were weak correlations between DLX2 expression level and infiltrating levels of B cells, CD4+ T cells, and DCs (Figure 9B). Furthermore, there were significantly positive correlations between CLSTN2 expression level and infiltrating levels of CD4+ T cells (*r* = 0.44, *p* = 2.00e-20), macrophages (*r* = 0.405, *p* = 2.33e-17), and DCs (*r* = 0.266, *p* = 6.01e-08) (Figure 9C). On the other hand, REG3A expression level had weak negative correlations with infiltrating levels of CD4+ T cells and macrophages in COAD (Figure 9D).

As regard to metastasis-related immune markers (Table 3). After the correlation adjustment by purity, the results revealed that the LEP expression level was significantly positively correlated with 18 immune markers among 22 metastasis-related markers. DLX2 and CLSTN2 expression levels were significantly positively correlated with 11 and 16 immune markers, respectively. However, among 22 metastasis-related immune markers, REG3A expression level was significantly positively correlated with two immune markers and significantly negatively correlated with three markers in COAD.

**Table 3:** Correlation analysis between four identified genes and immune-related markers in COAD (TIMER).

**Gene markers**	**LEP**				**DLX2**				**CLSTN2**				**REG3A**			
	**None**		**Purity**		**None**		**Purity**		**None**		**Purity**		**None**		**Purity**	
	**Cor**	***p***	**Cor**	***p***	**Cor**	***p***	**Cor**	***p***	**Cor**	***p***	**Cor**	***p***	**Cor**	***p***	**Cor**	***p***
CCL2	0.455	^***^	0.438	^***^	0.067	0.154	0.036	0.469	0.378	^***^	0.360	^***^	−0.043	0.355	−0.048	0.331
TGFβ (TGFB1)	0.369	^***^	0.314	^***^	0.169	^***^	0.167	^***^	0.377	^***^	0.379	^***^	−0.076	0.106	−0.121	^*^
CTLA4	0.249	^***^	0.219	^***^	0.166	^***^	0.148	^**^	0.167	^***^	0.146	^**^	0.067	0.155	0.040	0.420
CCL5	0.266	^***^	0.249	^***^	0.025	0.591	0.021	0.667	0.029	0.542	−0.017	0.734	−0.057	0.222	−0.064	0.199
IL10	0.381	^***^	0.370	^***^	0.137	^**^	0.112	^*^	0.201	^***^	0.199	^***^	−0.021	0.654	−0.044	0.381
COX2(PTGS2)	0.289	^**^	0.133	^**^	0.154	^***^	0.139	^**^	0.075	0.111	0.043	0.389	0.111	^*^	0.097	0.051
VEGF (VEGFA)	0.162	0.154	−0.010	0.837	0.128	^**^	0.143	^**^	0.143	^**^	0.142	^**^	−0.157	^***^	−0.196	^***^
PD-1 (PDCD1)	0.217	^***^	0.187	^***^	0.133	^**^	0.136	^**^	0.082	0.081	0.047	0.344	0.027	0.565	0.020	0.695
PD-L1 (CD274)	−0.060	0.599	0.243	^***^	0.118	^*^	0.112	^*^	0.098	^*^	0.069	0.164	0.031	0.505	0.019	0.702
CXCL5	0.142	^**^	0.096	0.052	0.088	0.060	0.069	0.165	0.057	0.222	0.025	0.616	0.125	^**^	0.125	^*^
CXCL8 (IL8)	0.207	^***^	0.180	^***^	0.050	0.283	0.032	0.520	0.068	0.146	0.039	0.429	0.104	^*^	0.101	^*^
CSF1	0.417	^***^	0.401	^***^	0.106	^*^	0.100	^*^	0.376	^***^	0.383	^***^	−0.018	0.698	−0.039	0.437
MMP9	0.149	0.189	0.156	0.187	0.065	0.162	0.057	0.252	0.384	^***^	0.374	^***^	−0.056	0.234	−0.085	0.088
CD141 (THBD)	0.387	^***^	0.367	^***^	0.119	^*^	0.106	^*^	0.510	^***^	0.530	^***^	−0.043	0.358	−0.078	0.117
CCL22	0.062	0.586	0.182	^***^	0.082	0.079	0.079	0.112	0.311	^***^	0.311	^***^	0.022	0.634	0.025	0.618
CD11c (ITGAX)	0.469	^***^	0.432	^***^	0.110	^*^	0.106	^*^	0.322	^***^	0.326	^***^	−0.069	0.142	−0.123	^*^
CXCL12	0.424	^***^	0.425	^***^	0.084	0.074	0.072	0.145	0.491	^***^	0.504	^***^	−0.045	0.334	−0.087	0.079
CCL17	0.200	^***^	0.146	^**^	0.031	0.502	0.005	0.915	0.270	^***^	0.262	^***^	0.017	0.723	0.025	0.618
VISTA (C10ORF54)	−0.002	0.986	0.252	^***^	0.023	0.617	0.004	0.944	0.262	^***^	0.236	^***^	0.021	0.651	0.003	0.958
CD25 (IL2RA)	0.293	^***^	0.263	^***^	0.129	^**^	0.119	^*^	0.232	^***^	0.215	^***^	0.037	0.434	0.037	0.458
HGF	0.350	^***^	0.337	^***^	0.018	0.694	0.003	0.948	0.589	^***^	0.596	^***^	−0.092	^*^	−0.090	0.070
FOXP3	0.131	0.249	0.094	0.428	0.103	^*^	0.096	0.052	0.359	^***^	0.371	^***^	−0.042	0.366	−0.060	0.230

*COAD, colon adenocarcinoma; Cor, R value of Spearman's correlation; None, correlation without adjustment. Purity, correlation adjusted by purity*.

* *p < 0.05*;

** *p < 0.01*;

*** *p < 0.001*.

### Expression of Four Prognosis-Related Genes in Colorectal Cancer

To better understand the potential of these prognosis-related genes as drug targets, we analyzed their expression levels in normal tissues and colorectal cancer tissues using ONCOMINE 4.5 database. The results revealed that mRNA expression levels of these four genes were significantly higher in colorectal cancer tissues than in normal tissues (*p* < 0.01). Although the fold differences of LEP, DLX2, and CLSTN2 were within 2, they ranked within the top 20% based on mRNA expression (Over-expression Gene Rank (LEP): in top 20%; Over-expression Gene Rank (DLX2): in top 11%; Over-expression Gene Rank (CLSTN2): in top 14%; (Supplementary Figures 2A–C). In addition, REG3A expression level was significantly higher in colorectal cancer tissues than in normal colon tissues (Supplementary Figure 2D).

### Prediction of Potential Therapeutic Drugs for COAD

Given the strong association between distant metastasis and the survival of cancer patients, we postulated that drugs affecting the DEGs in metastatic patients may play an antitumor role in cancer treatment. Among the 127 candidate DEGs, 49 upregulated and 27 downregulated genes were selected as input files for the CMap pilot dataset. As a result, four compounds (Mean ≤ −0.40, *p* < 0.05, percent non-null > 50) were considered candidate drugs: ajmaline, a class IA antiarrhythmic drug used diagnostically (not used therapeutically) to elicit ECG changes in patients suspected to have Brugada syndrome; TTNPB, a synthetic retinoid that acts as a selective agonist for retinoic acid receptors (RARs); dydrogesterone, a progestogen drug used to treat menstrual and premenstrual disorders, endometriosis, infertility and other conditions; and dicycloverine, also known as dicyclomine, an anticholinergic drug used for treating irritable bowel syndrome (IBS) (Table 4). Then, the relationships between the four compounds and cancers were investigated using CTD, and the results revealed that ajmaline, TTNPB, and dydrogesterone could target colonic neoplasms (Supplementary Table 5).

**Table 4:** The four most significant small-molecule drugs for COAD after CMap analysis.

**Rank**	**CMap name**	**Mean**	***n***	**Enrichment**	***p-*value**	**Specificity**	**Percent non-null**	**Biological function**
1	Ajmaline	−0.474	3	−0.859	0.00559	0.0142	66	Class 1A antiarrhythmic agent
2	TTNPB	−0.677	2	−0.927	0.01105	0.0133	100	Selective RAR agonist
3	Dydrogesterone	−0.603	4	−0.711	0.01424	0.0063	75	Progestogen
4	Dicycloverine	−0.442	5	−0.631	0.01774	0.0382	60	Anticholinergic drug

*COAD, colon adenocarcinoma; RAR, retinoic acid receptor*.

## Discussion

In this study, we identified four novel distant metastasis-related genes significantly associated with the survival of COAD patients. In brief, we screened 127 DEGs by comparing the gene expression profiles of primary tumors from metastatic patients with those from primary patients. Most previous studies focused on the differentially expressed regulators between tumor tissue and normal tissue (22–24). However, in the present study, we analyzed DEmiRNAs and DEGs between non-distant metastatic and distant metastatic COAD patients. The overlap between distant metastasis-related genes and prognostic factors represents an important group of potential biomarkers and therapeutic targets. Moreover, the expression levels of LEP, DLX2, CLSTN2, and REG3A were significantly higher in tumor tissues than normal tissues. Based on this, we determined four small-molecule drugs (ajmaline, TTNPB, dydrogesterone, and dicycloverine) that potentially target these genes according to the data from CMap. As the CMap results are based on small-molecule perturbations profiled across a large number of cell types (17), we further validated the relationships between the four candidate drugs and colon carcinoma in CTD. Among the four drugs, the results suggested that three drugs, including ajmaline, TTNPB, and dydrogesterone, could treat colon carcinoma.

Notably, compared to four individual prognosis-associated genes, the combined gene signature panel had better discrimination performance in COAD patients. LEP was reported to promote cancer cell migration and invasion (25, 26). DLX2 was indicated to increase the risk of metastasis in prostate cancer patients with a high expression of Ki67 (27). In addition, CLSTN2 was demonstrated to be involved in both the tumorigenesis and pulmonary metastasis of osteosarcoma (28). Interestingly, REG3A was found to promote cell proliferation in gastric and colorectal cancers (29, 30). However, the present study found that REG3A correlates with favorable survival in colorectal cancer, and is down-regulated in primary tumors of patients with distant metastasis (M1) compared to patients without distant metastasis (M0). Consistent with this result, Qiu et al. reported that REG3A was a tumor suppressor in gastric cancer, while REG3A overexpression could inhibit the invasion and proliferation and promote the apoptosis of gastric cancer cells *via* the phosphatidylinositol 3-kinase (PI3K)/Akt-GSK3 signaling pathway axis (31). From this perspective, REG3A might promote tumor cells proliferation, but suppress metastatic cascade indirectly. The understanding of the roles of these four genes in the metastasis of colorectal cancer may be improved by analyzing lager cohorts and performing *in vitro* and *in vivo* experiments.

In general, tumor development could be controlled by cytotoxic innate and adaptive immune cells. However, cancer cells evolve different mechanisms that mimic peripheral immune tolerance to avoid tumoricidal attack. Furthermore, increasing evidence reveals that tumor-infiltrating immune cells could promote the metastatic cascade thus affecting clinical outcome (32, 33). In this study, we found that expression of LEP, DLX2, CLSTN2, and REG3A are correlated with immune infiltrating levels of CD8+ T cells, CD4+ T cells, macrophages, neutrophils, and DCs in COAD, indicating that these four prognosis-associated genes expression levels could also reflect immune status, which partially explains that LEP, DLX2 and CLSTN2 are predictors of poor outcome while REG3A indicates better outcome and lower probability of distant metastasis in COAD.

To develop more treatment options for COAD, we found that three existing drugs may be used for treating COAD. First, ajmaline, an indole alkaloid commonly used for hypertension, was found to inhibit DNA synthesis by arresting cells at the G2 phase and promoting apoptosis in prostate cancer cells (34). As a voltage-gated Na+ channels (VGSCs) blocker, ajmaline preferentially binds to the open state of cardiac Na+ channel protein (Nav1.5) (35). In recent years, VGSCs have been recognized as the invasiveness-associated proteins, and they are exclusively highly expressed in aggressive tumor biopsies and metastatic cancer cells (36–38). Specifically, Nav1.5, a neonatal splice variant of VGSCs, has been found to promote the breast cancer cell invasion *in vitro* and metastasis *in vivo* (39). Thus, VGSCs are a class of promising anti-metastasis drug targets (40), especially Nav1.5, which is a neonatal isoform not commonly expressed in normal adult tissues (41, 42). There are numerous clinically used drugs that target Nav1.5, but some undesirable effects such as local anesthetics, antiarrhythmics, and anticonvulsants restrict their application in treating metastatic cancers (43). More recently, researchers are focusing on exploring the proper dose of old drugs and developing novel tissue-specific drugs to treat metastatic cancers by inhibiting VGSCs. For example, Shilpa et al. designed and synthesized five small molecule compounds to inhibit the invasion of MDA-MB-231 cells (a highly aggressive human breast cancer cell line) through blocking Nav1.5-dependent inward currents (44). Moreover, Hatice et al. reported that low concentrations of naringenin (5 and 10 μM), a natural compound found in citrus fruits and tomatoes, could inhibit MAT-LyLu cells (a highly metastatic prostate cancer cell line) metastasis by blocking VGSCs (45). Also, Nelson et al. proposed that phenytoin, a sodium channel-blocking antiepileptic drug, at a dose equivalent to that used to treat epilepsy (60 mg/kg; daily), could significantly reduce breast tumor metastasis to the liver, lungs, and spleen in orthotopic tumor models (46). Besides, Baptista-Hon et al. showed that VGSCs expressed in colon cancer cells could promote invasion. Apart from this, ropivacaine, an inhibitor of Nav1.5, could inhibit invasion of SW620 cells (a metastatic colon cancer cell line), with a dose of inhibition of 3.8 μM (47). Second, TTNPB is a retinoic acid analog that acts as a selective RAR agonist. It has been reported that an analog of TTNPB, 4-hydroxybenzyl-modified compound (4HBTTNPB), could induce apoptosis in breast cancer cells (48). For dydrogesterone, only one case has been reported. In brief, one patient with recurrent endometrial stromal sarcoma was treated exclusively with dydrogesterone at a daily dose of 10 mg, and the tumor clinically disappeared after 4 years and 3 months (49). To date, no studies have focused on the three drugs in COAD. Therefore, future studies are needed to explore the key molecular targets and therapeutic effects of these three drugs in COAD.

In summary, we identified four novel prognosis-related genes and three potential drugs for COAD patients, which may serve to unravel the complex gene networks of distant metastasis with clinical relevance.

## Data Availability Statement

All datasets generated for this study are included in the article/Supplementary Material.

## Author Contributions

MW participated in its design, analysis, interpretation, and manuscript drafting. WL assisted in making the study design and evaluating the results obtained. X-FY supervised the study, participated in its design, interpretation, analysis, and including drafting. All authors contributed to drafting, reviewing the manuscript, and approved the submission of the final version.

## Conflict of Interest

The authors declare that the research was conducted in the absence of any commercial or financial relationships that could be construed as a potential conflict of interest.

## Supplementary Material

Supplementary Figure 1 Prognostic value of the combined gene signature panel. **(A)** K-M curve based on low-risk group and high-risk group. **(B)** ROC curve (AUC = 0.672). K-M, Kaplan-Meier; OS, overall survival; ROC, operating characteristic curve; AUC, area under the curve.

Supplementary Figure 2 Expression of four selected genes in colorectal cancer (Oncomine database). Box plots derived from mRNA expression data in Oncomine are shown, comparing expression of specific genes in normal and tumor tissues. ^*^*p* < 0.05, ^**^*p* < 0.01, and ^***^*p* < 0.001. **(A)** Comparison of LEP mRNA expression (Skrzypczak Colorectal 2). **(B)** Comparison of DLX2 mRNA expression (TCGA Colorectal). **(C)** Comparison of CLSTN2 mRNA expression (Graudens Colon). **(D)** Comparison of REG3A mRNA expression (TCGA Colorectal).

Supplementary Table 1 Identification of differentially expressed genes (DEGs) in TCGA-COAD patients with or without distant metastasis.

Supplementary Table 2 Shared genes between the targets of the DEmiRNAs and DEGs.

Supplementary Table 3 Gene Ontology (GO) term enrichment for the prognosis-associated genes differentially expressed in distant metastatic COAD.

Supplementary Table 4 The relationship between cancers and prognosis-associated genes differentially expressed in distant metastatic COAD based on CTD.

Supplementary Table 5 Relationship between cancers and three potential drugs for COAD based on CTD.
