# Supplementary figures and images for: Integrated Analysis of Distant Metastasis-Associated Genes and Potential Drugs in Colon Adenocarcinoma
Source: Front Oncol. 2020 Oct 23;10:576615. doi: 10.3389/fonc.2020.576615 (PMC7645237; doi:10.3389/fonc.2020.576615)

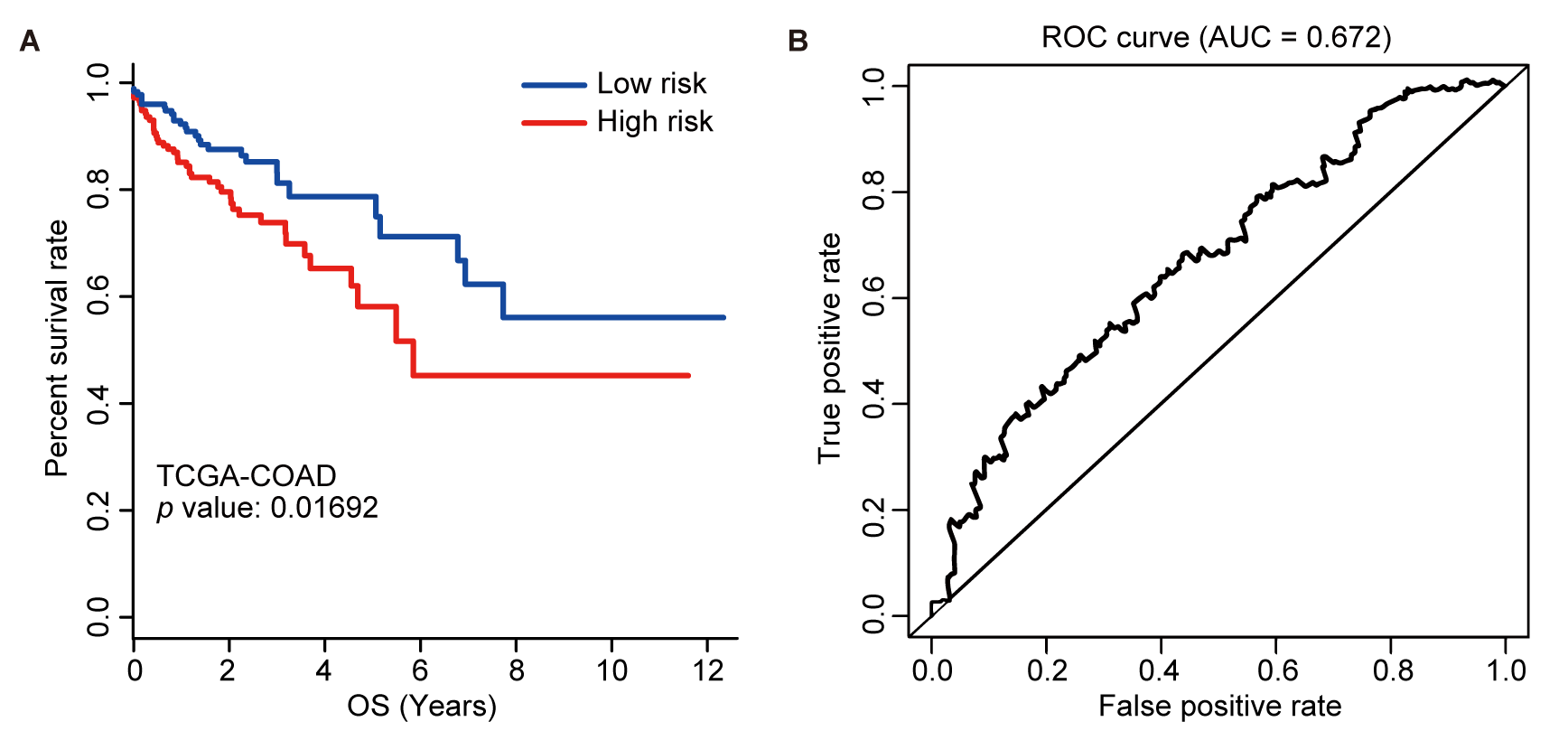

Supplement: Supplementary Figure 1 — Prognostic value of the combined gene signature panel. (A) K-M curve based on low-risk group and high-risk group. (B) ROC curve (AUC = 0.672). K-M, Kaplan-Meier; OS, overall survival; ROC, operating characteristic curve; AUC, area under the curve. [file Image_1.TIF]

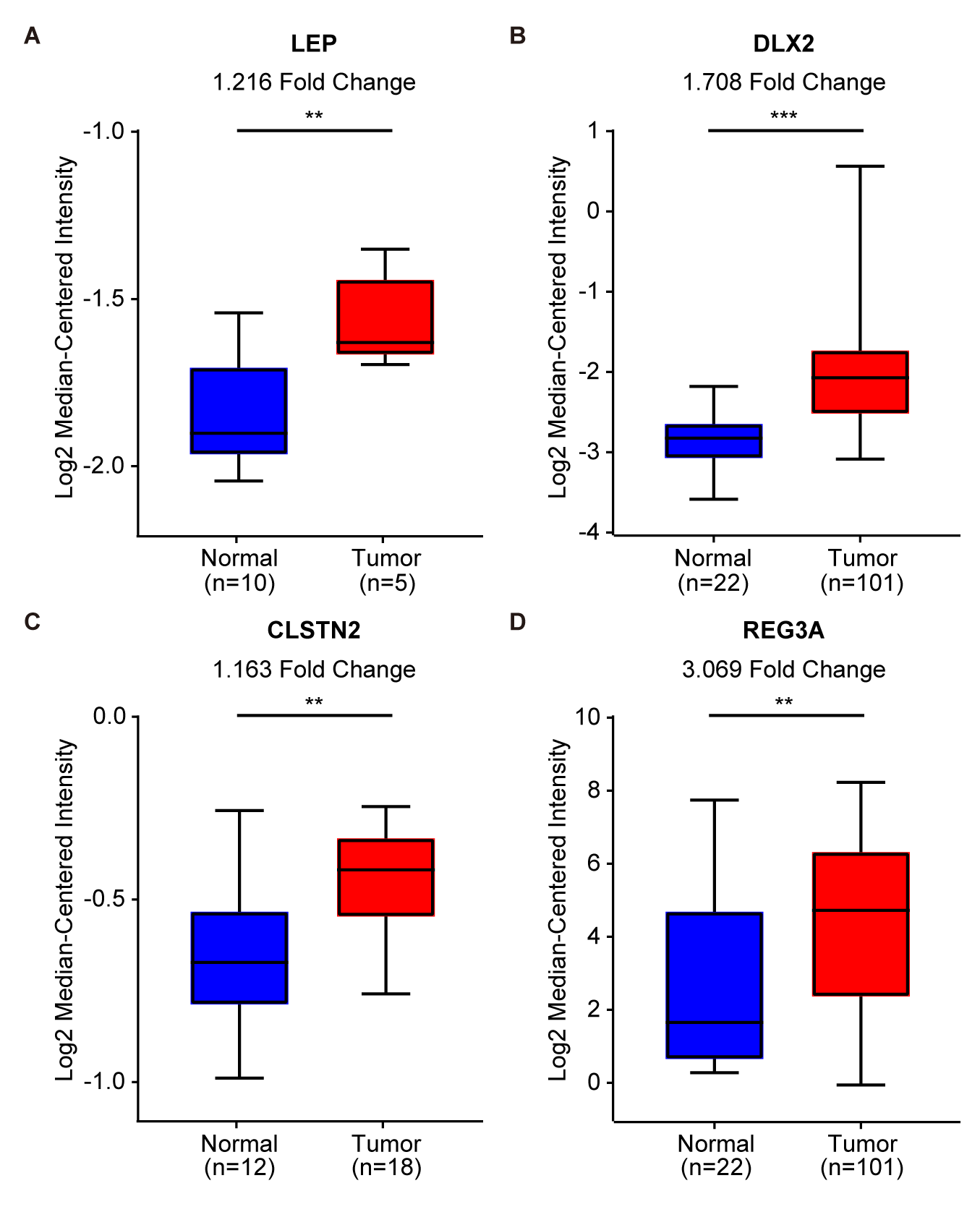

Supplement: Supplementary Figure 2 — Expression of four selected genes in colorectal cancer (Oncomine database). Box plots derived from mRNA expression data in Oncomine are shown, comparing expression of specific genes in normal and tumor tissues. *p < 0.05, **p < 0.01, and ***p < 0.001. (A) Comparison of LEP mRNA expression (Skrzypczak Colorectal 2). (B) Comparison of DLX2 mRNA expression (TCGA Colorectal). (C) Comparison of CLSTN2 mRNA expression (Graudens Colon). (D) Comparison of REG3A mRNA expression (TCGA Colorectal). [file Image_2.TIF]
