# Supplementary material for: Integrated Analysis of Distant Metastasis-Associated Genes and Potential Drugs in Colon Adenocarcinoma
Source: Front Oncol. 2020 Oct 23;10:576615. doi: 10.3389/fonc.2020.576615 (PMC7645237; doi:10.3389/fonc.2020.576615)
Supplement: Supplementary Table 1 — Identification of differentially expressed genes (DEGs) in TCGA-COAD patients with or without distant metastasis. [file Table_1.DOC]

**Table S1** Identification of differentially expressed genes (DEGs) in TCGA-COAD patients with or without distant metastasis.

| **Gene symbol** | **Log2FC** | **LogCPM** | ***p* value** | **FDR** |
| --- | --- | --- | --- | --- |
| LINC00400 | 6.499073 | -3.028484 | 3.12E-39 | 1.90E-35 |
| TFAP2B | 6.118766 | -1.925171 | 6.31E-53 | 1.93E-48 |
| AC107953.2 | 5.293238 | -2.985287 | 3.27E-49 | 4.99E-45 |
| KRTAP4-6 | 5.211014 | -3.431511 | 1.60E-33 | 4.44E-30 |
| UNCX | 5.105072 | -2.90793 | 2.74E-38 | 1.39E-34 |
| PSG1 | 5.092134 | -3.190715 | 2.26E-29 | 4.07E-26 |
| NEUROD4 | 4.973052 | -3.397635 | 2.42E-25 | 2.73E-22 |
| GALP | 4.952574 | -3.459859 | 9.91E-30 | 2.16E-26 |
| PSG7 | 4.742893 | -3.47804 | 1.97E-25 | 2.31E-22 |
| PSG9 | 4.607979 | -3.049142 | 1.14E-30 | 2.68E-27 |
| LHX3 | 4.547202 | -2.446738 | 1.07E-39 | 8.15E-36 |
| DCAF4L2 | 4.419107 | -2.656622 | 2.92E-18 | 1.44E-15 |
| PSG6 | 4.418226 | -3.502097 | 1.04E-19 | 6.60E-17 |
| PPP1R17 | 4.273369 | -3.022893 | 1.84E-29 | 3.50E-26 |
| SBK2 | 4.225678 | -3.308713 | 3.92E-25 | 4.13E-22 |
| CER1 | 4.099231 | -2.191474 | 6.32E-37 | 2.75E-33 |
| FOXR2 | 3.991705 | -3.620357 | 5.82E-17 | 2.57E-14 |
| GPR50 | 3.971337 | -3.468182 | 4.42E-17 | 2.01E-14 |
| PSG11 | 3.963154 | -3.503492 | 2.01E-18 | 1.00E-15 |
| AC132825.1 | 3.922284 | -1.298493 | 3.46E-43 | 3.52E-39 |
| MTND5P32 | 3.901976 | -3.200291 | 4.85E-19 | 2.64E-16 |
| AC066616.2 | 3.879488 | -3.41596 | 1.42E-19 | 8.49E-17 |
| FGF6 | 3.810027 | -3.686821 | 1.34E-19 | 8.17E-17 |
| AC087612.1 | 3.808944 | -2.584352 | 9.38E-26 | 1.14E-22 |
| CALML5 | 3.805707 | -1.984422 | 1.96E-19 | 1.15E-16 |
| TUBA3C | 3.762741 | -3.251761 | 2.14E-21 | 1.67E-18 |
| FER1L6-AS1 | 3.755763 | -3.235478 | 6.06E-22 | 5.14E-19 |
| AC108359.1 | 3.518086 | -3.49755 | 1.70E-21 | 1.37E-18 |
| ARL14EPL | 3.495412 | -3.507331 | 4.12E-22 | 3.59E-19 |
| TBX5-AS1 | 3.439163 | -3.244935 | 3.86E-25 | 4.13E-22 |
| BX276092.7 | 3.409667 | -3.502091 | 1.71E-16 | 7.24E-14 |
| LINC02617 | 3.379965 | -3.501655 | 5.18E-25 | 5.27E-22 |
| CT45A1 | 3.356455 | -3.003819 | 6.06E-10 | 1.13E-07 |
| CHRNA4 | 3.305165 | -2.688135 | 1.76E-15 | 6.64E-13 |
| CDH12 | 3.249742 | -3.146635 | 1.33E-18 | 7.00E-16 |
| GPR1-AS | 3.239148 | -3.581543 | 5.64E-18 | 2.73E-15 |
| XAGE2 | 3.228629 | -1.870318 | 7.11E-12 | 1.78E-09 |
| LINC02582 | 3.206961 | -0.572077 | 5.14E-12 | 1.32E-09 |
| AC064872.1 | 3.198843 | -2.780073 | 8.92E-10 | 1.52E-07 |
| SALL3 | 3.18994 | -2.639801 | 1.40E-12 | 4.07E-10 |
| HRH3 | 3.16435 | -2.512145 | 3.64E-16 | 1.48E-13 |
| NBEAP1 | 3.083596 | -2.791796 | 7.92E-35 | 2.68E-31 |
| LINC02303 | 3.071887 | -3.226962 | 2.07E-13 | 6.63E-11 |
| CYP4F32P | 3.067017 | -3.653591 | 9.95E-16 | 3.94E-13 |
| NFE4 | 3.031014 | -2.819683 | 1.20E-28 | 1.92E-25 |
| MIR548XHG | 3.013492 | -3.58002 | 2.01E-11 | 4.69E-09 |
| RMST | 3.009772 | -3.202333 | 2.54E-17 | 1.18E-14 |
| PSG4 | 3.003317 | -3.410634 | 1.78E-16 | 7.43E-14 |
| SLITRK1 | 2.987268 | -3.432935 | 1.14E-19 | 7.09E-17 |
| SHISA7 | 2.940036 | -2.56448 | 2.99E-36 | 1.14E-32 |
| NCAN | 2.925313 | -2.443535 | 9.30E-28 | 1.42E-24 |
| PAGE2 | 2.921505 | -3.561784 | 6.97E-17 | 3.04E-14 |
| ARHGAP36 | 2.915747 | -2.43236 | 6.64E-21 | 5.06E-18 |
| CACNG7 | 2.901996 | -2.51389 | 1.45E-26 | 2.01E-23 |
| CHST8 | 2.898903 | -2.104696 | 1.47E-29 | 2.99E-26 |
| PNMA5 | 2.88774 | 2.630678 | 2.08E-09 | 3.21E-07 |
| RNF17 | 2.881133 | -2.969074 | 2.63E-22 | 2.36E-19 |
| AC022778.1 | 2.863702 | -3.421394 | 6.21E-10 | 1.14E-07 |
| MAGEC2 | 2.830359 | -2.208173 | 4.87E-09 | 6.81E-07 |
| CHGB | 2.795083 | 3.181318 | 9.10E-21 | 6.61E-18 |
| NEUROD1 | 2.791645 | 0.223904 | 1.89E-12 | 5.29E-10 |
| CBLN4 | 2.787807 | -1.777348 | 1.62E-20 | 1.15E-17 |
| AC079209.2 | 2.78149 | -3.492697 | 2.36E-10 | 4.67E-08 |
| NEUROD2 | 2.765287 | -2.177796 | 3.88E-23 | 3.70E-20 |
| SUMO1P1 | 2.76077 | -3.457877 | 8.64E-17 | 3.71E-14 |
| PHF2P2 | 2.751999 | -2.736777 | 4.44E-15 | 1.63E-12 |
| AC084871.2 | 2.745025 | -2.997297 | 3.40E-19 | 1.92E-16 |
| TGM6 | 2.688707 | -2.991893 | 7.08E-11 | 1.53E-08 |
| UTS2B | 2.683406 | -1.568916 | 7.32E-27 | 1.06E-23 |
| LRRC53 | 2.654641 | -2.872541 | 5.09E-13 | 1.54E-10 |
| ANXA8 | 2.653532 | -1.425336 | 1.02E-13 | 3.45E-11 |
| SDCBPP1 | 2.628093 | -3.268487 | 1.42E-11 | 3.39E-09 |
| SRRM4 | 2.622148 | -2.726196 | 7.04E-20 | 4.67E-17 |
| HIF1A-AS3 | 2.621713 | -1.205026 | 6.48E-32 | 1.65E-28 |
| SLC1A6 | 2.59881 | -3.373062 | 1.46E-15 | 5.62E-13 |
| TEX15 | 2.588654 | -2.958902 | 1.10E-11 | 2.69E-09 |
| LINC02393 | 2.576395 | -3.284364 | 1.36E-10 | 2.83E-08 |
| AC023310.4 | 2.559685 | -3.603631 | 1.74E-18 | 8.84E-16 |
| PRICKLE2-AS3 | 2.551076 | -3.310009 | 1.97E-09 | 3.07E-07 |
| TBR1 | 2.549102 | -2.938915 | 6.25E-11 | 1.38E-08 |
| MMD2 | 2.531892 | -3.630908 | 3.48E-13 | 1.11E-10 |
| PRDX3P4 | 2.527402 | -3.463932 | 1.92E-09 | 3.02E-07 |
| MTATP8P2 | 2.51739 | -0.658043 | 4.92E-26 | 6.26E-23 |
| AC097374.1 | 2.514035 | -3.681071 | 8.40E-14 | 2.91E-11 |
| BRDT | 2.513094 | -3.412861 | 4.06E-19 | 2.25E-16 |
| CDH9 | 2.478965 | -2.767462 | 2.89E-08 | 3.57E-06 |
| SCRT2 | 2.465362 | -3.530279 | 2.15E-10 | 4.31E-08 |
| BAGE2 | 2.456753 | -3.507551 | 1.24E-06 | 9.20E-05 |
| SEZ6 | 2.448505 | -1.197592 | 5.14E-19 | 2.75E-16 |
| COX7B2 | 2.414162 | -3.0595 | 6.80E-07 | 5.51E-05 |
| TCF23 | 2.404593 | -1.819213 | 2.38E-17 | 1.12E-14 |
| DOCK4-AS1 | 2.370641 | -3.193893 | 8.53E-08 | 9.17E-06 |
| WNT7A | 2.369909 | -1.59237 | 3.90E-12 | 1.04E-09 |
| SOHLH2 | 2.352603 | -1.96506 | 1.17E-10 | 2.46E-08 |
| WDR11-AS1 | 2.327138 | -3.016708 | 1.52E-18 | 7.87E-16 |
| LINC00973 | 2.313827 | -2.506929 | 6.66E-10 | 1.20E-07 |
| FAM9A | 2.304736 | -3.208319 | 1.96E-08 | 2.52E-06 |
| AC011632.1 | 2.286715 | -2.66417 | 5.49E-10 | 1.03E-07 |
| MYH16 | 2.28455 | -0.446135 | 2.92E-26 | 3.88E-23 |
| DPYSL5 | 2.254032 | -1.286888 | 2.11E-14 | 7.42E-12 |
| AC079316.2 | 2.253663 | -3.374694 | 2.38E-07 | 2.22E-05 |
| THSD7B | 2.25336 | -1.289021 | 5.65E-16 | 2.27E-13 |
| LINC02476 | 2.25287 | -3.25754 | 7.25E-06 | 0.000397 |
| LEP | 2.243944 | -1.188442 | 2.23E-12 | 6.09E-10 |
| TMEM132D-AS1 | 2.242586 | 0.141481 | 2.81E-05 | 0.001195 |
| COLEC10 | 2.241202 | -1.762328 | 1.11E-34 | 3.38E-31 |
| CT45A10 | 2.237621 | -3.416866 | 1.94E-05 | 0.000897 |
| FBXO40 | 2.227465 | -3.314597 | 1.76E-09 | 2.79E-07 |
| EZHIP | 2.225418 | -2.943292 | 1.69E-06 | 0.000119 |
| AC009951.4 | 2.219657 | -2.00145 | 3.17E-08 | 3.82E-06 |
| AC012494.2 | 2.218599 | -3.571253 | 1.07E-09 | 1.78E-07 |
| AC026333.3 | 2.209072 | -3.405165 | 7.95E-09 | 1.07E-06 |
| LNCAROD | 2.196064 | -1.779843 | 2.22E-06 | 0.000149 |
| MEIS1-AS2 | 2.190977 | -3.44301 | 1.44E-11 | 3.40E-09 |
| EEF1A2 | 2.189362 | 1.711794 | 9.92E-20 | 6.44E-17 |
| CCKBR | 2.168008 | -2.640543 | 8.77E-10 | 1.50E-07 |
| COL2A1 | 2.167727 | 1.046014 | 1.63E-14 | 5.84E-12 |
| MAGEC1 | 2.155032 | -2.16029 | 1.56E-05 | 0.000753 |
| TACR3 | 2.149954 | -3.570453 | 7.44E-12 | 1.85E-09 |
| AC105910.1 | 2.14577 | -3.543167 | 8.73E-10 | 1.50E-07 |
| CHRNA6 | 2.125455 | -2.394429 | 5.40E-20 | 3.66E-17 |
| AP003072.5 | 2.12052 | -3.456421 | 2.44E-09 | 3.64E-07 |
| PHF21B | 2.115882 | -3.008835 | 1.43E-13 | 4.68E-11 |
| AC011595.1 | 2.114839 | -3.530059 | 2.67E-07 | 2.43E-05 |
| ERICH3 | 2.103706 | -2.561307 | 2.17E-11 | 4.94E-09 |
| CAMK2B | 2.10296 | -0.64047 | 1.46E-21 | 1.20E-18 |
| ATP2B3 | 2.097362 | -2.234877 | 6.44E-11 | 1.41E-08 |
| MIR7-3HG | 2.092483 | -2.847518 | 8.97E-10 | 1.52E-07 |
| LINC02753 | 2.091757 | -3.393012 | 5.06E-08 | 5.85E-06 |
| CST6 | 2.089708 | 0.124918 | 3.08E-16 | 1.27E-13 |
| LINC00648 | 2.088081 | -2.791677 | 2.69E-07 | 2.43E-05 |
| DMP1 | 2.08197 | -3.39146 | 4.19E-13 | 1.29E-10 |
| NKX6-3 | 2.08129 | 0.446774 | 2.05E-06 | 0.000141 |
| VCAN-AS1 | 2.074835 | -3.156086 | 2.46E-07 | 2.29E-05 |
| CRHR1 | 2.069949 | -3.44811 | 7.18E-10 | 1.28E-07 |
| SLC38A8 | 2.067525 | -2.704657 | 6.08E-10 | 1.13E-07 |
| LINC02078 | 2.06226 | -3.140613 | 1.24E-13 | 4.12E-11 |
| MAGEB1 | 2.047198 | -3.679848 | 7.56E-05 | 0.002679 |
| SP3P | 2.045627 | -3.196145 | 1.56E-10 | 3.20E-08 |
| ADAMTS20 | 2.043474 | -3.04253 | 5.98E-06 | 0.000339 |
| AC106799.3 | 2.041452 | -3.449497 | 4.05E-06 | 0.000245 |
| HMGB1P37 | 2.035573 | -2.21315 | 7.72E-24 | 7.60E-21 |
| AC022973.4 | 2.035283 | -2.789719 | 2.99E-08 | 3.68E-06 |
| UGT3A1 | 2.030233 | -2.782223 | 3.10E-05 | 0.001301 |
| ANKRD34C | 2.029331 | -3.498656 | 6.70E-10 | 1.20E-07 |
| RPL23AP30 | 2.025103 | -3.396591 | 1.34E-08 | 1.76E-06 |
| AC100800.1 | 2.007236 | -2.990643 | 7.27E-05 | 0.002606 |
| C6orf15 | 2.005267 | 1.671815 | 7.62E-09 | 1.03E-06 |
| AF178030.1 | 1.999197 | -3.613473 | 4.90E-05 | 0.00188 |
| KIF1A | 1.998024 | 1.431092 | 1.30E-12 | 3.86E-10 |
| INSL5 | 1.997731 | 1.86135 | 4.04E-05 | 0.001604 |
| LINC01896 | 1.986098 | -3.518289 | 9.85E-05 | 0.003292 |
| TDRD12 | 1.985345 | -1.427366 | 8.21E-21 | 6.11E-18 |
| AL583808.1 | 1.976275 | -3.394187 | 1.88E-06 | 0.00013 |
| HCN4 | 1.972227 | -2.507956 | 1.54E-15 | 5.88E-13 |
| DSCR8 | 1.971949 | -2.689145 | 0.00011 | 0.003583 |
| CCDC144A | 1.963523 | -1.610872 | 1.49E-10 | 3.06E-08 |
| C9orf170 | 1.952739 | -3.502232 | 2.05E-11 | 4.74E-09 |
| GOLGA6L7 | 1.945034 | -3.588715 | 6.31E-07 | 5.21E-05 |
| ACTL6B | 1.939824 | -2.989338 | 2.34E-10 | 4.67E-08 |
| AL138828.1 | 1.939107 | -2.901569 | 8.52E-10 | 1.49E-07 |
| AC008957.3 | 1.939046 | -2.765531 | 6.51E-11 | 1.42E-08 |
| DACH2 | 1.931115 | -2.686871 | 9.57E-12 | 2.36E-09 |
| AC092490.1 | 1.924882 | -1.823117 | 2.09E-15 | 7.76E-13 |
| DPYD-AS1 | 1.9153 | -3.597921 | 1.45E-07 | 1.43E-05 |
| AC106820.4 | 1.911569 | -1.419632 | 9.20E-29 | 1.56E-25 |
| AC068870.3 | 1.899958 | -3.473869 | 6.31E-08 | 7.08E-06 |
| MTND4P14 | 1.899801 | -3.560755 | 5.66E-07 | 4.72E-05 |
| LINC01419 | 1.894705 | -1.513444 | 0.000194 | 0.005474 |
| EEF1A1P26 | 1.894534 | -3.36706 | 1.15E-11 | 2.80E-09 |
| LHX1-DT | 1.889669 | -3.439786 | 4.31E-06 | 0.000259 |
| AC024940.2 | 1.875374 | -0.161086 | 5.00E-23 | 4.62E-20 |
| AC009948.4 | 1.875209 | -2.579435 | 4.33E-05 | 0.0017 |
| RIMS4 | 1.866773 | -0.985652 | 3.05E-09 | 4.42E-07 |
| MAGEA10 | 1.859467 | -1.270984 | 0.000292 | 0.007531 |
| SPOCK3 | 1.850946 | -1.837039 | 1.32E-07 | 1.33E-05 |
| AL357568.2 | 1.848819 | -3.624879 | 9.20E-05 | 0.003125 |
| PSCA | 1.844897 | 2.394633 | 6.10E-08 | 6.92E-06 |
| AC011503.1 | 1.837595 | -3.382503 | 2.80E-11 | 6.32E-09 |
| AL133445.2 | 1.832162 | -2.857255 | 1.20E-10 | 2.51E-08 |
| AC010627.1 | 1.830575 | -1.778528 | 3.91E-13 | 1.22E-10 |
| AL589655.1 | 1.817209 | -3.273825 | 0.000976 | 0.018559 |
| COL11A2 | 1.816877 | 0.759723 | 5.22E-20 | 3.62E-17 |
| AL159159.1 | 1.813404 | -3.443427 | 0.000184 | 0.005282 |
| HTR1DP1 | 1.808895 | -3.560686 | 2.59E-06 | 0.00017 |
| LINC00355 | 1.807133 | -2.143601 | 4.31E-05 | 0.001698 |
| LINC02864 | 1.806152 | -0.272362 | 0.000112 | 0.003634 |
| AC087893.3 | 1.801646 | -3.345251 | 1.09E-07 | 1.12E-05 |
| AL731563.2 | 1.798754 | -3.58007 | 1.36E-07 | 1.36E-05 |
| AC036222.3 | 1.794269 | -3.420398 | 5.65E-09 | 7.77E-07 |
| AC025419.1 | 1.788027 | -3.219315 | 1.88E-12 | 5.29E-10 |
| AC068389.3 | 1.784529 | -3.415414 | 8.21E-05 | 0.00287 |
| CD200R1L-AS1 | 1.769229 | -3.072782 | 0.000269 | 0.007039 |
| SLC13A5 | 1.76563 | -1.263765 | 1.32E-12 | 3.86E-10 |
| ANXA8L1 | 1.764363 | -1.455565 | 2.06E-12 | 5.71E-10 |
| PAX4 | 1.752037 | -1.012889 | 1.96E-06 | 0.000135 |
| DKK1 | 1.747068 | 1.617506 | 1.72E-08 | 2.24E-06 |
| A2ML1 | 1.740901 | 0.209657 | 2.35E-06 | 0.000156 |
| PRB1 | 1.739404 | -3.359296 | 5.78E-05 | 0.002164 |
| ZNF560 | 1.738258 | -3.531855 | 3.05E-09 | 4.42E-07 |
| CLCNKB | 1.733464 | -2.262229 | 4.40E-12 | 1.15E-09 |
| FOXC2-AS1 | 1.728511 | -3.532832 | 3.76E-06 | 0.000231 |
| RLIMP1 | 1.726382 | -1.871742 | 4.77E-05 | 0.001845 |
| MTCYBP3 | 1.724767 | -3.358576 | 1.98E-05 | 0.000915 |
| WIF1 | 1.720668 | 2.395271 | 1.15E-05 | 0.000581 |
| DSG1 | 1.712425 | -0.587351 | 2.83E-19 | 1.63E-16 |
| AP003696.1 | 1.711984 | -2.843839 | 0.00017 | 0.004986 |
| HMGB1P51 | 1.711088 | -3.567023 | 3.08E-05 | 0.001296 |
| AC044840.1 | 1.708759 | -3.345635 | 1.24E-05 | 0.000621 |
| AC012499.1 | 1.7078 | -3.055945 | 1.66E-08 | 2.16E-06 |
| SCN1A | 1.706056 | -3.007447 | 1.95E-07 | 1.86E-05 |
| HSD3BP4 | 1.701163 | -3.443359 | 4.56E-09 | 6.48E-07 |
| GPR87 | 1.700599 | -1.754881 | 9.92E-05 | 0.003302 |
| AL023584.2 | 1.700558 | -3.622377 | 8.59E-07 | 6.69E-05 |
| KRT84 | 1.697444 | -3.144283 | 3.47E-06 | 0.000217 |
| AC007336.3 | 1.697112 | -2.754572 | 1.64E-09 | 2.64E-07 |
| CCNYL2 | 1.695527 | -3.509497 | 6.63E-10 | 1.20E-07 |
| NAALADL2-AS2 | 1.691328 | -3.388992 | 1.01E-06 | 7.71E-05 |
| FEV | 1.689722 | -1.040927 | 4.99E-07 | 4.26E-05 |
| FAM216B | 1.688853 | -2.10609 | 5.50E-10 | 1.03E-07 |
| AC119673.1 | 1.687706 | -3.152731 | 1.03E-06 | 7.86E-05 |
| KIRREL2 | 1.685654 | -1.678514 | 1.44E-12 | 4.15E-10 |
| ASNSP1 | 1.684222 | -3.376293 | 7.79E-06 | 0.00042 |
| LINC01087 | 1.680842 | -3.476541 | 1.74E-05 | 0.000826 |
| AC104763.2 | 1.680364 | -3.41467 | 0.000304 | 0.007754 |
| SPINT4 | 1.679435 | -3.39543 | 0.000353 | 0.008689 |
| CA10 | 1.677514 | -2.010682 | 8.70E-10 | 1.50E-07 |
| CNMD | 1.676662 | -3.087389 | 1.04E-07 | 1.08E-05 |
| AC104041.1 | 1.665666 | -3.593539 | 3.59E-06 | 0.000224 |
| BTBD17 | 1.663358 | -3.016849 | 6.55E-08 | 7.24E-06 |
| ACTBL2 | 1.65562 | -2.542284 | 5.42E-07 | 4.57E-05 |
| ERICH3-AS1 | 1.654964 | -3.561244 | 4.08E-05 | 0.001619 |
| SNRPEP10 | 1.649257 | -3.520359 | 0.000267 | 0.007026 |
| AL139412.1 | 1.644682 | -3.520844 | 6.83E-05 | 0.002477 |
| AC023813.2 | 1.643218 | -3.292742 | 0.00042 | 0.00994 |
| SIDT1-AS1 | 1.642651 | -3.215752 | 5.12E-10 | 9.77E-08 |
| KLF2P4 | 1.640803 | -3.634878 | 0.000259 | 0.006836 |
| AC025280.1 | 1.640716 | -3.592297 | 3.66E-06 | 0.000227 |
| LINC02241 | 1.637859 | -3.389866 | 0.001347 | 0.023274 |
| LINC01239 | 1.63676 | -2.357013 | 8.35E-07 | 6.56E-05 |
| THRB-IT1 | 1.636061 | -3.485132 | 6.38E-06 | 0.000356 |
| BMS1P22 | 1.632846 | -3.514593 | 5.52E-07 | 4.63E-05 |
| LINP1 | 1.631158 | -3.440311 | 0.000796 | 0.016046 |
| RN7SKP163 | 1.627093 | -3.546266 | 9.53E-05 | 0.00321 |
| AL157402.2 | 1.625161 | -3.43021 | 0.001372 | 0.023596 |
| AC087286.1 | 1.62203 | -3.243618 | 0.000919 | 0.017774 |
| DPY19L2P4 | 1.616832 | -3.494837 | 1.10E-07 | 1.12E-05 |
| SYT4 | 1.616113 | -1.054016 | 1.31E-06 | 9.67E-05 |
| RGS21 | 1.615799 | -3.592628 | 7.44E-05 | 0.00265 |
| AC022537.1 | 1.614632 | -3.397896 | 0.002952 | 0.040188 |
| GCG | 1.611274 | 2.785154 | 0.000177 | 0.005161 |
| AC136475.9 | 1.608179 | -2.128132 | 2.98E-11 | 6.70E-09 |
| UPB1 | 1.603657 | -1.403737 | 2.21E-12 | 6.08E-10 |
| SLC14A1 | 1.602282 | 3.224634 | 8.06E-08 | 8.76E-06 |
| LINC02042 | 1.601081 | -2.489203 | 0.001158 | 0.021005 |
| RPRM | 1.595608 | -0.542684 | 4.65E-10 | 8.98E-08 |
| MTND5P2 | 1.592376 | -3.233315 | 0.000101 | 0.003363 |
| CGA | 1.591411 | -3.127088 | 7.59E-07 | 6.03E-05 |
| CD200R1L | 1.590874 | -2.504409 | 3.42E-05 | 0.001405 |
| PTPRJ-AS1 | 1.587806 | -3.430484 | 2.51E-05 | 0.001087 |
| CNTNAP4 | 1.584665 | -3.20278 | 3.80E-05 | 0.001526 |
| IGLON5 | 1.583832 | -0.863131 | 1.10E-15 | 4.31E-13 |
| LHX1 | 1.582 | -2.754999 | 2.73E-05 | 0.001169 |
| PEX5L | 1.581953 | -2.43792 | 2.33E-12 | 6.30E-10 |
| IGFL2-AS1 | 1.581554 | 0.174902 | 1.53E-06 | 0.000109 |
| FUT9 | 1.581428 | -2.970918 | 0.000235 | 0.006331 |
| ZDHHC22 | 1.581133 | -2.617561 | 7.94E-06 | 0.000425 |
| ERVMER61-1 | 1.567034 | -1.15567 | 0.000646 | 0.013785 |
| BRWD1-IT1 | 1.559143 | -3.616179 | 0.000243 | 0.006501 |
| LINC02188 | 1.55374 | -1.644999 | 2.94E-06 | 0.000189 |
| AL035078.1 | 1.548464 | -3.494402 | 1.88E-07 | 1.81E-05 |
| AL138713.1 | 1.548025 | -3.06458 | 0.003225 | 0.042623 |
| AC105460.2 | 1.546928 | -2.631475 | 0.000774 | 0.01573 |
| FAM204CP | 1.546438 | -3.40276 | 0.001841 | 0.029229 |
| SILC1 | 1.546048 | -3.511988 | 8.36E-07 | 6.56E-05 |
| AL359976.1 | 1.545063 | -3.037784 | 5.21E-05 | 0.001979 |
| ITGA2B | 1.543089 | -0.89591 | 5.50E-17 | 2.47E-14 |
| GDAP1L1 | 1.54238 | -1.713364 | 3.21E-11 | 7.14E-09 |
| BHLHA9 | 1.54173 | -2.182291 | 1.86E-05 | 0.000873 |
| CYP7A1 | 1.540349 | -3.214076 | 2.07E-05 | 0.00095 |
| KRTAP13-2 | 1.539348 | -1.750864 | 0.001024 | 0.019203 |
| MRTFA-AS1 | 1.539108 | -2.210717 | 1.54E-07 | 1.50E-05 |
| SLC9C1 | 1.535354 | -0.758696 | 1.84E-14 | 6.52E-12 |
| MUC16 | 1.533835 | 1.308587 | 1.00E-05 | 0.000517 |
| AC020661.2 | 1.533708 | -3.624567 | 0.00047 | 0.01086 |
| AC073316.1 | 1.533067 | -3.182113 | 7.71E-06 | 0.000417 |
| AP004289.2 | 1.532233 | -3.541828 | 0.002097 | 0.031841 |
| DUX4L27 | 1.528053 | -3.559302 | 8.80E-08 | 9.39E-06 |
| RSU1P3 | 1.526601 | -3.484677 | 0.001451 | 0.02461 |
| C1QL4 | 1.52288 | -2.699691 | 2.19E-09 | 3.31E-07 |
| KIF26B-AS1 | 1.522844 | -3.148438 | 4.72E-06 | 0.000278 |
| QRFPR | 1.52276 | -1.834237 | 3.81E-05 | 0.001526 |
| VGLL1 | 1.518872 | -1.189175 | 7.97E-05 | 0.002806 |
| SCN5A | 1.516845 | 0.045763 | 2.03E-13 | 6.58E-11 |
| LINC01929 | 1.5162 | -1.452896 | 1.48E-09 | 2.41E-07 |
| LINC01414 | 1.513456 | -3.469612 | 0.000203 | 0.005651 |
| CLVS2 | 1.512672 | -2.415795 | 9.56E-07 | 7.36E-05 |
| IGFL1P1 | 1.512359 | -2.29251 | 5.40E-06 | 0.000312 |
| DYDC2 | 1.510189 | -2.201918 | 7.39E-06 | 0.000403 |
| AQP4 | 1.509924 | -3.006 | 2.12E-06 | 0.000144 |
| LUCAT1 | 1.508134 | -0.724475 | 2.10E-11 | 4.83E-09 |
| AC108472.1 | 1.507755 | -3.415112 | 1.72E-07 | 1.68E-05 |
| AC112493.1 | 1.504934 | -3.584386 | 0.000147 | 0.00447 |
| RAD17P2 | 1.496534 | -2.832633 | 0.002873 | 0.039481 |
| AL136164.3 | 1.494015 | -3.232768 | 0.00039 | 0.009371 |
| UNC80 | 1.491989 | -2.296797 | 7.51E-09 | 1.02E-06 |
| AC020928.1 | 1.490437 | -2.823567 | 4.08E-12 | 1.07E-09 |
| AC002064.3 | 1.489534 | -2.862818 | 8.19E-08 | 8.86E-06 |
| CSAG2 | 1.488537 | -3.321921 | 0.002171 | 0.032502 |
| CACNG1 | 1.488101 | -3.483293 | 4.81E-05 | 0.001851 |
| AP001011.1 | 1.48728 | -3.505005 | 2.16E-06 | 0.000145 |
| CUX2 | 1.486854 | -2.405496 | 1.02E-09 | 1.71E-07 |
| Z98752.2 | 1.484304 | -3.476141 | 8.42E-07 | 6.59E-05 |
| AC011352.3 | 1.482875 | -2.938443 | 1.63E-06 | 0.000116 |
| SAGE1 | 1.482541 | -2.826966 | 0.001991 | 0.030804 |
| AC006270.1 | 1.482265 | -3.421385 | 0.000159 | 0.004711 |
| SLC9C2 | 1.479714 | -3.396628 | 9.29E-06 | 0.000485 |
| LINC01322 | 1.472414 | -2.885684 | 3.18E-05 | 0.001329 |
| AC022905.1 | 1.47047 | -3.381678 | 9.68E-06 | 0.000502 |
| AC123595.1 | 1.469823 | -3.015583 | 1.37E-09 | 2.24E-07 |
| AC015712.6 | 1.467054 | -1.996149 | 1.69E-12 | 4.82E-10 |
| CLDN10-AS1 | 1.466144 | -1.539309 | 2.30E-06 | 0.000153 |
| POU3F3 | 1.463994 | -1.638615 | 1.10E-06 | 8.30E-05 |
| CLDN19 | 1.458175 | -3.104563 | 9.24E-06 | 0.000483 |
| SLC6A2 | 1.457166 | -2.113799 | 0.000298 | 0.007655 |
| PCDH11Y | 1.454155 | -3.565172 | 0.00014 | 0.004321 |
| AC006460.1 | 1.452801 | -2.73924 | 8.09E-07 | 6.40E-05 |
| SLC6A15 | 1.452338 | -1.211304 | 2.19E-05 | 0.000992 |
| SLC7A14 | 1.45022 | -1.266781 | 1.94E-05 | 0.000899 |
| FOXG1 | 1.450155 | -1.454763 | 0.000652 | 0.013887 |
| GFY | 1.44646 | -3.151504 | 4.29E-05 | 0.001692 |
| MFSD1P1 | 1.445223 | -2.899667 | 2.83E-05 | 0.001201 |
| AC026894.3 | 1.44305 | -3.30642 | 0.000364 | 0.008906 |
| TEX41 | 1.440023 | -1.675208 | 2.15E-09 | 3.30E-07 |
| GK-IT1 | 1.437844 | -2.682495 | 2.21E-08 | 2.80E-06 |
| INSRR | 1.436245 | -2.469606 | 3.50E-09 | 4.99E-07 |
| PCSK2 | 1.435121 | -0.254393 | 0.00014 | 0.004321 |
| CXCL5 | 1.434597 | 5.153618 | 4.69E-06 | 0.000277 |
| LINC02327 | 1.431172 | -3.456054 | 0.003314 | 0.043424 |
| AC107419.1 | 1.431139 | -3.165147 | 0.000275 | 0.007177 |
| FAR2P4 | 1.428291 | -2.504671 | 0.002306 | 0.033865 |
| NECAB2 | 1.427407 | -2.130606 | 1.82E-11 | 4.28E-09 |
| AC079866.2 | 1.427342 | -3.463574 | 0.000526 | 0.011769 |
| AC018682.1 | 1.427198 | -1.903951 | 9.10E-13 | 2.72E-10 |
| LHX9 | 1.425917 | -2.996861 | 4.62E-05 | 0.001795 |
| LINC00942 | 1.424928 | -1.432028 | 6.29E-12 | 1.59E-09 |
| AL139147.1 | 1.424194 | -3.12829 | 2.07E-06 | 0.000142 |
| AL136964.1 | 1.422693 | -3.163841 | 5.52E-06 | 0.000317 |
| PDSS1P1 | 1.421384 | -3.249749 | 2.21E-05 | 0.000998 |
| SLC25A15P3 | 1.421164 | -3.628288 | 5.72E-05 | 0.002149 |
| SCG3 | 1.417682 | 0.285105 | 2.27E-06 | 0.000151 |
| ADGRA1 | 1.417508 | -3.342298 | 9.27E-05 | 0.003148 |
| ADGRD1-AS1 | 1.411828 | -2.984693 | 7.99E-06 | 0.000427 |
| AC022540.1 | 1.411297 | -3.128295 | 8.97E-05 | 0.003081 |
| AC131182.1 | 1.408016 | -2.900976 | 0.001081 | 0.020027 |
| AC011352.1 | 1.407025 | -3.430612 | 1.43E-05 | 0.000704 |
| FABP4 | 1.403942 | 2.079198 | 4.23E-06 | 0.000255 |
| AF127577.6 | 1.403789 | -2.207698 | 8.23E-05 | 0.002874 |
| SCGB3A2 | 1.402177 | -3.394673 | 1.66E-05 | 0.000795 |
| FER1L6-AS2 | 1.400833 | -3.275593 | 2.12E-05 | 0.000971 |
| HOTAIR | 1.399796 | -0.550915 | 0.000232 | 0.006287 |
| AC022893.2 | 1.399553 | -3.014378 | 2.64E-07 | 2.43E-05 |
| EN1 | 1.397634 | -2.517147 | 3.46E-05 | 0.001419 |
| LINC01980 | 1.397084 | -2.025968 | 0.003849 | 0.048045 |
| AL445647.1 | 1.396648 | -2.446696 | 0.000454 | 0.010533 |
| PLD5 | 1.395698 | -3.012096 | 7.18E-06 | 0.000395 |
| PTPRQ | 1.39569 | -3.606561 | 7.41E-05 | 0.002642 |
| AC013410.1 | 1.395645 | -3.323502 | 0.00289 | 0.039636 |
| AC007785.1 | 1.395431 | -3.068828 | 1.45E-05 | 0.000708 |
| RNU6-107P | 1.393294 | -3.56873 | 0.001608 | 0.026527 |
| RPL23AP23 | 1.389967 | -3.473752 | 0.001101 | 0.020284 |
| NKX2-2 | 1.388712 | -1.494589 | 2.34E-05 | 0.001039 |
| PCDHA9 | 1.387423 | -3.465232 | 2.35E-05 | 0.00104 |
| MGAT5B | 1.387076 | -1.66069 | 1.16E-11 | 2.80E-09 |
| LINC01139 | 1.38581 | -2.308092 | 0.000184 | 0.005282 |
| LINC02525 | 1.385129 | -2.233696 | 7.01E-05 | 0.002528 |
| AL159972.1 | 1.383808 | -3.55309 | 0.000127 | 0.004019 |
| IL1RAPL2 | 1.383201 | -3.307805 | 0.000227 | 0.006155 |
| AL355916.1 | 1.381036 | -1.863 | 4.77E-13 | 1.46E-10 |
| GTSF1 | 1.378705 | 0.291656 | 5.03E-06 | 0.000294 |
| LINC00632 | 1.378674 | -2.962689 | 1.35E-09 | 2.22E-07 |
| CLSTN2 | 1.378639 | 2.256227 | 2.96E-09 | 4.36E-07 |
| SIGLEC15 | 1.378025 | 1.516594 | 6.16E-10 | 1.13E-07 |
| AP001885.1 | 1.376471 | -2.669963 | 0.000164 | 0.004843 |
| AC002073.1 | 1.375035 | -3.593684 | 1.66E-05 | 0.000792 |
| INHBA-AS1 | 1.372273 | -2.314491 | 8.44E-11 | 1.80E-08 |
| UPK2 | 1.372061 | -0.599473 | 5.60E-05 | 0.002109 |
| AC092964.1 | 1.370406 | -1.901079 | 2.36E-06 | 0.000156 |
| IL20RB | 1.368772 | 0.714427 | 5.40E-15 | 1.96E-12 |
| KL | 1.368739 | 0.60907 | 1.20E-13 | 4.03E-11 |
| TMEM196 | 1.367348 | -3.624514 | 1.26E-05 | 0.000628 |
| GREB1L | 1.367166 | 0.193769 | 1.23E-06 | 9.12E-05 |
| LINC01429 | 1.366649 | -3.137182 | 7.97E-07 | 6.32E-05 |
| HTR3C | 1.366501 | -1.919829 | 0.000196 | 0.005506 |
| P2RX2 | 1.366271 | -2.105831 | 6.16E-06 | 0.000348 |
| AC018797.2 | 1.366193 | -2.879911 | 7.89E-05 | 0.002784 |
| AL359955.1 | 1.364917 | -2.553863 | 0.000205 | 0.005715 |
| GPR26 | 1.364804 | -3.438244 | 8.08E-05 | 0.002834 |
| C12orf40 | 1.362714 | -3.488306 | 0.000122 | 0.003896 |
| ACTN2 | 1.361446 | -2.451064 | 1.70E-09 | 2.70E-07 |
| AC007750.1 | 1.358646 | -1.87086 | 4.71E-10 | 9.05E-08 |
| OFCC1 | 1.356888 | -2.43442 | 1.46E-05 | 0.000711 |
| MTCO1P12 | 1.353491 | 7.001197 | 7.67E-10 | 1.35E-07 |
| LINC01606 | 1.353424 | -3.611561 | 0.000441 | 0.010312 |
| WIPF3 | 1.351181 | 1.541955 | 8.38E-10 | 1.47E-07 |
| ATCAY | 1.349305 | -1.551803 | 1.69E-06 | 0.000119 |
| DLX2 | 1.347923 | -1.573427 | 6.69E-07 | 5.46E-05 |
| LINC02830 | 1.345572 | -3.020687 | 0.002114 | 0.031979 |
| AC093895.2 | 1.343587 | -2.530341 | 3.79E-05 | 0.001526 |
| AC069431.2 | 1.342891 | -3.054743 | 0.003356 | 0.043885 |
| AL117329.1 | 1.341673 | -2.996482 | 0.003267 | 0.043007 |
| NTN3 | 1.341655 | -2.941232 | 6.17E-08 | 6.95E-06 |
| MKRN3 | 1.340298 | -0.569867 | 0.000192 | 0.005435 |
| AL033397.1 | 1.336785 | -1.23158 | 3.88E-05 | 0.001551 |
| C14orf39 | 1.335579 | -3.417711 | 2.67E-05 | 0.00115 |
| AC027514.1 | 1.333741 | -2.792178 | 2.02E-05 | 0.000929 |
| SLC10A4 | 1.332666 | -1.435757 | 3.22E-09 | 4.61E-07 |
| AC104809.2 | 1.331957 | -0.630179 | 3.25E-05 | 0.001351 |
| LINC00545 | 1.3306 | -3.031137 | 2.30E-05 | 0.001028 |
| AC084871.1 | 1.328915 | -2.946188 | 1.19E-07 | 1.22E-05 |
| SND1-IT1 | 1.328333 | -1.996625 | 0.000876 | 0.017127 |
| MTCO1P11 | 1.327814 | -2.909832 | 1.37E-07 | 1.36E-05 |
| AC015871.4 | 1.327725 | -3.421513 | 0.000335 | 0.008361 |
| FAM133A | 1.324325 | -2.926857 | 1.74E-05 | 0.000826 |
| FSTL5 | 1.322812 | -2.177271 | 9.42E-05 | 0.003186 |
| CIDEA | 1.321812 | -2.200611 | 0.00252 | 0.035904 |
| L1CAM | 1.321191 | 3.946736 | 7.95E-08 | 8.67E-06 |
| GABRG3 | 1.320799 | -2.555553 | 0.001467 | 0.024799 |
| RN7SKP299 | 1.320606 | -3.364593 | 0.00194 | 0.030304 |
| AC006305.2 | 1.317742 | -3.292159 | 2.65E-07 | 2.43E-05 |
| FCF1P5 | 1.317622 | -3.254991 | 0.00011 | 0.003584 |
| LINC01608 | 1.31542 | -3.647183 | 0.000677 | 0.014278 |
| MMP13 | 1.314627 | 1.096912 | 4.03E-06 | 0.000245 |
| VWA5B2 | 1.313845 | 0.568595 | 9.45E-08 | 9.95E-06 |
| MIR181A1HG | 1.313513 | -2.929594 | 0.000374 | 0.009069 |
| AL049820.1 | 1.313201 | -3.620976 | 0.00342 | 0.04441 |
| LINC02584 | 1.311582 | -3.4931 | 8.35E-06 | 0.000442 |
| AL590233.1 | 1.311505 | -3.588242 | 3.00E-05 | 0.001268 |
| ITCH-IT1 | 1.310828 | -3.311457 | 0.000745 | 0.015254 |
| DLX1 | 1.309571 | -1.124836 | 5.05E-06 | 0.000294 |
| TRARG1 | 1.309432 | -0.643566 | 0.001812 | 0.028979 |
| AC092881.1 | 1.308425 | -1.197057 | 0.000118 | 0.003815 |
| AMER3 | 1.307771 | -1.415251 | 0.000125 | 0.003964 |
| AP000487.2 | 1.306997 | -3.48047 | 6.68E-06 | 0.00037 |
| AL049830.4 | 1.305937 | -3.374868 | 0.002098 | 0.031841 |
| LMX1A | 1.305801 | -1.774157 | 0.00015 | 0.004521 |
| KCNH7 | 1.305783 | -2.782141 | 1.16E-10 | 2.45E-08 |
| CSMD3 | 1.304852 | -2.330861 | 3.34E-05 | 0.001378 |
| AC020913.3 | 1.301337 | -3.435038 | 1.48E-06 | 0.000106 |
| ITGB5-AS1 | 1.299536 | -3.50196 | 0.000128 | 0.004025 |
| WNT16 | 1.298685 | -1.783102 | 1.75E-07 | 1.70E-05 |
| SLC6A10P | 1.297423 | -3.428966 | 0.000385 | 0.009267 |
| AL591501.1 | 1.293942 | -3.376367 | 0.000662 | 0.014065 |
| Z99127.1 | 1.29311 | -3.010703 | 0.001122 | 0.020524 |
| AC003986.2 | 1.292505 | -3.481525 | 6.26E-06 | 0.000352 |
| AC110769.3 | 1.29196 | -3.19666 | 0.003048 | 0.041157 |
| PCDHGB7 | 1.290551 | 1.384359 | 3.85E-13 | 1.21E-10 |
| POU6F2 | 1.29008 | 0.913848 | 0.000409 | 0.009798 |
| LINC02714 | 1.289147 | -3.3272 | 5.81E-05 | 0.002169 |
| IGF2BP2-AS1 | 1.288949 | -1.894118 | 4.47E-06 | 0.000267 |
| AL022067.1 | 1.28852 | -2.462218 | 3.03E-08 | 3.71E-06 |
| MTCO1P40 | 1.288339 | 2.813721 | 1.13E-05 | 0.000575 |
| PBOV1 | 1.284846 | -2.648852 | 0.00214 | 0.032207 |
| GBX2 | 1.283573 | -1.360797 | 4.27E-06 | 0.000256 |
| LINC01287 | 1.282042 | -3.124959 | 6.96E-07 | 5.62E-05 |
| AL513318.2 | 1.282038 | -1.265924 | 2.69E-06 | 0.000175 |
| AL645504.1 | 1.280466 | -3.194625 | 4.46E-05 | 0.001738 |
| AC018463.1 | 1.279149 | -2.787983 | 0.000209 | 0.005777 |
| IGSF1 | 1.278663 | -0.124504 | 2.67E-07 | 2.43E-05 |
| CHGA | 1.278567 | 5.246041 | 0.000352 | 0.008682 |
| RPS16P5 | 1.277922 | -3.50665 | 0.001071 | 0.019901 |
| KCNH5 | 1.277162 | -3.31491 | 0.001205 | 0.021528 |
| RNU6-130P | 1.276532 | -3.422698 | 0.000327 | 0.00821 |
| LINC02656 | 1.275134 | -1.682473 | 4.81E-12 | 1.24E-09 |
| MARCHF4 | 1.271734 | -0.753174 | 8.63E-14 | 2.96E-11 |
| NKX6-1 | 1.271249 | -2.793952 | 0.001108 | 0.020363 |
| CGB8 | 1.27018 | -3.61439 | 0.001683 | 0.027514 |
| KRT39 | 1.269946 | -1.977072 | 3.32E-05 | 0.001375 |
| LY6G6C | 1.26929 | 0.493087 | 4.71E-09 | 6.65E-07 |
| DRD5 | 1.268033 | -1.016357 | 0.001239 | 0.021852 |
| SYT6 | 1.265422 | -2.527124 | 2.08E-06 | 0.000142 |
| AC091151.1 | 1.264959 | -3.121836 | 0.001181 | 0.021282 |
| AL589743.5 | 1.264282 | -3.369299 | 0.000792 | 0.016005 |
| RPL7P25 | 1.262813 | -3.395162 | 0.000288 | 0.00745 |
| HSD3BP5 | 1.262291 | -2.045693 | 2.49E-09 | 3.71E-07 |
| PGPEP1L | 1.259854 | -3.302974 | 0.000471 | 0.010862 |
| AKR1C4 | 1.259353 | 0.439206 | 8.85E-06 | 0.000465 |
| GABRQ | 1.258631 | -2.25099 | 1.75E-08 | 2.26E-06 |
| AC004080.1 | 1.258519 | -0.851055 | 7.38E-05 | 0.002636 |
| SHOC1 | 1.256454 | -0.986611 | 2.63E-05 | 0.001136 |
| AC106865.1 | 1.25622 | -2.074406 | 2.82E-06 | 0.000183 |
| CHST9 | 1.254863 | -2.244706 | 0.000512 | 0.011533 |
| AC079866.1 | 1.252 | -3.300571 | 0.00087 | 0.017059 |
| AC020661.3 | 1.250927 | -3.313411 | 3.98E-05 | 0.001585 |
| LINC02600 | 1.249504 | -0.722943 | 6.06E-08 | 6.90E-06 |
| MTND1P23 | 1.248378 | 4.07527 | 0.000781 | 0.015858 |
| AC022973.2 | 1.247309 | -3.427027 | 0.001886 | 0.029729 |
| ST7-AS2 | 1.244597 | -2.596343 | 0.003391 | 0.044151 |
| MAPRE3-AS1 | 1.243291 | -2.921999 | 1.34E-06 | 9.82E-05 |
| Z97200.1 | 1.241824 | -2.261283 | 7.80E-06 | 0.00042 |
| LY6K | 1.241477 | -2.447327 | 5.54E-07 | 4.63E-05 |
| OR51B5 | 1.241422 | -2.846448 | 0.002795 | 0.038709 |
| AC107021.1 | 1.241136 | -2.801467 | 2.67E-06 | 0.000175 |
| SH3GL3 | 1.239833 | -3.350031 | 0.000522 | 0.011716 |
| MIR663AHG | 1.23869 | -2.893707 | 0.000547 | 0.012112 |
| GNA14-AS1 | 1.237228 | -3.492847 | 3.61E-05 | 0.001467 |
| AC068580.2 | 1.236745 | -2.352691 | 0.000106 | 0.003489 |
| ACMSD | 1.233969 | -2.353128 | 1.17E-05 | 0.00059 |
| AL117190.1 | 1.232082 | -2.732949 | 6.56E-06 | 0.000365 |
| AC011899.1 | 1.231989 | -3.218266 | 0.000506 | 0.011456 |
| SYT16 | 1.231967 | -3.084459 | 1.71E-06 | 0.00012 |
| AC073569.3 | 1.231241 | -2.585611 | 4.66E-06 | 0.000276 |
| TPTEP1 | 1.22924 | 0.848935 | 1.95E-10 | 3.96E-08 |
| LINC01709 | 1.228039 | -3.430597 | 0.00199 | 0.030794 |
| KLF14 | 1.227724 | -2.990164 | 3.72E-06 | 0.000229 |
| TMEM114 | 1.22698 | -3.222759 | 5.51E-05 | 0.002078 |
| AL353746.1 | 1.224977 | -3.496525 | 1.46E-05 | 0.000713 |
| CFAP61-AS1 | 1.224929 | -3.546439 | 2.48E-05 | 0.001083 |
| BTN1A1 | 1.223363 | -0.081262 | 2.14E-06 | 0.000145 |
| CCDC169 | 1.22321 | -1.527306 | 0.000144 | 0.004413 |
| SCRT1 | 1.221996 | -2.587405 | 1.44E-05 | 0.000705 |
| AC012494.1 | 1.221625 | -3.459982 | 0.000123 | 0.003909 |
| KCNN1 | 1.220959 | -1.795858 | 3.48E-10 | 6.85E-08 |
| CPA4 | 1.220854 | -1.126858 | 5.44E-07 | 4.57E-05 |
| KCNJ6 | 1.220136 | -1.927134 | 1.07E-07 | 1.11E-05 |
| JPH3 | 1.218625 | -0.994334 | 4.58E-08 | 5.33E-06 |
| H2AC9P | 1.21849 | -3.580358 | 1.36E-05 | 0.000668 |
| SLCO1A2 | 1.218277 | -1.432184 | 0.000196 | 0.005516 |
| KCNQ2 | 1.217882 | -0.055538 | 0.000599 | 0.013074 |
| KRT7 | 1.216373 | 4.030384 | 3.69E-06 | 0.000228 |
| ZNF90P1 | 1.216026 | -3.291246 | 0.002042 | 0.031275 |
| AL390778.2 | 1.215929 | -2.151164 | 0.001285 | 0.022387 |
| PLIN1 | 1.21493 | 1.320189 | 5.31E-06 | 0.000308 |
| LINC02302 | 1.214622 | -3.562723 | 0.002864 | 0.039378 |
| KCNB2 | 1.212697 | -2.822616 | 7.56E-07 | 6.02E-05 |
| AC098850.3 | 1.212136 | -2.822489 | 7.55E-05 | 0.002679 |
| LINC00494 | 1.210894 | -1.415324 | 3.30E-06 | 0.000208 |
| LCN15 | 1.210544 | 5.416324 | 0.001855 | 0.029382 |
| KCNMA1-AS1 | 1.209502 | -3.013108 | 7.69E-05 | 0.002721 |
| AC016027.2 | 1.208658 | -2.920122 | 8.34E-06 | 0.000442 |
| ZNF334 | 1.207471 | 1.147899 | 5.77E-06 | 0.000329 |
| KRT40 | 1.207429 | 1.35423 | 0.000283 | 0.00736 |
| AL138955.1 | 1.204709 | -2.252002 | 3.21E-06 | 0.000204 |
| TIMM9P2 | 1.203132 | -3.148633 | 0.000878 | 0.01713 |
| CACNG4 | 1.202812 | 2.279701 | 0.00013 | 0.004083 |
| AC234772.2 | 1.201389 | -2.484565 | 0.002497 | 0.035679 |
| AC026336.3 | 1.201075 | 0.951889 | 0.001444 | 0.024541 |
| GABRG2 | 1.200343 | -2.240386 | 0.000869 | 0.017052 |
| AL356274.2 | 1.198743 | -3.069072 | 0.001614 | 0.026576 |
| AL034418.1 | 1.196068 | -3.11853 | 0.000907 | 0.017584 |
| SNCB | 1.195691 | -2.83606 | 2.21E-05 | 0.000996 |
| OPCML | 1.195011 | -0.952095 | 9.18E-09 | 1.22E-06 |
| FOXI1 | 1.190964 | -0.449908 | 0.001025 | 0.019214 |
| AL358473.1 | 1.188666 | -2.847007 | 9.22E-10 | 1.55E-07 |
| MST1L | 1.186202 | 0.079698 | 4.85E-09 | 6.81E-07 |
| LINC01694 | 1.185876 | 0.539973 | 1.48E-06 | 0.000106 |
| GHRH | 1.18583 | -3.487223 | 0.000216 | 0.005933 |
| AL360091.3 | 1.182956 | -3.521128 | 0.002964 | 0.040298 |
| AC107308.1 | 1.182851 | -3.164264 | 6.76E-06 | 0.000374 |
| TAS2R63P | 1.182757 | -3.325219 | 0.003113 | 0.041731 |
| C1orf61 | 1.182612 | -1.615886 | 7.39E-06 | 0.000403 |
| AC087286.4 | 1.181073 | -3.009263 | 0.001735 | 0.028053 |
| PICSAR | 1.179459 | -2.509425 | 0.00011 | 0.003591 |
| AC009268.1 | 1.179092 | -3.442732 | 0.003061 | 0.041264 |
| CR392039.3 | 1.178457 | -3.408194 | 0.000383 | 0.009246 |
| ASTN1 | 1.178331 | -1.499971 | 0.000294 | 0.007568 |
| AL590762.4 | 1.177038 | -3.262496 | 0.000499 | 0.01135 |
| C11orf97 | 1.173989 | -3.4815 | 5.28E-05 | 0.002002 |
| SULT1E1 | 1.172223 | 0.726715 | 0.000637 | 0.013637 |
| NKX2-5 | 1.170054 | -2.904499 | 0.001242 | 0.021881 |
| INSL6 | 1.168346 | -3.334732 | 0.003034 | 0.041046 |
| MIR133A1HG | 1.165153 | -1.951797 | 0.001955 | 0.030408 |
| AP001042.2 | 1.16322 | -3.598001 | 0.000216 | 0.005936 |
| LINC02495 | 1.163117 | -3.464194 | 2.15E-05 | 0.000981 |
| AC100774.1 | 1.162464 | -3.058194 | 0.000419 | 0.00994 |
| AL391840.2 | 1.161299 | -3.631799 | 0.002465 | 0.035382 |
| TMEM59L | 1.159923 | -0.342742 | 1.61E-06 | 0.000114 |
| SERPINB2 | 1.159829 | 0.694038 | 0.001178 | 0.021261 |
| PCDHB17P | 1.158554 | -3.371076 | 3.66E-05 | 0.001478 |
| GALNT14 | 1.158186 | 0.314505 | 1.05E-05 | 0.000541 |
| RN7SKP78 | 1.156388 | -3.368122 | 0.000373 | 0.009069 |
| ALOX12P2 | 1.15545 | -0.782932 | 0.000111 | 0.003605 |
| PTTG4P | 1.154235 | -3.623793 | 0.00032 | 0.008103 |
| SCEL | 1.153816 | 1.728846 | 6.97E-05 | 0.002519 |
| CALB1 | 1.150149 | 3.365361 | 0.003475 | 0.044862 |
| INSM1 | 1.150088 | 1.226883 | 0.000153 | 0.00459 |
| PCDH9 | 1.148029 | -0.007262 | 5.73E-07 | 4.76E-05 |
| YWHAQP5 | 1.147172 | -3.401247 | 0.002423 | 0.034975 |
| AC012501.2 | 1.146579 | -2.154763 | 0.001606 | 0.026527 |
| QRSL1P3 | 1.146545 | -2.789792 | 0.002315 | 0.033917 |
| SLC7A11-AS1 | 1.145814 | -1.67939 | 1.70E-06 | 0.000119 |
| PLCXD3 | 1.144107 | -1.074209 | 0.000338 | 0.00842 |
| AC114760.2 | 1.144049 | -1.688508 | 5.58E-06 | 0.000319 |
| TCL6 | 1.143509 | -0.791393 | 0.00069 | 0.01445 |
| RPS27AP7 | 1.136556 | -3.276391 | 0.003927 | 0.04878 |
| AC025575.2 | 1.134979 | -1.418257 | 8.41E-08 | 9.07E-06 |
| AL138689.1 | 1.134697 | -0.868828 | 1.47E-08 | 1.93E-06 |
| AC021739.4 | 1.134457 | -3.464993 | 0.000955 | 0.018279 |
| RN7SL600P | 1.132669 | -2.860336 | 6.51E-05 | 0.002383 |
| HAO2 | 1.132435 | -3.382191 | 4.71E-06 | 0.000278 |
| KCNT1 | 1.127638 | -0.08002 | 1.91E-05 | 0.000887 |
| AL845331.2 | 1.127367 | -3.606444 | 0.000104 | 0.003431 |
| CNGA3 | 1.125804 | -0.569267 | 0.00012 | 0.00384 |
| SPTSSB | 1.125692 | 0.566601 | 3.34E-05 | 0.001378 |
| SYT5 | 1.124775 | -0.77763 | 3.75E-07 | 3.29E-05 |
| S100Z | 1.123975 | -2.738677 | 3.17E-08 | 3.82E-06 |
| C12orf56 | 1.123615 | -0.435741 | 0.001705 | 0.027731 |
| DGKB | 1.119315 | 0.139097 | 0.000411 | 0.009828 |
| EPHB6 | 1.117735 | 3.365668 | 9.89E-05 | 0.003297 |
| Z93403.1 | 1.116629 | -3.26901 | 0.00123 | 0.021742 |
| FOXC2 | 1.116016 | -0.12028 | 2.08E-08 | 2.66E-06 |
| SLC30A3 | 1.114872 | -1.977207 | 4.39E-07 | 3.81E-05 |
| AC006116.6 | 1.114169 | -3.326519 | 0.000637 | 0.013637 |
| FAM83A | 1.114153 | 1.592887 | 2.66E-05 | 0.001146 |
| PRMT8 | 1.111658 | -3.08797 | 0.000234 | 0.006331 |
| NKAIN4 | 1.111209 | -0.95309 | 2.15E-08 | 2.74E-06 |
| AVPR2 | 1.11038 | -0.205486 | 8.97E-08 | 9.51E-06 |
| AC022395.1 | 1.110202 | -2.520982 | 0.000234 | 0.006331 |
| RIPPLY2 | 1.110038 | -3.259109 | 0.00322 | 0.042596 |
| AC103702.1 | 1.109353 | -1.865843 | 4.47E-05 | 0.001742 |
| AC022336.4 | 1.107526 | -2.765486 | 0.000381 | 0.009195 |
| KCNK15 | 1.107425 | 0.740454 | 2.15E-06 | 0.000145 |
| AC007319.1 | 1.107188 | -2.711795 | 0.000151 | 0.00454 |
| ERBB4 | 1.102866 | -2.861964 | 0.000346 | 0.008574 |
| KRR1P1 | 1.10102 | -1.730946 | 3.76E-05 | 0.001518 |
| APLP1 | 1.099558 | 1.010754 | 4.14E-10 | 8.10E-08 |
| AC008759.2 | 1.098039 | -2.59163 | 5.28E-08 | 6.06E-06 |
| PLPPR3 | 1.097523 | -2.351006 | 6.32E-05 | 0.002328 |
| DISC1FP1 | 1.094475 | -3.062206 | 0.000752 | 0.015366 |
| AC022467.1 | 1.092415 | -3.522836 | 9.44E-05 | 0.003187 |
| LINC01602 | 1.091748 | -1.050535 | 0.002213 | 0.032951 |
| DUTP1 | 1.091464 | -3.310418 | 0.000651 | 0.013884 |
| AC090673.1 | 1.090432 | -3.55943 | 0.000737 | 0.015158 |
| AC079035.1 | 1.087583 | -3.253194 | 0.003419 | 0.04441 |
| AL591222.1 | 1.087099 | -2.888859 | 0.001184 | 0.021288 |
| ARL4AP4 | 1.085556 | -2.933509 | 6.73E-05 | 0.002444 |
| AC048351.1 | 1.085089 | -3.19945 | 0.002406 | 0.034833 |
| Z99289.2 | 1.084173 | -2.949972 | 0.000156 | 0.004662 |
| RAPGEF4-AS1 | 1.081679 | -3.53428 | 0.000856 | 0.016897 |
| PRB2 | 1.078841 | -3.2077 | 0.002699 | 0.037653 |
| AL022238.1 | 1.078317 | -3.313748 | 0.000392 | 0.009403 |
| ZNF98 | 1.076802 | -3.606283 | 0.000108 | 0.003542 |
| AC009269.2 | 1.076169 | -3.496585 | 0.001174 | 0.021206 |
| CACNA1C-AS2 | 1.07432 | -2.192277 | 1.35E-07 | 1.36E-05 |
| ALX4 | 1.073974 | -2.963904 | 0.000108 | 0.003542 |
| VEGFD | 1.072793 | -0.818741 | 0.000141 | 0.004332 |
| ATP2B2 | 1.070886 | -2.355999 | 2.30E-05 | 0.001028 |
| LINC01122 | 1.069662 | -3.581761 | 1.29E-05 | 0.000639 |
| TTYH1 | 1.06952 | 0.560954 | 3.04E-08 | 3.71E-06 |
| AL161658.1 | 1.068059 | -2.983672 | 0.003518 | 0.045154 |
| AP000525.1 | 1.067884 | -2.077781 | 0.000219 | 0.005978 |
| GK-AS1 | 1.067668 | -1.273206 | 5.10E-08 | 5.87E-06 |
| HSPE1P26 | 1.067638 | -3.394724 | 0.000363 | 0.008875 |
| AC007684.1 | 1.065902 | -3.272471 | 0.000542 | 0.012056 |
| AC087392.1 | 1.063054 | -3.000561 | 4.91E-05 | 0.00188 |
| RDH10-AS1 | 1.06252 | -1.139976 | 1.52E-07 | 1.49E-05 |
| AP005432.1 | 1.062114 | -2.764214 | 9.02E-05 | 0.003084 |
| AC104958.1 | 1.061041 | -2.586085 | 0.000407 | 0.009743 |
| AC027808.2 | 1.060735 | -2.926016 | 4.47E-06 | 0.000267 |
| AC073912.3 | 1.05891 | -3.421307 | 0.000146 | 0.004457 |
| AC079313.1 | 1.057238 | -2.973846 | 0.000156 | 0.004667 |
| BBOX1 | 1.057046 | -2.017659 | 0.000544 | 0.012064 |
| LINC00862 | 1.056284 | -2.985946 | 3.17E-06 | 0.000201 |
| TMEM215 | 1.055515 | -3.111781 | 0.000197 | 0.005538 |
| CBX5P1 | 1.052471 | -3.418016 | 0.003132 | 0.041794 |
| DUSP9 | 1.051092 | -0.239693 | 0.00014 | 0.00432 |
| HEPHL1 | 1.050116 | 0.936734 | 0.000127 | 0.004006 |
| SVOP | 1.049639 | -1.082541 | 0.000362 | 0.008863 |
| ANKRD18DP | 1.049292 | -3.583168 | 8.61E-05 | 0.002976 |
| FBXW7-AS1 | 1.047171 | -3.608069 | 0.002134 | 0.032195 |
| ITPRIP-AS1 | 1.044169 | -2.718732 | 9.88E-06 | 0.00051 |
| ELFN2 | 1.04269 | 0.43007 | 4.95E-05 | 0.001894 |
| TBL1XR1-AS1 | 1.040895 | -2.795564 | 0.002567 | 0.036406 |
| LINC01170 | 1.037569 | -3.12392 | 0.003554 | 0.045318 |
| CTXND1 | 1.03501 | -0.1985 | 0.000516 | 0.011611 |
| AC012501.3 | 1.034676 | -0.198261 | 0.004011 | 0.04948 |
| MEG8 | 1.033735 | -2.866044 | 0.000179 | 0.005188 |
| LGALS7B | 1.032836 | -2.216452 | 9.15E-05 | 0.003115 |
| SLC24A2 | 1.031111 | -0.806677 | 1.81E-05 | 0.00085 |
| PROKR1 | 1.030314 | -3.039822 | 0.000119 | 0.003823 |
| MIR31HG | 1.029948 | -1.857882 | 0.001638 | 0.026873 |
| AC078925.4 | 1.028679 | -2.315849 | 0.001139 | 0.020757 |
| ZNF883 | 1.027974 | -0.45304 | 1.11E-05 | 0.000567 |
| CNIH2 | 1.026416 | -0.817685 | 2.55E-12 | 6.83E-10 |
| MYO3A | 1.026357 | -1.514332 | 0.000209 | 0.005777 |
| AC090971.3 | 1.025625 | -3.450766 | 0.001193 | 0.021375 |
| CACNA1G | 1.024264 | -1.669492 | 2.65E-07 | 2.43E-05 |
| AC010624.1 | 1.023745 | -2.927158 | 7.53E-05 | 0.002676 |
| LINC00566 | 1.023744 | -3.597468 | 0.000419 | 0.00994 |
| GABRA4 | 1.022894 | 0.541218 | 0.001622 | 0.026668 |
| AC112496.1 | 1.021977 | -1.64943 | 3.54E-07 | 3.12E-05 |
| AC015799.1 | 1.020622 | -1.225745 | 2.19E-05 | 0.000992 |
| PCDHB6 | 1.019787 | -1.394926 | 1.46E-06 | 0.000106 |
| ANGPTL5 | 1.019532 | -3.15936 | 0.000341 | 0.008478 |
| AC018641.1 | 1.017713 | -1.922793 | 0.000131 | 0.004097 |
| IFNWP19 | 1.017374 | -2.238865 | 0.002379 | 0.034519 |
| AC004917.1 | 1.014327 | -3.174814 | 1.34E-05 | 0.000662 |
| AL358232.1 | 1.014087 | -2.669906 | 0.00151 | 0.025246 |
| LINC00643 | 1.013417 | -3.365335 | 8.76E-05 | 0.003016 |
| AC005072.1 | 1.012097 | -2.696277 | 0.002121 | 0.032028 |
| FLG-AS1 | 1.01172 | -1.764658 | 2.44E-06 | 0.00016 |
| DLGAP1 | 1.011308 | -0.458162 | 3.84E-06 | 0.000235 |
| MAL2-AS1 | 1.009827 | -1.683272 | 3.71E-06 | 0.000229 |
| CDK5R2 | 1.009148 | -0.576186 | 0.000209 | 0.005791 |
| KRT8P17 | 1.008822 | -1.6102 | 9.02E-05 | 0.003084 |
| AC013394.1 | 1.00748 | -0.609013 | 9.35E-08 | 9.87E-06 |
| MRPL23-AS1 | 1.006728 | -1.808918 | 0.002044 | 0.031276 |
| ZNF365 | 1.005307 | -1.227429 | 2.44E-06 | 0.00016 |
| AC023051.1 | 1.004071 | -2.863392 | 0.000157 | 0.00469 |
| AC023157.1 | -1.007048 | -0.139067 | 0.000487 | 0.01114 |
| H2AC16 | -1.01337 | -2.191276 | 0.004047 | 0.049621 |
| TFF1 | -1.013484 | 7.268737 | 0.001116 | 0.020475 |
| ART3 | -1.013762 | 1.461283 | 0.000751 | 0.015366 |
| GLYATL2 | -1.015264 | -1.322754 | 0.003966 | 0.04913 |
| CALML3 | -1.015353 | -0.085309 | 0.002903 | 0.039725 |
| AC006970.1 | -1.016406 | -1.614637 | 0.000452 | 0.010507 |
| TRIM7 | -1.01824 | 3.082173 | 0.000384 | 0.009246 |
| GZMA | -1.020244 | 2.9475 | 5.34E-06 | 0.00031 |
| RPL15P18 | -1.020709 | -1.817648 | 7.92E-05 | 0.00279 |
| HLA-DPA3 | -1.02077 | -3.145626 | 0.000578 | 0.012681 |
| RPS2P2 | -1.021364 | -3.176756 | 0.000586 | 0.012813 |
| ENPEP | -1.022418 | 3.239525 | 7.17E-05 | 0.002576 |
| H2AC7 | -1.024023 | -0.145255 | 0.000652 | 0.013887 |
| MTHFD2P7 | -1.024149 | -3.405093 | 0.001448 | 0.024586 |
| RPS26P47 | -1.027484 | -1.670556 | 0.000438 | 0.010259 |
| LINC02489 | -1.027569 | -1.665454 | 0.00025 | 0.00663 |
| KRT2 | -1.027967 | -2.680321 | 0.00158 | 0.026181 |
| UROC1 | -1.029883 | -2.864979 | 0.00215 | 0.032337 |
| UGT2B7 | -1.031851 | 2.784846 | 0.000581 | 0.012726 |
| H3C7 | -1.034863 | -1.64889 | 0.002697 | 0.037636 |
| ABCG5 | -1.03543 | 0.379723 | 0.000508 | 0.01149 |
| SLC25A5P8 | -1.036978 | -3.471597 | 0.000224 | 0.006093 |
| LINC00114 | -1.037401 | 0.542923 | 2.37E-05 | 0.001048 |
| LAIR2 | -1.039853 | -0.258866 | 0.000152 | 0.004565 |
| AC004224.1 | -1.041248 | -2.001507 | 0.002466 | 0.035382 |
| RFPL2 | -1.053276 | -2.862964 | 0.004046 | 0.049621 |
| SLITRK6 | -1.054821 | 3.258341 | 0.002107 | 0.031924 |
| H3P1 | -1.055594 | -3.141105 | 0.000135 | 0.004217 |
| FASLG | -1.055843 | 0.028139 | 1.46E-06 | 0.000106 |
| CALHM6 | -1.057336 | 2.347878 | 4.85E-07 | 4.17E-05 |
| AL357632.1 | -1.057627 | -2.955334 | 0.001497 | 0.025125 |
| PGDP1 | -1.059148 | -2.567732 | 0.00071 | 0.014755 |
| AC005165.2 | -1.05999 | -1.609014 | 0.002963 | 0.040296 |
| VNN1 | -1.060088 | 3.855575 | 0.001119 | 0.020508 |
| APOBEC3A | -1.06065 | 0.361598 | 0.000128 | 0.004035 |
| GBP1P1 | -1.063992 | 0.64154 | 7.88E-06 | 0.000423 |
| RPSAP49 | -1.064108 | -1.288671 | 7.39E-05 | 0.002636 |
| SMLR1 | -1.073644 | -2.264344 | 0.001183 | 0.021288 |
| GAPDHP38 | -1.073902 | -0.871942 | 0.000145 | 0.00442 |
| LINC00261 | -1.081114 | 5.360053 | 0.000183 | 0.005268 |
| AC104248.1 | -1.084704 | -2.702414 | 0.000799 | 0.01609 |
| IGKV2D-29 | -1.086774 | 2.255494 | 0.00204 | 0.031275 |
| MTND4P20 | -1.089007 | -1.727293 | 0.000938 | 0.01803 |
| RHOXF1-AS1 | -1.089826 | -0.298799 | 0.000482 | 0.011072 |
| AL122018.1 | -1.09487 | -2.554841 | 0.00025 | 0.006641 |
| RPL3P12 | -1.095609 | -2.286805 | 0.000357 | 0.008774 |
| RPL29P11 | -1.095763 | -0.37673 | 1.97E-06 | 0.000135 |
| RBP2 | -1.096723 | 2.746664 | 0.001765 | 0.02844 |
| MTCYBP18 | -1.098836 | 0.659802 | 0.001837 | 0.029222 |
| RPSAP63 | -1.099905 | -2.962872 | 0.001809 | 0.028979 |
| NDUFB1P1 | -1.10124 | -2.198682 | 0.002178 | 0.032579 |
| CYP2C9 | -1.102096 | 0.05378 | 0.000443 | 0.010344 |
| SAA1 | -1.102242 | 4.06347 | 0.001711 | 0.027785 |
| AC046168.2 | -1.102387 | -2.591829 | 0.000856 | 0.016897 |
| AF124730.2 | -1.109094 | -3.046663 | 0.000815 | 0.016302 |
| AP002433.1 | -1.114799 | -3.25658 | 0.003717 | 0.046744 |
| AC005336.2 | -1.114915 | -3.292225 | 0.002266 | 0.033494 |
| SLC18A1 | -1.11623 | 1.796546 | 0.001735 | 0.028053 |
| AP001099.1 | -1.121027 | -3.584155 | 0.002139 | 0.032207 |
| PNPLA3 | -1.125623 | 0.625705 | 0.000236 | 0.006354 |
| AC007160.1 | -1.131605 | -1.29787 | 0.002038 | 0.031275 |
| AP006565.1 | -1.133221 | -3.128646 | 0.001578 | 0.026181 |
| AP003057.1 | -1.136396 | -2.188927 | 1.77E-05 | 0.000833 |
| ACTG1P19 | -1.137269 | -2.5701 | 0.000419 | 0.00994 |
| AL008733.1 | -1.139804 | -3.349831 | 0.002347 | 0.034279 |
| AC126564.1 | -1.141719 | -2.458661 | 0.001689 | 0.027556 |
| SLC25A5P6 | -1.142274 | -3.183139 | 7.35E-06 | 0.000402 |
| HLA-V | -1.146274 | -0.174655 | 0.000977 | 0.018559 |
| MTND6P22 | -1.148474 | -3.11326 | 0.003344 | 0.043742 |
| LINC02528 | -1.149418 | -3.32976 | 0.00137 | 0.023564 |
| HLA-DRB9 | -1.150751 | -2.430678 | 1.91E-05 | 0.000887 |
| CD1B | -1.154406 | -1.367513 | 1.13E-05 | 0.000572 |
| SAA2 | -1.155383 | 2.036146 | 0.000656 | 0.013942 |
| AC068658.1 | -1.155578 | -2.78812 | 0.000124 | 0.003947 |
| CXCL11 | -1.155627 | 4.173248 | 4.70E-05 | 0.001822 |
| RN7SL396P | -1.156291 | -2.453363 | 0.001872 | 0.029584 |
| AF124730.1 | -1.161286 | -3.355967 | 0.001511 | 0.025246 |
| H2AC13 | -1.162371 | -0.142387 | 0.00042 | 0.00994 |
| HSF5 | -1.164415 | -2.535223 | 0.001975 | 0.030636 |
| ALOX15B | -1.171538 | 1.269028 | 3.16E-05 | 0.001323 |
| C2CD4B | -1.171673 | 3.124088 | 4.94E-06 | 0.000289 |
| TFAP2A-AS1 | -1.17389 | -0.084015 | 3.64E-05 | 0.001477 |
| H2AC17 | -1.173925 | -1.033942 | 0.000221 | 0.006018 |
| TCHH | -1.174889 | 0.280186 | 0.00098 | 0.018594 |
| SNORA71A | -1.180706 | -0.17785 | 0.000613 | 0.013301 |
| PRDX2P1 | -1.18472 | -2.807694 | 0.001402 | 0.02394 |
| OTOG | -1.188211 | -2.397248 | 0.000997 | 0.018868 |
| H2BC7 | -1.188645 | -0.753945 | 0.001094 | 0.020217 |
| GNLY | -1.188814 | 2.907832 | 3.97E-07 | 3.47E-05 |
| ALDOAP1 | -1.190584 | -2.074295 | 0.000122 | 0.003889 |
| GPR25 | -1.191245 | -1.17585 | 1.06E-05 | 0.000542 |
| MMP12 | -1.195238 | 6.061874 | 9.52E-07 | 7.36E-05 |
| IGKV2D-40 | -1.202244 | 0.66239 | 0.002034 | 0.031275 |
| GAPDHP21 | -1.213631 | -2.055521 | 4.17E-05 | 0.001649 |
| RNVU1-27 | -1.216965 | -1.996983 | 0.002369 | 0.034455 |
| AADAC | -1.220369 | 0.633038 | 0.001537 | 0.025613 |
| SCARNA22 | -1.220654 | -2.38729 | 0.000219 | 0.005981 |
| CIITA | -1.225793 | 4.623659 | 1.40E-08 | 1.84E-06 |
| C11orf21 | -1.229967 | 0.095877 | 1.45E-05 | 0.000707 |
| ASS1P9 | -1.23089 | -2.12201 | 2.40E-05 | 0.001057 |
| LINC02446 | -1.231451 | -1.048548 | 0.000158 | 0.004699 |
| AC067904.2 | -1.232735 | -3.120995 | 0.000413 | 0.00986 |
| SCARNA6 | -1.24558 | 0.034834 | 0.003391 | 0.044151 |
| FDPSP1 | -1.256288 | -2.734459 | 0.000236 | 0.006348 |
| BANCR | -1.256993 | -3.090214 | 0.000177 | 0.005162 |
| MTCO3P12 | -1.261353 | 3.795551 | 0.000437 | 0.010253 |
| C2CD4A | -1.262211 | 4.789256 | 3.15E-07 | 2.80E-05 |
| AL353705.3 | -1.264524 | -2.243706 | 6.42E-05 | 0.002358 |
| CXCR2P1 | -1.265557 | 0.214924 | 4.23E-06 | 0.000255 |
| LINC02195 | -1.269568 | -2.244388 | 2.85E-06 | 0.000184 |
| AC008753.1 | -1.271333 | -1.661906 | 4.04E-06 | 0.000245 |
| ASS1P7 | -1.276365 | -2.868744 | 0.000445 | 0.01037 |
| SNORA80B | -1.285935 | -1.919776 | 0.000824 | 0.01644 |
| MT-TH | -1.287196 | -3.126086 | 0.002364 | 0.03443 |
| RN7SL2 | -1.288178 | 6.52895 | 0.00017 | 0.004986 |
| CLEC6A | -1.29551 | -2.383166 | 2.74E-05 | 0.001169 |
| SERPINA1 | -1.296567 | 8.741005 | 2.95E-07 | 2.63E-05 |
| GBP5 | -1.300735 | 3.857242 | 1.27E-07 | 1.30E-05 |
| PLA2G3 | -1.308861 | 1.301452 | 0.001979 | 0.030656 |
| BEST3 | -1.309938 | -0.565278 | 0.001166 | 0.021108 |
| H2AC12 | -1.310778 | -1.681752 | 0.000623 | 0.013461 |
| MUC5B | -1.319205 | 8.498722 | 5.18E-05 | 0.001971 |
| MYBPC3 | -1.320757 | -0.811683 | 6.34E-06 | 0.000356 |
| MT-TM | -1.328266 | 0.747186 | 0.001189 | 0.021345 |
| CNDP1 | -1.331949 | -0.875658 | 0.000377 | 0.009119 |
| NBPF7 | -1.33814 | -1.604084 | 0.00032 | 0.008103 |
| SBSN | -1.339122 | -1.172559 | 0.000854 | 0.016897 |
| ALDH1A2 | -1.342523 | 2.237954 | 0.000516 | 0.01161 |
| M1AP | -1.356568 | -0.975806 | 1.35E-05 | 0.000664 |
| UBD | -1.360221 | 4.215189 | 1.49E-07 | 1.46E-05 |
| SLC25A6P2 | -1.362968 | 0.401292 | 0.000791 | 0.015996 |
| H2BC13 | -1.367751 | -1.095522 | 1.64E-05 | 0.000787 |
| AC093063.1 | -1.371836 | -3.12217 | 0.001096 | 0.020233 |
| AC100826.1 | -1.374594 | -3.046942 | 0.001912 | 0.029992 |
| PIWIL1 | -1.37782 | 3.083299 | 0.000296 | 0.00762 |
| GBP4 | -1.381289 | 5.572132 | 2.40E-09 | 3.60E-07 |
| AL162739.2 | -1.383662 | -2.766823 | 0.002663 | 0.037346 |
| NOS2 | -1.401137 | 6.100787 | 1.06E-07 | 1.10E-05 |
| KIR3DL1 | -1.405426 | -3.152236 | 0.000332 | 0.008316 |
| AC008514.1 | -1.408572 | -1.621783 | 0.000703 | 0.014635 |
| DMBT1 | -1.413839 | 8.599129 | 4.37E-05 | 0.001711 |
| NLRP6 | -1.425282 | 1.56252 | 3.09E-06 | 0.000198 |
| AC005515.1 | -1.43748 | -0.490455 | 3.59E-05 | 0.001461 |
| PPIAP43 | -1.449635 | -2.293744 | 1.51E-05 | 0.000732 |
| SNORD17 | -1.467125 | 1.516671 | 3.09E-05 | 0.001296 |
| LINC01924 | -1.467722 | -3.533343 | 0.001684 | 0.027514 |
| PYDC1 | -1.469049 | -2.802177 | 0.001086 | 0.020119 |
| AC007991.4 | -1.469797 | -2.98182 | 0.000273 | 0.007143 |
| ZNF683 | -1.47848 | 0.612746 | 6.47E-07 | 5.32E-05 |
| SNHG25 | -1.479804 | 2.951414 | 1.56E-05 | 0.000753 |
| ATP12A | -1.490489 | 0.688286 | 0.003122 | 0.041779 |
| ITLN2 | -1.495517 | 0.556183 | 0.001918 | 0.03005 |
| RN7SL5P | -1.500389 | -1.526126 | 8.54E-05 | 0.002963 |
| AC129492.6 | -1.503109 | -2.557718 | 0.000632 | 0.013591 |
| HABP2 | -1.504093 | 1.044393 | 0.00097 | 0.018494 |
| IFNG | -1.504253 | -1.163167 | 1.74E-07 | 1.69E-05 |
| REG4 | -1.50561 | 9.289864 | 0.000177 | 0.005158 |
| CXCL10 | -1.516833 | 4.978484 | 2.51E-08 | 3.16E-06 |
| H1-4 | -1.534839 | 0.863417 | 0.000186 | 0.00533 |
| CHRNA2 | -1.539692 | -3.05816 | 0.003453 | 0.044699 |
| CALCA | -1.541069 | 2.055285 | 0.00338 | 0.044056 |
| PCDHA1 | -1.543556 | -2.081557 | 0.000671 | 0.014198 |
| HLA-U | -1.549669 | -0.118625 | 3.22E-07 | 2.86E-05 |
| CXCL9 | -1.552493 | 5.281759 | 6.05E-09 | 8.24E-07 |
| IGLV3-22 | -1.558181 | -2.503518 | 0.000363 | 0.008875 |
| H3P16 | -1.57564 | -0.779028 | 3.20E-09 | 4.60E-07 |
| PCSK1 | -1.585154 | 5.100951 | 0.000148 | 0.004487 |
| L1TD1 | -1.587626 | 5.061224 | 5.18E-05 | 0.001971 |
| IL36B | -1.600056 | -2.197415 | 0.002222 | 0.033026 |
| KIF19 | -1.601969 | 1.97049 | 2.27E-06 | 0.000151 |
| PGLYRP3 | -1.60649 | -2.216463 | 0.000858 | 0.016901 |
| LINC01992 | -1.609682 | -3.049613 | 0.00313 | 0.041794 |
| GPR83 | -1.615806 | -0.34712 | 2.78E-05 | 0.001183 |
| WFDC21P | -1.616307 | 1.228202 | 5.23E-10 | 9.91E-08 |
| ADAD2 | -1.624203 | -2.158226 | 0.000274 | 0.00715 |
| LYPD2 | -1.625125 | -3.173625 | 0.002736 | 0.038044 |
| RPS24P13 | -1.643143 | -3.307241 | 2.32E-05 | 0.001036 |
| CCDC160 | -1.650323 | -2.453843 | 0.000376 | 0.009113 |
| AC104123.1 | -1.655675 | -2.889729 | 1.59E-05 | 0.000765 |
| BCAR4 | -1.659978 | -3.33822 | 0.001715 | 0.027812 |
| OR2I1P | -1.664199 | 3.979847 | 1.64E-09 | 2.64E-07 |
| REG1CP | -1.66866 | -3.392006 | 0.002296 | 0.033797 |
| AP000763.2 | -1.672238 | -0.766853 | 2.79E-06 | 0.000181 |
| SNORA53 | -1.678123 | 1.095314 | 0.000523 | 0.011733 |
| CRP | -1.679494 | -3.020481 | 0.002454 | 0.035275 |
| MUC6 | -1.693301 | 4.067763 | 0.000933 | 0.017949 |
| H4C5 | -1.695604 | 0.462274 | 6.35E-06 | 0.000356 |
| KIR2DS4 | -1.697415 | -3.064491 | 3.55E-05 | 0.001449 |
| H1-3 | -1.698018 | 0.123227 | 0.000108 | 0.003542 |
| IL17C | -1.745597 | -0.731907 | 1.74E-06 | 0.000121 |
| ENPP7 | -1.760117 | -2.016515 | 8.74E-05 | 0.003014 |
| IDO1 | -1.765258 | 4.769418 | 1.01E-07 | 1.06E-05 |
| PCA3 | -1.772548 | -2.02653 | 0.000108 | 0.003555 |
| KLK12 | -1.777906 | 2.662141 | 1.77E-06 | 0.000123 |
| H3C12 | -1.780566 | -0.860368 | 2.73E-05 | 0.001169 |
| ADCY8 | -1.783764 | -2.972349 | 0.000855 | 0.016897 |
| AC007368.1 | -1.788718 | -2.622014 | 0.000733 | 0.015113 |
| H2AC14 | -1.791616 | -1.271757 | 4.15E-05 | 0.001644 |
| TEX101 | -1.82318 | -1.332787 | 1.74E-06 | 0.000121 |
| IGFALS | -1.842561 | 1.44298 | 3.00E-06 | 0.000193 |
| TMCO2 | -1.846802 | -3.314454 | 0.00021 | 0.005803 |
| RN7SL3 | -1.85479 | 0.904447 | 9.57E-07 | 7.36E-05 |
| SNORA71D | -1.856563 | -1.064531 | 0.001978 | 0.030656 |
| TFF2 | -1.859928 | 3.983617 | 2.34E-05 | 0.001039 |
| MUC5AC | -1.894719 | 5.752863 | 3.85E-05 | 0.001541 |
| H4C13 | -1.908661 | -2.00974 | 0.002621 | 0.036924 |
| AC093001.1 | -1.913284 | -3.429856 | 0.000191 | 0.005419 |
| G6PC | -1.920685 | -0.66113 | 0.002823 | 0.038901 |
| ERVH48-1 | -1.926104 | -0.601134 | 1.08E-05 | 0.000549 |
| ANXA10 | -1.932383 | 1.475418 | 0.000558 | 0.012293 |
| H2BC17 | -1.934287 | -0.766798 | 3.63E-06 | 0.000226 |
| AC104809.1 | -1.936578 | -2.924512 | 0.000646 | 0.013785 |
| ALDOB | -1.942015 | 6.489923 | 2.26E-07 | 2.13E-05 |
| ANKRD30BP1 | -1.964309 | -3.105285 | 0.000475 | 0.010932 |
| CLCA2 | -1.971675 | 0.596032 | 3.39E-05 | 0.001396 |
| H3C11 | -2.001209 | -1.614825 | 4.88E-05 | 0.001873 |
| MTCO2P12 | -2.024865 | 3.344414 | 2.01E-10 | 4.07E-08 |
| SPINK4 | -2.036195 | 7.311198 | 7.29E-07 | 5.86E-05 |
| CTRB2 | -2.048864 | -3.202344 | 9.16E-05 | 0.003115 |
| LINC01630 | -2.057248 | -2.25202 | 2.48E-05 | 0.001082 |
| SNORA73B | -2.057445 | 3.117368 | 4.72E-06 | 0.000278 |
| ANKRD30B | -2.058245 | -1.427047 | 5.55E-06 | 0.000318 |
| CPA1 | -2.077622 | -2.104775 | 0.000813 | 0.016291 |
| CPS1 | -2.092337 | 4.494383 | 3.17E-06 | 0.000201 |
| IVL | -2.114177 | -1.006369 | 0.003594 | 0.045647 |
| H2AC21 | -2.125346 | -1.823195 | 3.89E-05 | 0.001551 |
| PAEPP1 | -2.128645 | -3.462812 | 0.001405 | 0.023964 |
| LINC01115 | -2.133339 | -2.561864 | 0.000404 | 0.009697 |
| MGAM | -2.154844 | 2.472897 | 2.78E-07 | 2.51E-05 |
| LIPF | -2.161589 | -2.800908 | 0.001553 | 0.025858 |
| AC021534.1 | -2.186963 | -3.237677 | 9.97E-05 | 0.003313 |
| BAAT | -2.199593 | 0.628444 | 1.15E-06 | 8.55E-05 |
| H2AC4 | -2.201009 | -1.767929 | 5.29E-06 | 0.000307 |
| CLDN18 | -2.201671 | 5.085543 | 0.000324 | 0.008181 |
| AC129492.2 | -2.205522 | -3.523571 | 0.000116 | 0.003744 |
| CIB4 | -2.207673 | -2.243641 | 3.97E-06 | 0.000242 |
| STRIT1 | -2.208895 | -2.235449 | 0.000192 | 0.005435 |
| MS4A10 | -2.212048 | 0.466454 | 0.000878 | 0.01713 |
| CRNN | -2.214101 | -3.534558 | 0.002248 | 0.033268 |
| CEACAM20 | -2.254455 | -1.297873 | 2.18E-05 | 0.000992 |
| AC000374.1 | -2.282866 | -3.541518 | 0.00131 | 0.022728 |
| SPRR2B | -2.333273 | -3.583456 | 0.001133 | 0.020688 |
| AC108451.2 | -2.346111 | -3.267799 | 2.41E-05 | 0.001058 |
| CUBN | -2.374366 | 1.884062 | 1.14E-09 | 1.89E-07 |
| DEFA6 | -2.379015 | 5.43261 | 7.54E-06 | 0.000409 |
| RNA5SP149 | -2.384082 | -1.416793 | 0.003569 | 0.045449 |
| GSTA2 | -2.397049 | -0.878581 | 1.46E-06 | 0.000106 |
| KRT14 | -2.410039 | 2.682153 | 0.000537 | 0.011946 |
| SERPINB4 | -2.410331 | -1.550833 | 0.000374 | 0.009073 |
| PRSS1 | -2.466004 | 1.011748 | 1.38E-07 | 1.37E-05 |
| CD177P1 | -2.467866 | -0.430736 | 8.05E-05 | 0.002828 |
| LINC01913 | -2.486482 | -2.036546 | 7.84E-06 | 0.000421 |
| SPRR1B | -2.489457 | 1.31882 | 0.000164 | 0.004858 |
| FP236383.5 | -2.490255 | -2.929003 | 0.001368 | 0.02356 |
| KCNE1B | -2.505455 | -1.49955 | 0.000109 | 0.003574 |
| H4C2 | -2.580639 | -0.803728 | 7.44E-07 | 5.94E-05 |
| RMRP | -2.581062 | -2.867649 | 0.000148 | 0.004474 |
| RNA5SP290 | -2.582737 | -3.26954 | 0.000351 | 0.008661 |
| H4C4 | -2.603776 | 0.14806 | 2.56E-07 | 2.38E-05 |
| PSAPL1 | -2.604102 | -1.540978 | 1.62E-05 | 0.000777 |
| AL355075.4 | -2.61328 | -1.948931 | 1.88E-05 | 0.000878 |
| RNA5SP183 | -2.624123 | -2.540006 | 0.00052 | 0.011681 |
| RNA5S9 | -2.63034 | -1.25553 | 0.001813 | 0.02899 |
| AC099542.1 | -2.645007 | -2.756911 | 7.22E-06 | 0.000396 |
| SPRR2E | -2.66047 | 0.49434 | 0.001622 | 0.026668 |
| SLC10A2 | -2.67242 | 1.085613 | 0.003072 | 0.041353 |
| CYP1A1 | -2.68102 | -0.07836 | 1.24E-05 | 0.000621 |
| H2BC10 | -2.699963 | -1.124098 | 4.07E-06 | 0.000246 |
| RNA5SP473 | -2.721218 | -3.317618 | 2.42E-05 | 0.001061 |
| ORM1 | -2.736362 | 1.478895 | 1.18E-05 | 0.000592 |
| CYP3A4 | -2.749215 | 2.400282 | 2.47E-08 | 3.12E-06 |
| AP002963.1 | -2.754271 | -3.65071 | 9.63E-05 | 0.003237 |
| SEMG2 | -2.754389 | -2.019331 | 1.88E-05 | 0.000878 |
| FGL1 | -2.761677 | -0.909033 | 3.16E-06 | 0.000201 |
| AC092112.1 | -2.782861 | -1.190196 | 2.63E-08 | 3.28E-06 |
| TMEM229A | -2.791739 | -0.739169 | 1.29E-06 | 9.48E-05 |
| KRT78 | -2.793373 | -1.801774 | 1.70E-05 | 0.000809 |
| GP2 | -2.820506 | 3.157725 | 6.81E-07 | 5.51E-05 |
| REG1A | -2.82708 | 9.255213 | 1.28E-07 | 1.30E-05 |
| LCE3D | -2.828001 | -3.401884 | 0.00019 | 0.005406 |
| DEFA5 | -2.872463 | 6.417831 | 1.68E-06 | 0.000119 |
| IGKV3-25 | -2.949202 | -2.820407 | 3.29E-05 | 0.001363 |
| H2BC3 | -3.003325 | -1.179134 | 0.00018 | 0.005213 |
| AC115619.1 | -3.054869 | -2.915593 | 0.00028 | 0.007279 |
| SPRR2C | -3.07673 | -3.192581 | 0.000183 | 0.005279 |
| C8orf34-AS1 | -3.141065 | -2.292146 | 1.09E-06 | 8.29E-05 |
| RNA5SP452 | -3.153241 | -3.253439 | 3.72E-06 | 0.000229 |
| GRM8-AS1 | -3.177182 | -3.03753 | 4.86E-06 | 0.000285 |
| RNA5SP354 | -3.185987 | -2.831767 | 0.000105 | 0.003484 |
| BPIFA1 | -3.208744 | -1.836626 | 0.000131 | 0.004105 |
| SPRR2F | -3.233043 | -0.954902 | 0.000183 | 0.005279 |
| RNA5SP226 | -3.243841 | -2.37489 | 0.000285 | 0.007383 |
| REG3A | -3.27928 | 7.895106 | 4.20E-08 | 4.97E-06 |
| TTC29 | -3.290678 | -0.374067 | 3.26E-06 | 0.000206 |
| CPO | -3.296628 | 0.209404 | 2.19E-09 | 3.31E-07 |
| MSMB | -3.31439 | -1.429806 | 2.58E-07 | 2.39E-05 |
| AC011754.1 | -3.316267 | -1.477669 | 8.34E-06 | 0.000442 |
| CLEC2A | -3.363865 | -2.715696 | 1.76E-05 | 0.000831 |
| H4C3 | -3.415259 | 0.951227 | 5.99E-09 | 8.20E-07 |
| CCL25 | -3.416345 | 2.491814 | 4.44E-08 | 5.19E-06 |
| H1-5 | -3.425499 | 1.73209 | 9.87E-10 | 1.66E-07 |
| PNLIP | -3.432996 | -2.470208 | 0.000362 | 0.008875 |
| PRSS2 | -3.463609 | 6.084782 | 4.20E-08 | 4.97E-06 |
| REG3G | -3.479601 | -0.408644 | 1.21E-06 | 9.04E-05 |
| REG1B | -3.497103 | 6.704275 | 2.88E-08 | 3.57E-06 |
| RNA5-8SP2 | -3.555617 | -2.338924 | 4.81E-05 | 0.001851 |
| RNA5SP145 | -3.571621 | -2.240427 | 3.19E-05 | 0.001332 |
| RNA5SP429 | -3.586083 | -2.964008 | 4.70E-07 | 4.06E-05 |
| RNA5SP259 | -3.591554 | -3.24395 | 1.29E-05 | 0.000641 |
| AL121974.1 | -3.629331 | -2.613459 | 2.23E-05 | 0.001004 |
| RNA5SP150 | -3.788433 | -3.062541 | 1.92E-05 | 0.000892 |
| RNA5SP152 | -3.802797 | -3.210563 | 5.30E-07 | 4.49E-05 |
| APOB | -3.838059 | 4.542439 | 2.28E-07 | 2.14E-05 |
| DLGAP1-AS5 | -3.873747 | 0.371481 | 6.52E-08 | 7.24E-06 |
| CALCB | -3.934653 | 3.052967 | 1.08E-07 | 1.12E-05 |
| RNA5SP481 | -4.003726 | -3.227854 | 2.72E-06 | 0.000177 |
| AC010970.1 | -4.052188 | 1.868737 | 2.97E-09 | 4.36E-07 |
| RNA5SP19 | -4.085669 | -1.625728 | 8.95E-05 | 0.003076 |
| SLC2A2 | -4.124374 | -0.625211 | 3.04E-09 | 4.42E-07 |
| AC152010.1 | -4.144983 | -3.152097 | 8.48E-07 | 6.62E-05 |
| RNA5SP277 | -4.260832 | -2.918084 | 8.13E-07 | 6.41E-05 |
| APOA1 | -4.297577 | 4.184965 | 4.55E-10 | 8.84E-08 |
| UGT2B4 | -4.301581 | -0.249373 | 2.91E-09 | 4.31E-07 |
| INSL4 | -4.346034 | -1.67469 | 4.37E-08 | 5.13E-06 |
| RNA5SP506 | -4.404244 | -2.665944 | 1.74E-06 | 0.000121 |
| RNA5SP431 | -4.425035 | -2.906596 | 3.03E-06 | 0.000195 |
| RNA5SP387 | -4.443245 | -2.383968 | 4.51E-06 | 0.000268 |
| RNA5SP221 | -4.492778 | -1.560945 | 9.77E-08 | 1.02E-05 |
| RNA5-8SP6 | -4.595282 | -1.688262 | 5.43E-08 | 6.21E-06 |
| RNA5SP213 | -4.670778 | -2.568296 | 3.75E-06 | 0.00023 |
| RNA5SP252 | -4.703818 | -3.048881 | 2.13E-07 | 2.02E-05 |
| RNA5SP514 | -4.711732 | -3.124413 | 7.54E-08 | 8.28E-06 |
| RNA5SP486 | -4.765856 | -3.093642 | 8.79E-08 | 9.39E-06 |
| RNA5SP460 | -4.786829 | -2.848509 | 4.12E-07 | 3.59E-05 |
| RNU1-4 | -4.833695 | -2.256804 | 1.92E-06 | 0.000133 |
| RNA5SP202 | -4.855411 | 0.157728 | 5.24E-06 | 0.000305 |
| RNA5SP62 | -4.869175 | -3.031494 | 4.24E-08 | 5.00E-06 |
| RNA5SP99 | -4.880842 | -2.587102 | 7.14E-07 | 5.75E-05 |
| RNA5SP302 | -4.896084 | -2.918386 | 1.36E-07 | 1.36E-05 |
| RNA5SP444 | -4.949873 | -2.976129 | 4.74E-08 | 5.50E-06 |
| RNA5SP243 | -4.973415 | -1.503812 | 2.08E-06 | 0.000142 |
| RNA5SP208 | -5.274311 | -2.000402 | 4.63E-07 | 4.01E-05 |
| APOA4 | -5.320554 | 3.325 | 3.38E-06 | 0.000212 |
| PAEP | -5.341108 | 3.566357 | 5.82E-12 | 1.48E-09 |
| APOC3 | -5.381861 | 1.470024 | 2.08E-07 | 1.98E-05 |
| RNA5SP161 | -5.454934 | -1.275661 | 6.49E-07 | 5.33E-05 |
| RNA5-8SP5 | -5.563051 | -2.549617 | 8.45E-09 | 1.13E-06 |
| RNA5SP191 | -5.60423 | -1.402017 | 1.07E-06 | 8.13E-05 |
| RNA5SP242 | -5.884449 | -1.366477 | 7.06E-08 | 7.78E-06 |
| RNA5SP204 | -5.910595 | -2.29031 | 5.62E-09 | 7.76E-07 |
| RNA5SP225 | -6.032671 | -1.48171 | 1.38E-07 | 1.37E-05 |
| RNA5SP185 | -6.099084 | -2.13131 | 4.89E-09 | 6.81E-07 |
| RNA5SP141 | -6.101402 | 1.031203 | 1.67E-06 | 0.000118 |
| RNA5SP50 | -6.152626 | -1.663321 | 7.80E-08 | 8.53E-06 |
| BX322234.2 | -6.179292 | 0.331893 | 8.22E-09 | 1.10E-06 |
| RNA5SP134 | -6.241846 | -1.509251 | 3.73E-08 | 4.46E-06 |
| RNA5SP215 | -6.297719 | -1.424329 | 3.05E-08 | 3.71E-06 |
| RNA5SP267 | -6.619193 | -1.256408 | 3.80E-08 | 4.53E-06 |
| RNA5SP140 | -6.677614 | -1.629984 | 2.18E-09 | 3.31E-07 |
| RNA5SP211 | -7.101152 | -0.34446 | 6.47E-08 | 7.21E-06 |
| RNA5SP355 | -7.649498 | 0.757362 | 2.16E-08 | 2.74E-06 |
