# Supplementary material for: Integrated Analysis of Distant Metastasis-Associated Genes and Potential Drugs in Colon Adenocarcinoma
Source: Front Oncol. 2020 Oct 23;10:576615. doi: 10.3389/fonc.2020.576615 (PMC7645237; doi:10.3389/fonc.2020.576615)
Supplement: Supplementary Table 2 — Shared genes between the targets of the DEmiRNAs and DEGs. [file Table_2.DOC]

**Table S2** Shared genes between the targets of the DEmiRNAs and DEGs.

| **Gene symbol** | **Log2FC** | **logCPM** | ***p* value** | **FDR** |
| --- | --- | --- | --- | --- |
| TFAP2B | 6.11877 | -1.9252 | 6.31193E-53 | 1.9264E-48 |
| PSG1 | 5.09213 | -3.1907 | 2.26495E-29 | 4.06625E-26 |
| DCAF4L2 | 4.41911 | -2.6566 | 2.9195E-18 | 1.43715E-15 |
| FOXR2 | 3.9917 | -3.6204 | 5.82138E-17 | 2.57491E-14 |
| CHRNA4 | 3.30516 | -2.6881 | 1.76289E-15 | 6.6424E-13 |
| XAGE2 | 3.22863 | -1.8703 | 7.10664E-12 | 1.77782E-09 |
| SALL3 | 3.18994 | -2.6398 | 1.39948E-12 | 4.06783E-10 |
| HRH3 | 3.16435 | -2.5121 | 3.64207E-16 | 1.48208E-13 |
| PSG4 | 3.00332 | -3.4106 | 1.77727E-16 | 7.43045E-14 |
| SLITRK1 | 2.98727 | -3.4329 | 1.13856E-19 | 7.0916E-17 |
| TGM6 | 2.68871 | -2.9919 | 7.081E-11 | 1.53251E-08 |
| UTS2B | 2.68341 | -1.5689 | 7.32245E-27 | 1.0642E-23 |
| SRRM4 | 2.62215 | -2.7262 | 7.03568E-20 | 4.66802E-17 |
| MMD2 | 2.53189 | -3.6309 | 3.48406E-13 | 1.10764E-10 |
| CDH9 | 2.47896 | -2.7675 | 2.88934E-08 | 3.57015E-06 |
| LEP | 2.24394 | -1.1884 | 2.23352E-12 | 6.08635E-10 |
| FBXO40 | 2.22747 | -3.3146 | 1.76463E-09 | 2.79049E-07 |
| ATP2B3 | 2.09736 | -2.2349 | 6.44178E-11 | 1.41441E-08 |
| UGT3A1 | 2.03023 | -2.7822 | 3.10339E-05 | 0.001301037 |
| ANKRD34C | 2.02933 | -3.4987 | 6.69987E-10 | 1.20282E-07 |
| HCN4 | 1.97223 | -2.508 | 1.54199E-15 | 5.8827E-13 |
| DACH2 | 1.93111 | -2.6869 | 9.57485E-12 | 2.35665E-09 |
| SPOCK3 | 1.85095 | -1.837 | 1.31806E-07 | 1.33203E-05 |
| SLC13A5 | 1.76563 | -1.2638 | 1.31702E-12 | 3.86496E-10 |
| DSG1 | 1.71242 | -0.5874 | 2.83433E-19 | 1.63214E-16 |
| SCN1A | 1.70606 | -3.0074 | 1.94928E-07 | 1.85912E-05 |
| CNTNAP4 | 1.58466 | -3.2028 | 3.79553E-05 | 0.001525864 |
| PEX5L | 1.58195 | -2.4379 | 2.33412E-12 | 6.30418E-10 |
| FUT9 | 1.58143 | -2.9709 | 0.000234611 | 0.006330965 |
| AQP4 | 1.50992 | -3.006 | 2.11507E-06 | 0.000143768 |
| UNC80 | 1.49199 | -2.2968 | 7.51425E-09 | 1.01927E-06 |
| CACNG1 | 1.4881 | -3.4833 | 4.80766E-05 | 0.001850951 |
| CLDN19 | 1.45817 | -3.1046 | 9.23537E-06 | 0.000482643 |
| SLC6A2 | 1.45717 | -2.1138 | 0.000298481 | 0.007655168 |
| PCDH11Y | 1.45415 | -3.5652 | 0.000139884 | 0.004321108 |
| SLC6A15 | 1.45234 | -1.2113 | 2.1872E-05 | 0.0009921 |
| SLC7A14 | 1.45022 | -1.2668 | 1.94484E-05 | 0.000899339 |
| FOXG1 | 1.45015 | -1.4548 | 0.000652476 | 0.013886729 |
| PCSK2 | 1.43512 | -0.2544 | 0.000139725 | 0.004320584 |
| CXCL5 | 1.4346 | 5.15362 | 4.68786E-06 | 0.000277274 |
| LHX9 | 1.42592 | -2.9969 | 4.61742E-05 | 0.001795206 |
| PLD5 | 1.3957 | -3.0121 | 7.18276E-06 | 0.000394987 |
| PCDHA9 | 1.38742 | -3.4652 | 2.34679E-05 | 0.001039536 |
| MGAT5B | 1.38708 | -1.6607 | 1.16457E-11 | 2.79864E-09 |
| GTSF1 | 1.37871 | 0.29166 | 5.02856E-06 | 0.000294007 |
| CLSTN2 | 1.37864 | 2.25623 | 2.95587E-09 | 4.35533E-07 |
| KL | 1.36874 | 0.60907 | 1.20231E-13 | 4.03236E-11 |
| C12orf40 | 1.36271 | -3.4883 | 0.000122146 | 0.003895795 |
| WIPF3 | 1.35118 | 1.54195 | 8.37772E-10 | 1.46947E-07 |
| ATCAY | 1.34931 | -1.5518 | 1.69337E-06 | 0.000119082 |
| DLX2 | 1.34792 | -1.5734 | 6.6933E-07 | 5.46202E-05 |
| FAM133A | 1.32433 | -2.9269 | 1.73732E-05 | 0.000825531 |
| DLX1 | 1.30957 | -1.1248 | 5.04621E-06 | 0.000294475 |
| CSMD3 | 1.30485 | -2.3309 | 3.34073E-05 | 0.001377824 |
| PCDHGB7 | 1.29055 | 1.38436 | 3.8543E-13 | 1.21271E-10 |
| POU6F2 | 1.29008 | 0.91385 | 0.000409311 | 0.009797794 |
| GBX2 | 1.28357 | -1.3608 | 4.26866E-06 | 0.000256456 |
| SH3GL3 | 1.23983 | -3.35 | 0.000522096 | 0.01171645 |
| SYT16 | 1.23197 | -3.0845 | 1.71436E-06 | 0.000120005 |
| KCNN1 | 1.22096 | -1.7959 | 3.47811E-10 | 6.84851E-08 |
| KCNJ6 | 1.22014 | -1.9271 | 1.07276E-07 | 1.10985E-05 |
| JPH3 | 1.21863 | -0.9943 | 4.57604E-08 | 5.33056E-06 |
| PLIN1 | 1.21493 | 1.32019 | 5.30692E-06 | 0.000307922 |
| CACNG4 | 1.20281 | 2.2797 | 0.000130169 | 0.004082986 |
| GABRG2 | 1.20034 | -2.2404 | 0.000868795 | 0.017051857 |
| ASTN1 | 1.17833 | -1.5 | 0.000293828 | 0.007567624 |
| SERPINB2 | 1.15983 | 0.69404 | 0.001178018 | 0.021261452 |
| SCEL | 1.15382 | 1.72885 | 6.96742E-05 | 0.002519497 |
| SPTSSB | 1.12569 | 0.5666 | 3.33944E-05 | 0.001377824 |
| S100Z | 1.12398 | -2.7387 | 3.16754E-08 | 3.82108E-06 |
| DGKB | 1.11931 | 0.1391 | 0.00041102 | 0.009827704 |
| ERBB4 | 1.10287 | -2.862 | 0.000346045 | 0.008573517 |
| ALX4 | 1.07397 | -2.9639 | 0.000107933 | 0.003542065 |
| ATP2B2 | 1.07089 | -2.356 | 2.30429E-05 | 0.001028172 |
| DUSP9 | 1.05109 | -0.2397 | 0.000139578 | 0.004320409 |
| SVOP | 1.04964 | -1.0825 | 0.000361533 | 0.008862633 |
| ELFN2 | 1.04269 | 0.43007 | 4.95168E-05 | 0.001893799 |
| MYO3A | 1.02636 | -1.5143 | 0.000208517 | 0.005776716 |
| GABRA4 | 1.02289 | 0.54122 | 0.001621504 | 0.026667913 |
| ANGPTL5 | 1.01953 | -3.1594 | 0.000341124 | 0.00847811 |
| DLGAP1 | 1.01131 | -0.4582 | 3.83558E-06 | 0.000234593 |
| ZNF365 | 1.00531 | -1.2274 | 2.43899E-06 | 0.000160465 |
| CALML3 | -1.0154 | -0.0853 | 0.002903235 | 0.039724856 |
| TRIM7 | -1.0182 | 3.08217 | 0.000383518 | 0.009245627 |
| ENPEP | -1.0224 | 3.23953 | 7.16712E-05 | 0.002576448 |
| UROC1 | -1.0299 | -2.865 | 0.002149817 | 0.032337318 |
| UGT2B7 | -1.0319 | 2.78485 | 0.000580862 | 0.01272643 |
| ABCG5 | -1.0354 | 0.37972 | 0.000508038 | 0.011489916 |
| RFPL2 | -1.0533 | -2.863 | 0.004046123 | 0.049620578 |
| FASLG | -1.0558 | 0.02814 | 1.45548E-06 | 0.000105765 |
| SMLR1 | -1.0736 | -2.2643 | 0.001183067 | 0.021287789 |
| CYP2C9 | -1.1021 | 0.05378 | 0.000442633 | 0.010343919 |
| CXCL11 | -1.1556 | 4.17325 | 4.69887E-05 | 0.00182223 |
| HSF5 | -1.1644 | -2.5352 | 0.00197496 | 0.030636412 |
| C2CD4B | -1.1717 | 3.12409 | 4.94037E-06 | 0.000289405 |
| TCHH | -1.1749 | 0.28019 | 0.00098029 | 0.018594441 |
| SERPINA1 | -1.2966 | 8.74101 | 2.9472E-07 | 2.63008E-05 |
| GBP5 | -1.3007 | 3.85724 | 1.274E-07 | 1.29608E-05 |
| MUC5B | -1.3192 | 8.49872 | 5.17768E-05 | 0.001971377 |
| MYBPC3 | -1.3208 | -0.8117 | 6.34011E-06 | 0.000355691 |
| SBSN | -1.3391 | -1.1726 | 0.000853961 | 0.016897366 |
| ALDH1A2 | -1.3425 | 2.23795 | 0.00051582 | 0.011609743 |
| HABP2 | -1.5041 | 1.04439 | 0.000970137 | 0.018493799 |
| REG4 | -1.5056 | 9.28986 | 0.00017661 | 0.005158025 |
| CXCL9 | -1.5525 | 5.28176 | 6.04623E-09 | 8.23799E-07 |
| PCSK1 | -1.5852 | 5.10095 | 0.000148334 | 0.004486785 |
| KIF19 | -1.602 | 1.97049 | 2.26763E-06 | 0.000151109 |
| PGLYRP3 | -1.6065 | -2.2165 | 0.000857793 | 0.01690112 |
| GPR83 | -1.6158 | -0.3471 | 2.77622E-05 | 0.001183382 |
| CRP | -1.6795 | -3.0205 | 0.00245378 | 0.035275251 |
| ENPP7 | -1.7601 | -2.0165 | 8.74087E-05 | 0.003014364 |
| IDO1 | -1.7653 | 4.76942 | 1.01153E-07 | 1.05725E-05 |
| PCA3 | -1.7725 | -2.0265 | 0.000108445 | 0.003555047 |
| KLK12 | -1.7779 | 2.66214 | 1.77454E-06 | 0.000123089 |
| MUC5AC | -1.8947 | 5.75286 | 3.84753E-05 | 0.001541034 |
| IVL | -2.1142 | -1.0064 | 0.00359406 | 0.045647398 |
| CLDN18 | -2.2017 | 5.08554 | 0.000323826 | 0.008181438 |
| CIB4 | -2.2077 | -2.2436 | 3.96653E-06 | 0.000242117 |
| SPRR2B | -2.3333 | -3.5835 | 0.001132863 | 0.020687817 |
| PSAPL1 | -2.6041 | -1.541 | 1.61817E-05 | 0.000776517 |
| SPRR2E | -2.6605 | 0.49434 | 0.001621745 | 0.026667913 |
| SLC10A2 | -2.6724 | 1.08561 | 0.003071651 | 0.041352795 |
| SEMG2 | -2.7544 | -2.0193 | 1.88247E-05 | 0.000878488 |
| REG3A | -3.2793 | 7.89511 | 4.1981E-08 | 4.97024E-06 |
| REG3G | -3.4796 | -0.4086 | 1.21382E-06 | 9.03553E-05 |
| UGT2B4 | -4.3016 | -0.2494 | 2.91221E-09 | 4.31459E-07 |
| PAEP | -5.3411 | 3.56636 | 5.8231E-12 | 1.48101E-09 |
