# Supplementary material for: Integrated Analysis of Distant Metastasis-Associated Genes and Potential Drugs in Colon Adenocarcinoma
Source: Front Oncol. 2020 Oct 23;10:576615. doi: 10.3389/fonc.2020.576615 (PMC7645237; doi:10.3389/fonc.2020.576615)
Supplement: Supplementary Table 3 — Gene Ontology (GO) term enrichment for the prognosis-associated genes differentially expressed in distant metastatic COAD. [file Table_3.DOC]

**TABLE S3** Gene Ontology (GO) term enrichment for the prognosis-associated genes differentially expressed in distant metastatic COAD.

| **Gene** | **GO class (direct)** | **Evidence** | **Evidence with** | **Reference** | |
| --- | --- | --- | --- | --- | --- |
| *LEP* | Tyrosine phosphorylation of STAT protein | IBA | MGI:MGI:104663 | PMID:21873635 |  |
|  | Positive regulation of receptor signaling pathway via JAK-STAT | IBA | UniProtKB:P41159 | PMID:21873635 | |
|  | Positive regulation of phosphatidylinositol 3-kinase signaling | IBA | UniProtKB:P41159 | PMID:21873635 | |
|  | Negative regulation of appetite by leptin-mediated signaling pathway | IBA | PANTHER:PTN001711963 | PMID:21873635 | |
|  | Response to insulin | IBA | UniProtKB:G8BLB4 UniProtKB:P50595 | PMID:21873635 | |
|  | positive regulation of TOR signaling | IBA | UniProtKB:P41159 | PMID:21873635 | |
|  | Activation of protein kinase C activity | IBA | UniProtKB:P41159 | PMID:21873635 | |
|  | Extracellular space | IBA | UniProtKB:G8BLB4 UniProtKB:O42164 UniProtKB:P50595 | PMID:21873635 | |
|  | Positive regulation of p38MAPK cascade | IBA | UniProtKB:P41159 | PMID:21873635 | |
| *DLX2* | RNA polymerase II cis-regulatory region sequence-specific DNA binding | IBA | UniProtKB:Q92988 | PMID:21873635 | |
|  | Regulation of transcription by RNA polymerase II | IBA | UniProtKB:O95231 UniProtKB:P56177 UniProtKB:Q92988 UniProtKB:Q9H9S0 | PMID:21873635 | |
|  | Cell differentiation | IBA | PANTHER:PTN000675745 | PMID:21873635 | |
|  | Positive regulation of transcription by RNA polymerase II | IBA | UniProtKB:P56177 | PMID:21873635 | |
|  | Negative regulation of transcription by RNA polymerase II | IBA | PANTHER:PTN002802895 | PMID:21873635 | |
|  | Nucleus | IBA | UniProtKB:A7Y7W3 UniProtKB:P56177 UniProtKB:Q6NSW7 UniProtKB:Q9H9S0 | PMID:21873635 | |
|  | DNA-binding transcription factor activity | IBA | UniProtKB:Q9H9S0 | PMID:21873635 | |
|  | Sequence-specific DNA binding | IBA | UniProtKB:O95231 UniProtKB:Q92988 UniProtKB:Q9H9S0 | PMID:21873635 | |
| *CLSTN2* | Golgi membrane | IEA | UniProtKB-SubCell:SL-0134 | GO_REF:0000044 | |
|  | Endoplasmic reticulum membrane | IEA | UniProtKB-SubCell:SL-0097 | GO_REF:0000044 | |
|  | Homophilic cell adhesion via plasma membrane adhesion molecules | IEA | InterPro:IPR002126 | GO_REF:0000002 | |
|  | Dendrite | IEA | UniProtKB-SubCell:SL-0283 | GO_REF:0000044 | |
|  | Glutamatergic synapse | IEA | UniProtKB:Q9ER65 | GO_REF:0000107 | |
|  | Integral component of postsynaptic density membrane | IEA | UniProtKB:Q9ER65 | GO_REF:0000107 | |
|  | Postsynaptic membrane | IBA | PANTHER:PTN001020450 | PMID:21873635 | |
|  | Positive regulation of synapse assembly | IBA | PANTHER:PTN001020450 | PMID:21873635 | |
|  | Positive regulation of synaptic transmission | IBA | PANTHER:PTN001020450 | PMID:21873635 | |
|  | Cell surface | IBA | PANTHER:PTN001020450 | PMID:21873635 | |
| *REG3A* | Protein binding | IPI | UniProtKB:Q99750 | PMID:25416956 | |
|  | Extracellular region | TAS |  | Reactome:R-HSA-6801762 | |
|  | Cytoplasm | TAS |  | PMID:8997243 | |
|  | Acute-phase response | IEA | UniProtKB-KW:KW-0011 | GO_REF:0000043 | |
|  | Heterophilic cell-cell adhesion via plasma membrane cell adhesion molecules | TAS |  | PMID:8997243 | |
|  | Positive regulation of keratinocyte proliferation | ISS | UniProtKB:O09037 | GO_REF:0000024 | |
|  | Carbohydrate binding | TAS |  | PMID:1325291 | |
|  | Identical protein binding | IPI | UniProtKB:Q06141 | PMID:24256734 | |
|  | Negative regulation of keratinocyte differentiation | ISS | UniProtKB:O09037 | GO_REF:0000024 | |
|  | Positive regulation of wound healing | ISS | UniProtKB:O09037 | GO_REF:0000024 | |
|  | Signaling receptor activity | IBA | UniProtKB:Q13018 | PMID:21873635 | |
|  | Positive regulation of cell population proliferation | IBA | PANTHER:PTN000514573 | PMID:21873635 | |
|  | Cell wall disruption in other organism | IBA | UniProtKB:Q6UW15 | PMID:21873635 | |
|  | Peptidoglycan binding | IBA | UniProtKB:Q6UW15 | PMID:21873635 | |
|  | Oligosaccharide binding | IBA | UniProtKB:Q6UW15 | PMID:21873635 | |
|  | Response to peptide hormone | IBA | PANTHER:PTN000514573 | PMID:21873635 | |
|  | Antimicrobial humoral immune response mediated by antimicrobial peptidede | IBA | UniProtKB:Q6UW15 | PMID:21873635 | |
|  | Extracellular space | IBA | PANTHER:PTN000514573 | PMID:21873635 | |

ISS sequence similarity evidence used in manual assertion, IBA biological aspect of ancestor evidence used in manual assertion, TAS traceable author statement used in manual assertion, IEA evidence used in automatic assertion, IPI physical interaction evidence used in manual assertion
