# Supplementary material for: Integrated Analysis of Distant Metastasis-Associated Genes and Potential Drugs in Colon Adenocarcinoma
Source: Front Oncol. 2020 Oct 23;10:576615. doi: 10.3389/fonc.2020.576615 (PMC7645237; doi:10.3389/fonc.2020.576615)
Supplement: Supplementary Table 4 — The relationship between cancers and prognosis-associated genes differentially expressed in distant metastatic COAD based on CTD. [file Table_4.DOC]

**TABLE S4** The relationship between cancers and prognosis-associated genes differentially expressed in distant metastatic COAD based on CTD.

| **Genes** | **Disease** | **Inference Score** | **Direct Evidence** |
| --- | --- | --- | --- |
| *LEP* | Breast Neoplasms | 152.21 | * |
|  | Colonic Neoplasms | 98.62 | * |
|  | Prostatic Neoplasms | 78.52 | * |
|  | Polycystic Ovary Syndrome | 46.21 | * |
| *DLX2* | Lung Neoplasms | 71.41 |  |
|  | Cell Transformation, Neoplastic | 71.08 |  |
|  | Neoplasms | 60.61 |  |
|  | Precancerous Conditions | 52.04 |  |
|  | Breast Neoplasms | 48.76 |  |
|  | Ovarian Neoplasms | 45.96 |  |
|  | Neoplasms, Experimental | 44.07 |  |
|  | Adenocarcinoma | 42.97 |  |
|  | Uterine Cervical Neoplasms | 41.28 |  |
|  | Liver Neoplasms | 40.14 |  |
|  | Liver Neoplasms, Experimental | 39.26 |  |
|  | Carcinoma, Squamous Cell | 38.4 |  |
|  | Neoplasm Invasiveness | 35.47 |  |
|  | Skin Neoplasms | 34.58 |  |
|  | Prostatic Neoplasms | 31.45 |  |
|  | Urinary Bladder Neoplasms | 30.59 |  |
|  | Pancreatic Neoplasms | 30.26 |  |
|  | Carcinoma, Hepatocellular | 27.24 |  |
|  | Colonic Neoplasms | 26.12 |  |
| *CLSTN2* | Liver Neoplasms | 63.58 |  |
|  | Lung Neoplasms | 68.11 |  |
|  | Liver Neoplasms, Experimental | 63.12 |  |
|  | Cell Transformation, Neoplastic | 61.73 |  |
|  | Carcinoma, Hepatocellular | 54.45 |  |
|  | Neoplasms, Experimental | 51.3 |  |
|  | Adenoma | 48.98 |  |
|  | Neoplasms | 48.08 |  |
|  | Breast Neoplasms | 42.63 |  |
|  | Precancerous Conditions | 42.2 |  |
|  | Uterine Cervical Neoplasms | 38 |  |
|  | Adenocarcinoma | 37.42 |  |
|  | Adenoma, Liver Cell | 31.64 |  |
|  | Thyroid Neoplasms | 29.28 |  |
|  | Mammary Neoplasms, Animal | 29.2 |  |
|  | Urinary Bladder Neoplasms | 28.87 |  |
|  | Colonic Neoplasms | 28.04 |  |
|  | Prostatic Neoplasms | 28.03 |  |
|  | Neoplasm Invasiveness | 26.8 |  |
|  | Neoplasm Metastasis | 26.14 |  |
| *REG3A* | Liver Neoplasms | 44.4 |  |
|  | Carcinoma, Hepatocellular | 43.41 |  |
|  | Neoplasms, Experimental | 42.5 |  |
|  | Neoplasms | 40.28 |  |
|  | Liver Neoplasms, Experimental | 36.9 |  |
|  | Ovarian Neoplasms | 36.51 |  |
|  | Neoplasm Metastasis | 36.18 |  |
|  | Breast Neoplasms | 35.96 |  |
|  | Uterine Cervical Neoplasms | 34.72 |  |
|  | Lung Neoplasms | 33.04 |  |
|  | Multiple Myeloma | 30.51 |  |
|  | Precancerous Conditions | 30.17 |  |
|  | Carcinoma | 29.91 |  |
|  | Prostatic Neoplasms | 29.02 |  |
|  | Colonic Neoplasms | 28.62 |  |
|  | Adenocarcinoma | 25.13 |  |
|  | Mammary Neoplasms, Experimental | 25.09 |  |
