# Supplementary material for: Integrated Analysis of Distant Metastasis-Associated Genes and Potential Drugs in Colon Adenocarcinoma
Source: Front Oncol. 2020 Oct 23;10:576615. doi: 10.3389/fonc.2020.576615 (PMC7645237; doi:10.3389/fonc.2020.576615)
Supplement: Supplementary Table 5 — Relationship between cancers and three potential drugs for COAD based on CTD. [file Table_5.DOC]

**TABLE S5** Relationship between cancers and three potential drugs for COAD based on CTD.

| **Drugs** | **Disease** | **Inference Score** | **Direct Evidence** |
| --- | --- | --- | --- |
| Ajmaline | Osteosarcoma | 3.52 |  |
|  | Cell Transformation, Neoplastic | 3.17 |  |
|  | Colonic Neoplasms | 3.15 |  |
|  | Neoplasms | 3.05 |  |
|  | Prostatic Neoplasms | 2.5 |  |
|  | Breast Neoplasms | 2.47 |  |
| TTNPB | Breast Neoplasms | 30.4 |  |
|  | Carcinoma, Hepatocellular | 27.17 |  |
|  | Prostatic Neoplasms | 22.59 |  |
|  | Esophageal Neoplasms | 19.72 |  |
|  | Carcinoma | 18.91 |  |
|  | Mammary Neoplasms, Animal | 17.71 |  |
|  | Liver Neoplasms | 15.67 |  |
|  | Adenocarcinoma | 15.38 |  |
|  | Mammary Neoplasms, Experimental | 15.36 |  |
|  | Intestinal Neoplasms | 15.23 |  |
|  | Squamous Cell Carcinoma of Head and Neck | 14.84 |  |
|  | Barrett Esophagus | 13.92 |  |
|  | Carcinoma, Squamous Cell | 13.52 |  |
|  | Lung Neoplasms | 11.67 |  |
|  | Colonic Neoplasms | 11.47 |  |
|  | Polycystic Ovary Syndrome | 11.18 |  |
|  | Colorectal Neoplasms | 11.09 |  |
| Dydrogesterone | Meningioma | 4.76 | * |
|  | Endometrial Neoplasms | 17.07 |  |
|  | Neoplasms, Hormone-Dependent | 17.03 |  |
|  | Cell Transformation, Neoplastic | 16.89 |  |
|  | Polycystic Ovary Syndrome | 15.36 |  |
|  | Leukemia-Lymphoma, Adult T-Cell | 15.04 |  |
|  | Mesothelioma, Malignant | 11.93 |  |
|  | Breast Neoplasms | 11.7 |  |
|  | Lung Neoplasms | 10.67 |  |
|  | Prostatic Neoplasms | 9.24 |  |
|  | Stomach Neoplasms | 8.3 |  |
|  | Sarcoma | 8.19 |  |
|  | Mesothelioma | 7.99 |  |
|  | Thyroid Neoplasms | 7.17 |  |
|  | Carcinoma, Hepatocellular | 6.86 |  |
|  | Carcinoma, Non-Small-Cell Lung | 6.68 |  |
|  | Neoplasms, Hormone-Dependent | 6.55 |  |
|  | Mammary Neoplasms, Experimental | 6.12 |  |
|  | Adenocarcinoma | 5.94 |  |
|  | Tuberous Sclerosis 2 | 5.6 |  |
|  | Colonic Neoplasms | 5.59 |  |
